# Toward Anti‐Herpesviral PROTACs: Assessing the Challenges for Targeted Protein Degradation on the Example of Kaposi's Sarcoma‐Associated Herpesvirus Latency‐Associated Nuclear Antigen

**DOI:** 10.1002/cmdc.202500758

**Published:** 2025-11-18

**Authors:** Aylin Berwanger, Saskia Catherina Stein, Sarah Brandner, Andreas Martin Kany, Sebastian Heinz, Brigitta Loretz, Claus‐Michael Lehr, Anna Katharina Herta Hirsch, Frederik Lermyte, Thomas Friedrich Schulz, Martin Empting

**Affiliations:** ^1^ Helmholtz Centre for Infection Research (HZI)/Helmholtz‐Institute for Pharmaceutical Research Saarland (HIPS) Campus E8.1 66123 Saarbrücken Germany; ^2^ Department of Pharmacy Saarland University Campus E8.1 66123 Saarbrücken Germany; ^3^ PharmaScienceHub (PSH) Saarland University Campus E2.1 66123 Saarbrücken Germany; ^4^ German Centre for Infection Research (DZIF) Partner Site Hannover‐Braunschweig, 66123 Saarbrücken Germany; ^5^ Hannover Medical School Institute of Virology Carl‐Neuberg‐Str.1 30625 Hannover Germany; ^6^ Cluster of Excellence RESIST (EXC 2155) Hannover Medical School Carl‐Neuberg‐Str.1 30625 Hannover Germany; ^7^ Department of Chemistry Technical University of Darmstadt Peter‐Grünberg‐Str.4 64287 Darmstadt Germany

**Keywords:** Kaposi's sarcoma‐associated herpesvirus, latency‐associated nuclear antigen, native mass spectrometry, proteolysis targeting chimeras, ternary complex formation

## Abstract

Kaposi's sarcoma‐associated herpesvirus (KSHV) is a gamma‐herpesvirus linked to several malignancies, including Kaposi's sarcoma, primary effusion lymphoma, and multicentric Castleman's disease. Among its numerous encoded proteins, the latency‐associated nuclear antigen (LANA) plays a pivotal role in the maintenance of viral latency and oncogenesis. This manuscript focuses on therapeutic strategies aimed at targeting LANA to prevent KSHV‐associated diseases. Following the concept of proteolysis‐targeting chimeras (PROTACs), heterobifunctional compounds are designed and synthesized, which are able to bind LANA as well as specific E3 ligases. To achieve induction of targeted protein degradation, formation of functional ternary complexes as well as uptake into cells is required, which necessitates optimization of multiple compound parameters in parallel. Hence, the conjugates are tested using an assay pipeline tailored for PROTAC drug discovery by checking properties such as binding affinities, formation of ternary complexes, and in vitro absorption, distribution, metabolism, excretion (ADME) data. Restricted permeation as the reason for lack of intracellular target degradation is especially identified.

## Graphical abstract

It is attempted to employ proteolysis‐targeting chimeras‐based targeted protein degradation to the viral protein “Latency‐associated Nuclear Antigen” from Kaposi's sarcoma‐associated herpesvirus. While ternary complex formation is detected via native mass spectrometry, protein degradation is not observed, presumably due to insufficient permeability.

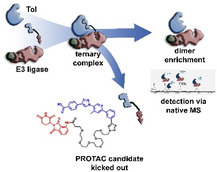

## 1 Introduction

Kaposi's sarcoma‐associated herpesvirus (KSHV), also known as human gamma‐herpesvirus 8 (HHV‐8), is a member of the herpesvirus family.^[^ ^1^ ^]^ It plays a significant role in the development of Kaposi's sarcoma, a cancer commonly observed in individuals with AIDS.^[^ ^2^ ^]^ KSHV exhibits a dual life cycle, characterized by latent and lytic phases.^[^ ^3^ ^]^ During latent infection, KSHV persists within host cells, maintaining its genome as an extrachromosomal episome.^[^ ^1^ ^]^ The latency‐associated nuclear antigen (LANA) is a pivotal player in this phase as it ensures segregation of the viral genome during cell division.^[^ ^3^ ^,^ ^4^ ^]^ LANA not only maintains genome stability but also broadly regulates viral gene expression, making it a critical target for therapeutic strategies aimed at abolishing KSHV infection.^[^ ^3^ ^]^ The lytic phase involves active viral replication and production of infectious particles. High levels of lytic gene expression are dependent on LANA's presence during this phase.^[^ ^5^ ^]^

**Figure 1:**
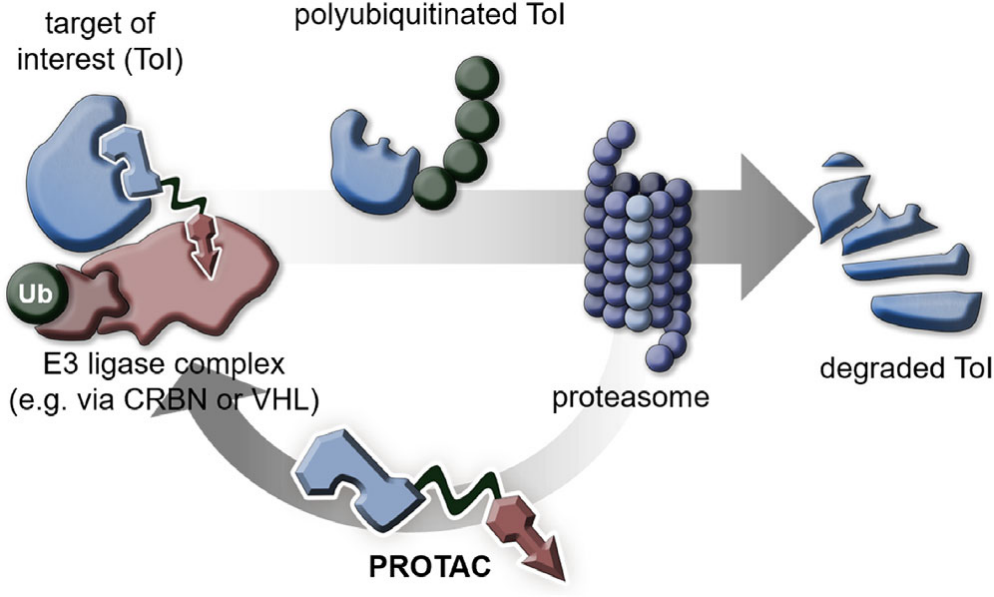
Concept of proteolysis targeting chimeras.

In previous studies, we have demonstrated that inhibition of the LANA–DNA interaction by small molecular entities is a viable antiviral strategy.^[^ ^6^ ^–^ ^8^ ^]^ In light of these results, attempting to identify new drug modalities that rely on targeted protein degradation (TPD) of the KSHV LANA protein appears to be very attractive.^[^ ^9^ ^]^ This innovative approach enables the specific removal of, e.g., a disease‐mediating (viral) protein from host cells.^[^ ^10^, ^11^ ^–^ ^12^ ^]^ In the context of TPD, so‐called proteolysis‐targeting chimeras (PROTACs) are one of the most prominent modalities.^[^ ^13^ ^–^ ^15^ ^]^ They consist of two functional domains: one that binds to the target protein (target of interest; ToI) and another that recruits an E3 ubiquitin ligase.^[^ ^14^ ^]^ The E3 ligase is able to tag the target protein with ubiquitin, marking it for proteasomal degradation.^[^ ^16^ ^]^ This concept is illustrated in **Figure** **1**. PROTACs offer a promising avenue for treating diseases by specifically removing individual proteins of interest from the cellular environment.

## 2 Results and Discussion

### 2.1 Design of PROTAC Conjugates

**Figure 2:**
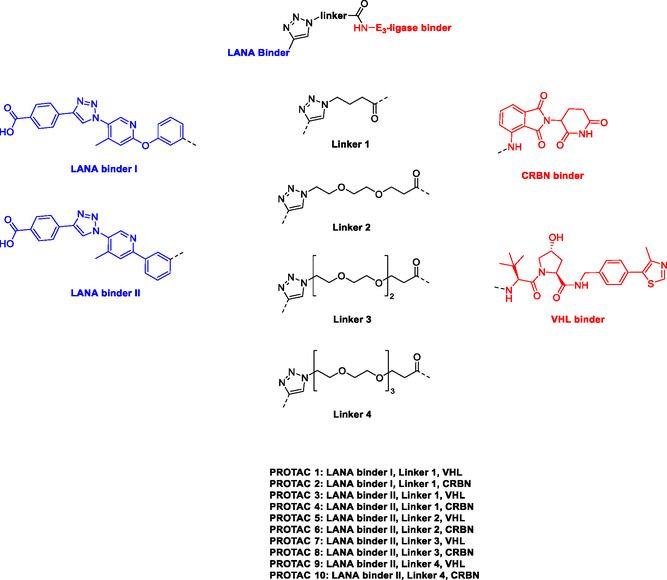
Design of LANA‐binding PROTACs with small molecule inhibitor part in blue, E3 ligase ligands in red, and linker in black.

The design of LANA‐degrading PROTACs is based on two previously identified small molecule inhibitors (**Figure** **2**, LANA binder I and LANA binder II shown in blue).^[^ ^7^ ^,^ ^8^ ^]^ LANA binder I was chosen initially due to its more flexible ether linkage within the phenoxy motif, with the aim of promoting ternary complex formation. Considering our initial results, we obtained for our PROTACs **1** and **2**, we shifted our focus to LANA binder II. This was supported by the previously reported capability of this latter scaffold to inhibit LANA‐driven replication in a cell‐based assay.^[^ ^8^ ^]^ Additionally, chemical accessibility of LANA binder II was superior, granting more facile synthesis. For LANA binder I, a microwave reaction was required, which could only be carried out on a small scale. In contrast, the synthesis of LANA binder II could be conducted on a larger scale. Additionally, when comparing PROTACs **1** and **2** with PROTACs **3** and **4**, we observed higher inhibition and binding affinity for the PROTACs based on LANA binder II (see **Table** **1**).

**Table 1:** Synthesized PROTACs and their K_D_ values determined via MST as well as inhibition via EMSA at a compound concentration of 50 µM.

PROTAC number	Structure	K_D_ [MST] [µM]a)	Inhibition [EMSA, LBS1] @50 µMa)
1	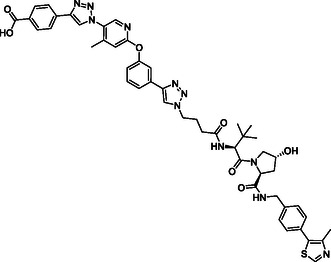 LANA binder I—Linker 1—VHL	45 ± 8.9	23 ± 7%
2	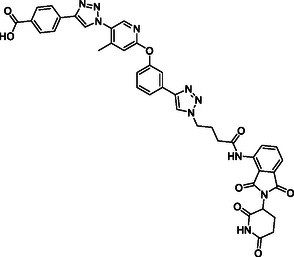 LANA binder I—Linker 1—CRBN	5.9 ± 0.5	30 ± 10%
3	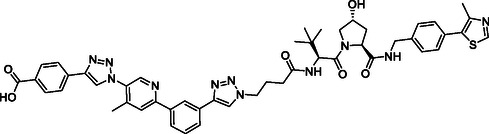 LANA binder II—Linker 1—VHL	6.4 ± 2.5	24 ± 1%
4	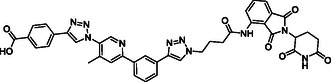 LANA binder II—Linker 1—CRBN	1.6 ± 0.3	59 ± 2%
5	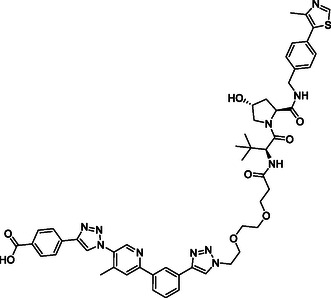 LANA binder II—Linker 2—VHL	1.3 ± 0.5	1 ± 1%
6	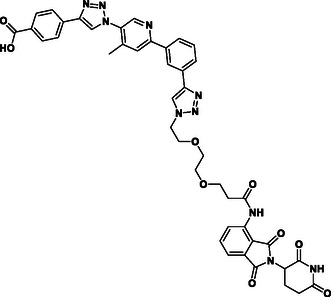 LANA binder II—Linker 2—CRBN	6.1 ± 1.6	n.i.
7	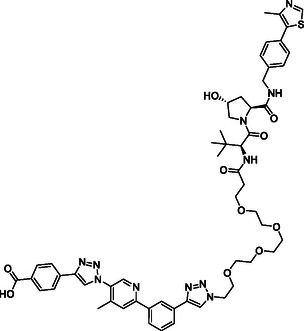 LANA binder II—Linker 3—VHL	12 ± 1.9	19 ± 7%
8	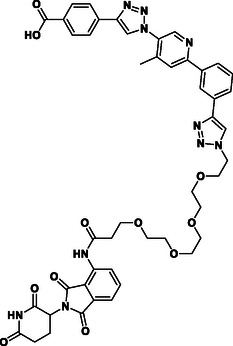 LANA binder II—Linker 3—CRBN	17 ± 2.9	2 ± 2%
9	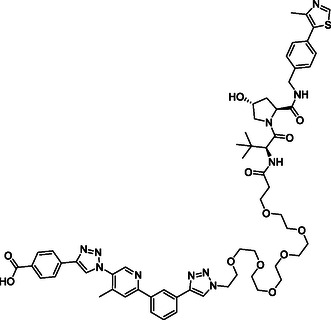 LANA binder II—Linker 4—VHL	16 ± 1.5	4 ± 4%
10	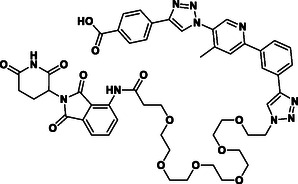 LANA binder II—Linker 4—CRBN	14 ± 1.8	2 ± 2%

n.i. = no inhibition,

a) values are means of at least three replicates, and ± values represent standard deviation.

As E3 ligase binders, we used either the cereblon (CRBN) ligand pomalidomide or the Von–Hippel–Lindau (VHL) ligand (*S, R,S*)‐AHPC (Figure 2, shown in red).

We decided to use flexible linkers (mainly PEG‐based), as this is a known strategy to improve aqueous solubility while maintaining permeability.^[^ ^17^ ^,^ ^18^ ^]^ The linkers are drawn in black color.

### 2.2 Biological Evaluation

We tested our PROTAC candidates step by step using a pipeline with different assays to sequentially check the most important properties. PROTACs exceed the structural space of Lipinski's rule of five and often require optimization of physicochemical properties to be functional. Hence, in addition to effective binding to the target of interest, important features are permeability, capability to induce formation of the ternary complex, as well as subsequent ubiquitination and degradation. In a first step, the PROTAC candidates were tested in two different assays for their binding affinity to LANA as well as for their inhibition of the LANA–DNA interaction. For the determination of the binding affinity, we used microscale thermophoresis (MST) employing an oligomerization‐deficient mutant of the LANA *C*‐terminal domain (amino acid residues 1008–1146). An electrophoretic mobility shift assay (EMSA) was performed for the determination of the inhibition of the protein–DNA interaction at 50 µM compound concentration by using the same truncated and oligomerization‐deficient LANA construct and an oligonucleotide representing the LANA binding site (LBS) 1.^[^ ^6^ ^]^ The observed K_D_ values and LANA inhibition by the PROTACs are shown in Table 1.

All PROTACs were able to bind LANA, the protein of interest, with most of them having K_D_ values in the low micromolar range, which is sufficient for the PROTAC concept, which relies on a catalytic “event‐driven” mechanism of action (MoA) as opposed to the classical “occupancy‐driven” paradigm for drug discovery.^[^ ^19^ ^]^ Inhibition by PROTAC candidates was tested at a concentration of 50 µM to enable direct comparison as compounds **1–5** were not soluble at higher concentrations (see **Table** **2**). The PROTAC candidates showed low to moderate inhibition of the LANA–DNA interaction at this concentration. The highest inhibition was observed for compound **4** (59%). This compound also showed a good binding affinity of 1.6 ± 0.3 µM. As visible in Table 1, there is no correlation between affinity and inhibition. This could be due to a shift of binding sites away from the protein‐DNA interface. The binding to LANA is essential for PROTACs, but the inhibition is not, as long as a ternary complex is formed successfully.

**Table 2:** In vitro ADME data.

Compound	Kinetic solubility 1% DMSO/PBS [µM]a)	Chrom. LogD_*7.4*_	Cell permeability P_app_ Caco‐2 [10^−6^ cm s^−1^]	Mouse liver S9, t_1/2_ [ min]/Cl_int_ [µL mg^−1^ min^−1^]a)	Mouse liver microsomes, t_1/2_ [ min]/Cl_int_ [µL mg^−1^ min^−1^]a)	Human liver S9, t_1/2_ [min]/Cl_int_ [µL mg^−1^ min^−1^]a)
1	51 ± 6.3	0.91	n.p.	67 ± 13/ 10.6 ± 2.1	64 ± 9.0/ 21.8 ± 3.0	>120/ <5.8
2	41 ± 17	0.78	n.p	>120/ <5.8	n.d.	>120/ <5.8
3	44 ± 2.1	0.83	n.p	>120/ <5.8	42 ± 15/ 36 ± 13	>120/ <5.8
4	54 ± 2.6	0.61	n.p	>120/ <5.8	n.d.	>120/ <5.8
5	45 ± 22	0.78	n.p	24.3 ± 2.5/ 27.8 ± 2.9	18.9 ± 3.0/ 75 ± 12	98 ± 34/ 7.6 ± 2.6
6	>200	0.53	n.p	21.4 ± 2.3/ 32.7 ± 3.5	11.1 ± 1.0/ 126 ± 11	105 ± 8/ 6.7 ± 0.5
7	>100	0.83	n.p	5.0 ± 1.0/ 144 ± 30	4.2 ± 0.1/ 332 ± 9.2	25.5 ± 0.4/ 27.2 ± 0.5
8	>200	0.66	n.p	19.1 ± 1.8/ 29.5 ± 6.4	11.2 ± 1.1/ 125 ± 12	93 ± 29/ 8.0 ± 2.2
9	>200	0.87	n.p	12.2 ± 0.7/ 56.8 ± 3.1	50 ± 27/ 33 ± 14	29 ± 11/ 26 ± 10
10	>200	0.70	n.p	25.6 ± 3.0/ 27.3 ± 3.3	12.3 ± 2.1/ 114 ± 20	103 ± 22/ 6.9 ± 1.4

n.d. = not determined, n.p. = no permeability (compound not detected in basolateral compartment),

a) values are means of at least two independent experiments, and ± values represent standard deviation.

### 2.3 Investigation of Ternary Complex Formation via Native MS

**Figure 3:**
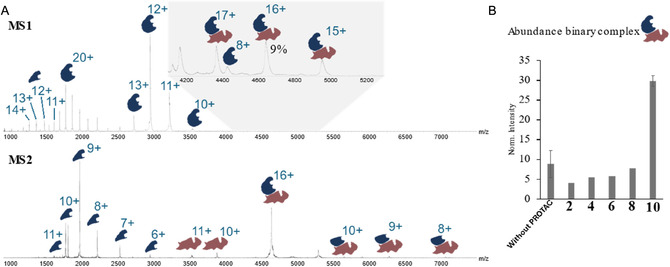
A) (top) nMS spectrum of LANA and CRBN with monomeric and dimeric LANA species present. Inset shows a zoomed‐in high‐*m/z* region showing charge states of the (LANA)_2_‐CRBN complex. (bottom) MS2 spectrum after isolation of the 16+ (LANA)_2_‐CRBN complex followed by mild collisional activation, resulting in ejection of LANA monomer and CRBN. B) Abundance of the binary LANA–CRBN complex in the presence of each CRBN‐based PROTAC candidate compared to the control without a PROTAC candidate present, normalized to monomeric LANA signals (charge states 11+ to 14+).

**Figure 4:**
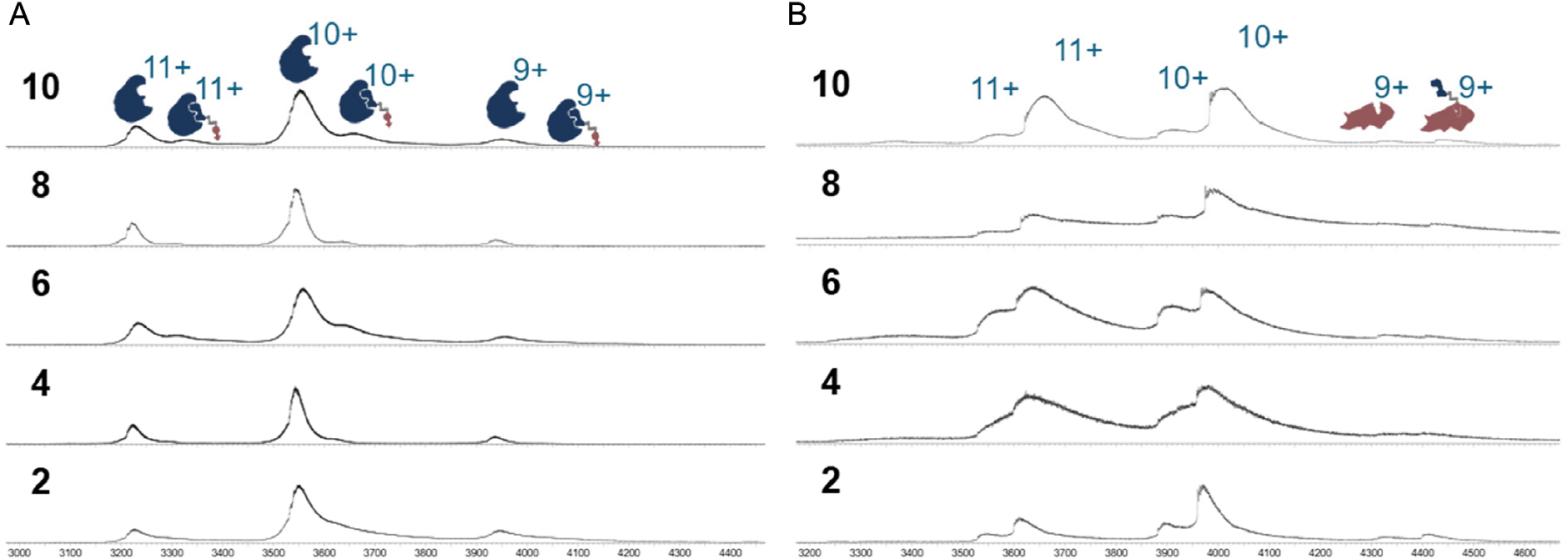
**A)** LANA‐binding capacity of each CRBN‐based PROTAC candidate validated by nMS. **B)** CRBN‐binding capacity of each CRBN‐based PROTAC candidate validated by nMS.

After confirming LANA binding, as a further step, we wanted to test our conjugates for their ability to induce formation of ternary complexes, which is an essential hallmark for PROTAC functionality. The generation of these higher‐order assemblies between ToI and the E3 ligase is necessary to enable poly‐ubiquitination and subsequent protein degradation. Therefore, we performed native mass spectrometry (MS) experiments to investigate whether or not the ternary complex (VHL‐compound‐LANA or CRBN‐compound‐LANA) was formed. We initially focused on the CRBN‐based PROTAC candidates, as CRBN has been the most commonly used E3 ubiquitin ligase for PROTAC discovery efforts, including compounds in clinical trials.^[^ ^13^ ^]^ For these experiments, we used a recently developed CRBN construct engineered for efficient expression and ease of handling.^[^ ^20^ ^]^ As a first experiment, we co‐incubated CRBN and LANA without addition of any of our PROTAC candidates (see **Figure** **3**A). Surprisingly, in addition to the expected species—monomeric and dimeric LANA as well as monomeric CRBN—we also observed signals for a complex between the LANA dimer and CRBN at around 10% of the intensity of the signals for monomeric LANA. As this result was quite unexpected, we isolated the 16+ charge state of this species and subjected it to mild collisional activation to dissociate it into subunits, which supported the identification of this ion as (LANA)_2_‐CRBN (see Figure 3A). While nonspecific adducts can form during ESI, this phenomenon typically occurs only at concentrations significantly higher than those (7–15 µM) we used in these experiments. As such, our results indicate that these two proteins are in fact able to form a complex in vitro, albeit with low affinity. While the LANA construct, which we used in this work, does contain a SOCS‐box motif (residues 1085–1100) and has been reported to form a complex with cullin 5 that subsequently mediates the ubiquitination and degradation of tumor suppressors VHL (via the N‐terminal domain, which is not part of the construct used in this work) and p53,^[^ ^21^ ^]^ to the best of our knowledge, a direct interaction with CRBN has so far not been reported. Next, we tested each of the five CRBN‐based PROTAC candidates (**2**, **4**, **6**, **8**, and **10**). While PROTAC‐induced ternary complexes have been detected with MS before,^[^ ^22^ ^–^ ^24^ ^]^ including in our own work,^[^ ^25^ ^–^ ^27^ ^]^ we were unable to directly detect signals corresponding to the ternary complex. In contrast, after incubating each protein separately with the PROTAC candidates, we were able to effectively detect the binary protein‐ligand complexes with native MS, indicating that binding in these complexes is not so labile as to not survive transfer into the gas phase (see **Figure** **4**). Intriguingly, while the relative intensity of the signals for the (LANA)_2_‐CRBN complex remained roughly the same or decreased slightly after the addition of compounds **2**, **4**, **6**, and **8**, co‐incubation of both proteins with compound **10** reproducibly resulted in a roughly threefold increase in the relative intensity of these signals (see Figure 3B). We hypothesize that compound **10** does in fact function as an inducer of the ternary complex and enhances the affinity between LANA and CRBN, but that binding of the compound is destabilized in the architecture of the resulting complex. This would explain why this interaction does not survive ionization but leaves behind a binary CRBN–LANA complex. It is known from literature^[^ ^23^ ^,^ ^26^ ^]^ that PROTAC‐induced complexes in the gas phase tend to dissociate by ejecting the PROTAC, as it is energetically favorable to preserve the protein–protein interaction surface in the gas phase at the expense of the protein‐ligand interactions. In contrast, we did not find any evidence for an interaction between LANA and VHL, either in the presence or absence of the VHL‐based PROTAC candidates (data not shown). As such, compound **10** seems to be the most efficient conjugate we generated in this study. For this PROTAC structure, we used a longer flexible PEG linker, which apparently helps to facilitate simultaneous binding to LANA and CRBN.

### 2.4 In Vitro ADME Properties

As a next step, we conducted in vitro absorption, distribution, metabolism, excretion (ADME) profiling. As we are using compounds beyond Lipinski's rule of five, we were interested in assessing the potential of these compounds for suitable pharmacokinetic properties. The compounds were tested for their kinetic solubility (1% DMSO in PBS pH 7.4), lipophilicity (chromatographic LogD_7.4_ value), cellular permeability (using Caco‐2 cells), and metabolic stability (using mouse and human liver S9 fractions and mouse liver microsomes) (see Table 2).

Despite native MS showing that most of the PROTAC candidates most likely cannot induce a ternary complex, we still decided to test all of them for their ADME profile to gain insights into relations between structure and solubility/stability/permeability to inform possible future work.

As summarized in Table 2, most of the compounds have at least moderate metabolic stability or even high stability in terms of hepatic turnover over a period of two hours. Especially compounds **1–4** turned out to be the most stable in mouse and human liver S9. The chromatographic LogD_7.4_ indicates similarly low lipophilicity for all compounds (LogD_7.4_ < 1). The compounds that have an aliphatic linker (compounds **1–4**) show a lower kinetic solubility around 50 µM. An increase in solubility was expected for conjugates with PEG‐linkers (Figure 2) and, indeed, this motif improved solubility, especially with longer chain length (compounds **6‐ 10**). However, PROTAC candidates containing PEG‐linkers were metabolically less stable compared to the other PROTAC candidates (**1–4**). Among all compounds tested, our most promising PROTAC candidate **10** (based on native MS results) showed an acceptable ADME profile, particularly in terms of solubility and stability in human liver S9. Mouse metabolic stability was lower compared to **1–4**, which will be taken into account during further optimization of the scaffold toward murine models. Another very important point for PROTACs is permeability, which we assessed using Caco‐2 cells. Cell permeability is a requirement. It is not just to enable absorption from the oral route (Lipinski Rule of five) but is essential for the intracellular bioavailability. Some PROTACs have been described to be able to fold themselves when having flexible linkers, thereby improving permeability.^[^ ^28^ ^]^ Although, the usage of flexible PEG‐linkers helped to overcome solubility issues, we were not able to detect any permeability across the Caco‐2 monolayer for any of these large molecules. As a consequence, we did not observe by Western Blot any targeted degradation of LANA in cells treated with these constructs (see Supporting Information, Figure S4, Supporting Information).

## 3 Conclusion

In this study, we synthesized PROTAC candidates able to bind to the viral KSHV LANA protein in vitro. Ten PROTAC candidates containing two different ToI‐binding motifs as well as two different E3 ligase ligands and different linkers were synthesized and characterized via different assays. The conjugates showed good binding affinity to the ToI. While no evidence supporting the formation of ternary complexes was found for VHL‐based PROTACs, compound **10** (CRBN‐based PROTAC) seems to be able to form this complex or at least enhance the affinity of CRBN for the LANA dimer. Especially by using longer flexible PEG‐linkers we obtained highly water‐soluble compounds. The longest of all PEG‐linkers used in this study also seems to be helpful for induction of ternary complex formation (compound **10**). A major limitation we noticed during this study is the low permeability of these large molecules as none of them gave satisfactory results in the Caco‐2 assay. With regards to the other ADME parameters, the PROTAC candidates have reasonable profiles in general. We aim to explore additional optimization strategies in the future, including further linker variation as there are different linkers described in literature to be highly efficient (flexible as well as sterically hindered ones).^[^ ^29^ ^]^ Each PROTAC system is very individual, and in addition to optimization of ToI‐ and E3‐ligase‐binding‐motifs, the linker regions require additional attention. Due to potentially conflicting structure–activity and structure‐property relationships (SAR vs SPRs) parallel optimization of essential parameters like solubility, permeability, metabolic stability, and of course binding affinity already at early discovery stages is very challenging to achieve a functional PROTAC against LANA as a hitherto unexplored viral target for targeted protein degradation. Other possible strategies to overcome the barriers we were confronted with are the use of smaller in‐cell click‐formed proteolysis targeting chimeras (CLIPTACs) and the use of nanoparticles as carriers. Thereby, one can bypass permeability issues and directly check whether the PROTACs are functional within the cellular context. Furthermore, additional parameters might impair functional induction of degradation such as lack of accessible lysine residues for ubiquitination, inadequate linker length or geometry, obstructing target protein orientation, as well as (viral) deubiquitinating enzymes.^[^ ^30^ ^,^ ^31^ ^]^ As the LANA protein is decorated with numerous basic amino acids on its surface for effective interaction with the phosphate backbone of double‐stranded DNA, lack of available lysines is unlikely. Investigating the other potential barriers for effective LANA degradation will be subject to future studies accompanied by expanded SAR studies including broad linkerology investigations.

In conclusion, we successfully synthesized bifunctional conjugates and created an assay pipeline to test PROTAC candidates step by step. This enables an assessment of the barriers encountered en route to functional and effective PROTACs and facilitates medicinal chemistry optimization of molecules not considered ‘drug‐like’ under traditional criteria.

## 4 Experimental Section

4.1

4.1.1

##### Chemistry

**Scheme 1:**
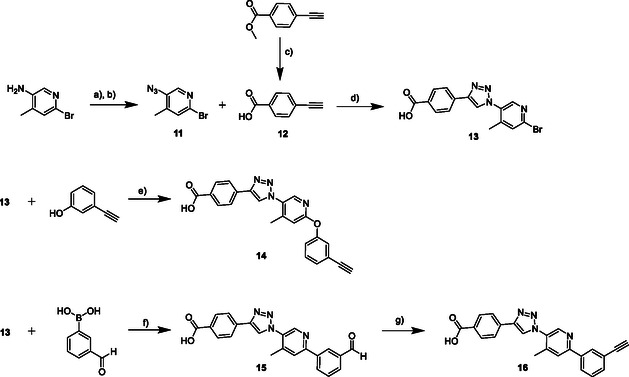
Synthesis of the precursors **14** and **16**. Reagents and conditions: a) 1.7 eq. NaNO_2_, 6M HCl, EtOAc, H_2_O, 0 °C, 30 min; b) 1.7 eq. NaN_3_, rt, 1 h; c) 1 M NaOH, THF/MeOH 1:1, rt, overnight; d) 2.0 eq. DIPEA, 0.5 eq. Na‐ascorbate, 0.5 eq. CuSO_4_·5H_2_O, MeOH/H_2_O 1:1, argon, rt, overnight; e) 3.0 eq. Cs_2_CO_3_, 0.1 eq. CuI, 0.1 eq. Co(acac)_2_, DMF, argon, microwave, 160 °C, 5 min; f) 2.0 eq. boronic acid, 3.0 eq. Na_2_CO_3_, 0,1 eq. Pd(PPh_3_)_4_, H_2_O/1,4‐dioxane 1:1, argon, 80 °C, 0.5‐24 h; g) 4.0 eq. Cs_2_CO_3_, 1.2 eq. Ohira–Bestmann reagent, MeOH, rt, 2 h.

Compounds **11**, **12**, and **13** were synthesized according to a previous publication.^[^ ^8^ ^]^ Compound **14** was synthesized in an Ullmann–Diaryl ether synthesis in the microwave by using compound **13**, 3‐ethynylphenol, Cs_2_CO_3_, CuI, and cobalt(II)‐acetylacetonate in dimethylformamide (DMF).^[^ ^32^ ^]^ To obtain compound **16,** we first synthesized compound **20** by using a Suzuki–Miyaura coupling with compound **13**, 3‐formylphenylboronic acid, Na_2_CO_3_, and tetrakis(triphenylphosphine)palladium(0) in water/1,4‐dioxane.^[^ ^8^ ^]^ Afterward, compound **15** was converted to compound **16** in an Ohira–Bestmann reaction by using Cs_2_CO_3_ and the Ohira–Bestmann reagent in methanol (see **Scheme** **1**).

**Scheme 2:**
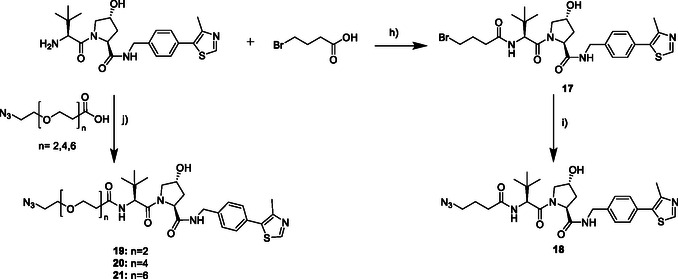
Synthesis of the VHL ligands **18‐21**. Reagents and conditions: h) 1.1 eq. HATU, 2.5 eq. DIPEA, DMF, 0 °C ‐> rt, overnight; i) 4.0 eq. NaN_3_, DMF, argon, 80 °C, overnight; j) 1.1 eq. HATU, 2.5 eq. DIPEA, DMF, 0 °C ‐> rt, 3h.

In **Scheme** **2**, the synthesis of the VHL‐ligands is shown. To obtain ligand **18**, we synthesized compound **17** in a first step via an amide coupling by using the VHL‐ligand, 4‐bromobutyric acid, O‐(7‐azabenzotriazol‐1‐yl)‐*N*,*N*,*N*′,*N*′‐tetramethyluronium‐hexafluorphosphate (HATU) and diisopropylethylamine (DIPEA) in DMF. In the second step, compound **17** was converted into compound **18** by using NaN_3_ in DMF. Ligands **19–21** were also synthesized in an amide coupling by using the VHL‐ligand, the respective carboxylic acid, HATU, and DIPEA in DMF.

**Scheme 3:**
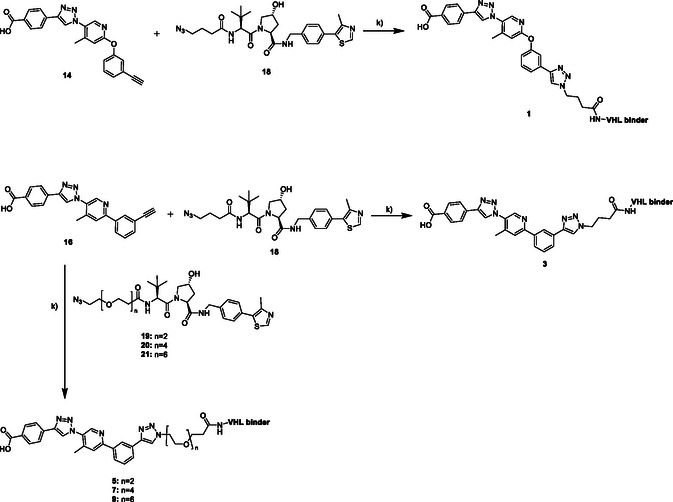
Synthesis of VHL‐binding PROTACs **1, 3, 5, 7,** and **9**. Reagents and conditions: k) 1.0 eq. CuSO_4_·5H_2_O, 2.0 eq. Na‐ascorbate, 2.0 eq. DIPEA, MeOH/H_2_O/DMF 1:1:1, argon, rt, overnight.

The synthesis of the VHL‐based PROTACs **1, 3, 5, 7,** and **9** is shown in **Scheme** **3**. For the synthesis of all PROTACs either compound **14** or compound **16** and the according VHL‐ligand were used in a copper‐catalyzed azide–alkyne cycloaddition (CuAAC) with CuSO_4_ · 5H_2_O, sodium ascorbate, and DIPEA in methanol/water/DMF.

**Scheme 4:**
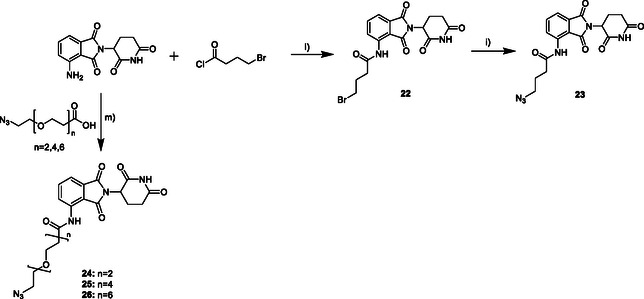
Synthesis of the CRBN ligands **23‐26**. Reagents and conditions: l) THF, argon, 60 °C, 4h; i) 2.0 eq. NaN_3_, DMF, argon, 80 °C, overnight; m) 3.0 eq. T3P, 2.5 eq. DIPEA, DMF, 80 °C, 6 h, rt, overnight.

**Scheme** **4** shows the synthesis of CRBN‐based ligands. For this, pomalidomide was used as educt reacting with 4‐bromobutyryl chloride in THF to compound **22**, which was then converted to compound **23** by using NaN_3_ in DMF. The synthesis of the ligands **24–26** is based on an amide coupling using pomalidomide, the respective carboxylic acid, propylphosphonic anhydride, and DIPEA in DMF.

**Scheme 5:**
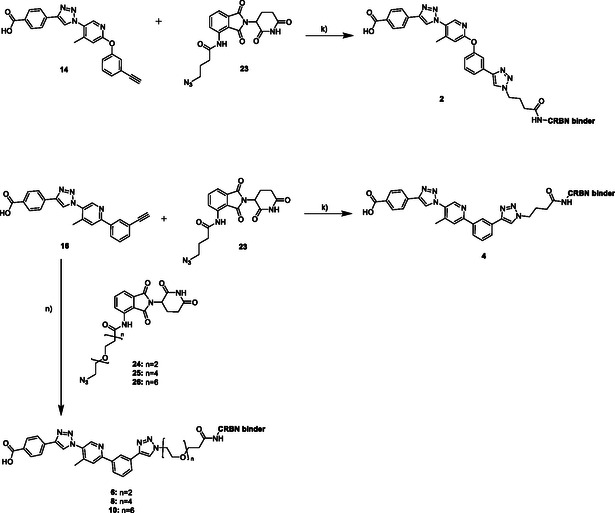
Synthesis of CRBN‐binding PROTACs **2, 4, 6, 8,** and **10**. Reagents and conditions: k) 1.0 eq. CuSO_4_·5H_2_O, 2.0 eq. Na‐ascorbate, 2.0 eq. DIPEA, MeOH/H_2_O/DMF 1:1:1, argon, rt, overnight; n) 0.5 eq. CuSO_4_·5H_2_O, 1.0 eq. Na‐ascorbate, 1.0 eq. DIPEA, 0.5 eq. THPTA, MeOH/H_2_O/DMF 1:1:1, argon, rt, overnight.

The synthesis of the CRBN‐based PROTACs **2, 4, 6, 8,** and **10** is shown in **Scheme** **5**. For the synthesis of PROTACs **2** and **4,** either compound **14** or compound **16** was used with compound **23** in a CuAAC reaction with CuSO_4_ · 5H_2_O, sodium ascorbate, and DIPEA in methanol/water/DMF. PROTACs **6, 8,** and **10** were synthesized by using compound **16**, the respective CRBN‐ligand, CuSO_4_ · 5H_2_O, sodium ascorbate, tris(3‐hydroxypropyltriazolylmethyl)amine and DIPEA in methanol/water/DMF.

Further synthesis, characterization, materials, and experimental details are provided in the Supporting Information.^[^ ^33^, ^34^ ^–^ ^35^ ^]^

## Conflict of Interest

The authors declare no conflict of interest.

## Supporting information

Supplementary Material

## Data Availability

The data that support the findings of this study are available from the corresponding author upon reasonable request.
