# Supplementary material for: Toward Anti‐Herpesviral PROTACs: Assessing the Challenges for Targeted Protein Degradation on the Example of Kaposi's Sarcoma‐Associated Herpesvirus Latency‐Associated Nuclear Antigen
Source: ChemMedChem. 2025 Nov 18;21(1):e202500758. doi: 10.1002/cmdc.202500758 (PMC12811992; doi:10.1002/cmdc.202500758)
Supplement: Supplementary file 1 — Supplementary Material [file CMDC-21-e202500758-s001.pdf]

Supporting Information

Towards Anti-Herpesviral PROTACs – Assessing The Challenges For Targeted Protein Degradation On The Example Of KSHV LANA

Aylin Berwanger[a,b,c,d], Saskia Catherina Stein[d,e,f], Sarah Brandner[g], Andreas Martin Kany[a,c], Sebastian Heinz[a,c], Brigitta Loretz[a,c], Claus-Michael Lehr[a,b,c], Anna Katharina Herta Hirsch[a,b,c,d], Frederik Lermyte[g], Thomas Friedrich Schulz[d,e,f], Martin Empting* [a,b,c,d,f]

This paper dedicated to Rolf Müller on the occasion of his 60th birthday.

[a] Aylin Berwanger, Dr. Andreas Martin Kany, Sebastian Heinz, Dr. Brigitta Loretz, Prof. Dr. Claus-Michael Lehr, Prof. Dr. Anna Katharina Herta Hirsch, Prof. Dr. Martin Empting
Helmholtz-Institute for Pharmaceutical Research Saarland (HIPS)
Campus E8.1, 66123 Saarbrücken, Germany
E-mail: aylin.berwanger@helmholtz-hips.de, andreas.kany@helmholtz-hips.de, sebastian.heinz@uni-saarland.de, brigitta.loretz@helmholtz-hips.de, claus-michael.lehr@helmholtz-hips.de, anna.hirsch@helmholtz-hips.de, martin.empting@helmholtz-hips.de

[b] Aylin Berwanger, Prof. Dr. Claus-Michael Lehr, Prof. Dr. Anna Katharina Herta Hirsch, Prof. Dr. Martin Empting
Department of Pharmacy
Saarland University
Campus E8.1, 66123 Saarbrücken, Germany
E-mail: aylin.berwanger@helmholtz-hips.de, claus-michael.lehr@helmholtz-hips.de, anna.hirsch@helmholtz-hips.de, martin.empting@helmholtz-hips.de

[c] Aylin Berwanger, Dr. Andreas Martin Kany, Sebastian Heinz, Dr. Brigitta Loretz, Prof. Dr. Claus-Michael Lehr, Prof. Dr. Anna Katharina Herta Hirsch, Prof. Dr. Martin Empting
PharmaScienceHub (PSH)
Saarland University
Campus E2.1, 66123 Saarbrücken, Germany
E-mail: aylin.berwanger@helmholtz-hips.de, andreas.kany@helmholtz-hips.de, sebastian.heinz@uni-saarland.de, brigitta.loretz@helmholtz-hips.de, claus-michael.lehr@helmholtz-hips.de, anna.hirsch@helmholtz-hips.de, martin.empting@helmholtz-hips.de

[d] Aylin Berwanger, Dr. Saskia Catherina Stein, Prof. Dr. Anna Katharina Herta Hirsch, Prof. Dr. Thomas Friedrich Schulz, Prof. Dr. Martin Empting
German Centre for Infection Research (DZIF)
Partner Site Hannover-Braunschweig
66123 Saarbrücken, Germany
E-mail: aylin.berwanger@helmholtz-hips.de, stein.saskia@mh-hannover.de, anna.hirsch@helmholtz-hips.de, schulz.thomas@mh-hannover.de, martin.empting@helmholtz-hips.de

[e] Dr. Saskia Catherina Stein, Prof. Dr. Thomas Friedrich Schulz
Institute of Virology
Hannover Medical School
Carl-Neuberg-Str. 1, 30625 Hannover, Germany
E-mail: stein.saskia@mh-hannover.de, schulz.thomas@mh-hannover.de

[f] Dr. Saskia Catherina Stein, Prof. Dr. Thomas Friedrich Schulz, Prof. Dr. Martin Empting
Cluster of Excellence RESIST (EXC 2155)
Hannover Medical School
Carl-Neuberg-Str. 1, 30625 Hannover, Germany
E-mail: stein.saskia@mh-hannover.de, schulz.thomas@mh-hannover.de, martin.empting@helmholtz-hips.de

[g] Sarah Brandner, Prof. Dr. Frederik Lermyte
Department of Chemistry
Technical University of Darmstadt
Peter-Grünberg-Str. 4, 64287 Darmstadt, Germany
E-mail: sarah.brandnr@tu-darmstadt.de, Frederik.lermyte@tu-darmstadt.de

Corresponding Author: Prof. Dr. Martin Empting (Martin.Empting@helmholtz-hips.de)

Table of Contents

1. Materials and Methods 4

1.1 Chemistry 4

1.2 Protein Expression 17

1.3 Microscale Thermophoresis (MST) assay 17

1.4 Electrophoretic Mobility Shift Assay (EMSA) 19

1.5 Kinetic Solubility 19

1.6 Chromatographic $LogD_{7.4}$ 19

1.7 Metabolic Stability 20

1.8 Cytotoxicity 20

1.9 Cell permeability 21

1.10 Native MS experiments 21

1.11 Cell-based assays 21

2. ^{1}H and ^{13}C Spectra 23

3. High Resolution Mass Spectra 37

4. HPLC Traces 44

5. References 49

1. Materials and Methods

1.1 Chemistry

The chemicals were purchased from commercial suppliers. Preparative high performance liquid chromatography (HPLC, Ultimate 3000 UHPLC+, Thermo Scientific) was used for the purification of the final compounds. Therefore, a reversed-phase column (VP 250/16 Nucleodur C18 Gravity, 5 µm, Macherey-Nagel, Germany) and water (containing 0.05% FA) and Acetonitril (containing 0.05% FA) as solvents were used. Reaction control was carried out by using TLC (TLC Silica Gel 60 F_{254} plates, Merck, Darmstadt, Germany) or by reversed-phase liquid chromatography mass spectrometry (LCMS, Thermo Scientific DIONEX UltiMate3000). The purity of the final compounds was determined by LCMS (Thermo Scientific DIONEX UltiMate3000, wavelength: 254 nm) and was >95% for all compounds. The ^{1}H- and ^{13}C-NMR spectra were recorded on a Bruker UltraShield 500 Plus nuclear magnetic resonance spectrometer at 499.90 MHz and 125.70 MHz, respectively. The spectra were evaluated with the software ACD/Spectrus Processor 2019.2.1. The signals were calibrated to the signal of the solvent DMSO-*d6*. The chemical shifts (δ) were given in parts per million (ppm) and the coupling constants (J) were given in hertz (Hz). High resolution masses (HRMS) were determined by LCMS/MS using a Thermo Scientific Q Exactive Focus (Germany) with a DIONEX ultimate 3000 UHPLC + focused.

General Procedure 1 (GP1): Amide coupling

a) 1.0 eq. of the amine and 1.0 eq. of the carboxylic acid were dissolved in DMF at 0°C. 1.1 eq. HATU and 2.5 eq. DIPEA were added and the mixture was stirred at room temperature 3-24 h. Then, H_2O was added and the mixture was extracted three times with DCM. The combined organic layers were dried over Na_2SO_4 and the solvent was removed under vacuum. The obtained crude product was used without further purification.

b) 1.0 eq. of the amine and 1.0 eq. of the carboxylic acid were dissolved in DMF. 3.0 eq. T3P (50% in EtOAc) and 2.5 eq. DIPEA were added. The mixture was stirred for 6h at 80°C and then overnight at room temperature. Then H_2O was added and the mixture was extracted with EtOAc three times. The combined organic layers were dried over Na_2SO_4 and the solvent was removed under vacuum. The obtained crude product was used without further purification.

General Procedure 2 (GP2): CuAAC

a) 1.2 eq. of the azide and 1.0 eq. of the alkyne were dissolved in MeOH/H_2O/DMF (1:1:1) and allowed to stir for ½ h at room temperature. Then a mixture of 1.0 eq. $CuSO_4 \cdot 5H_2O$, 2.0 eq. Na-ascorbate and 2.0 eq. DIPEA in H_2O previously prepared under argon at room temperature (½ h) was added. The mixture was stirred overnight at room temperature. 1 M HCl was added (pH~1) and the product was precipitated. To obtain the crude product, the solids were collected over vacuum filtration und dried under vacuum. The products were purified using preparative HPLC with the solvents water (containing 0.05% FA) and MeCN (containing 0.05% FA) (gradient elution, MeCN:H_2O 1.5:8.5 -> 0:10).

b) 1.2 eq. of the azide and 1.0 eq. of the alkyne were dissolved in MeOH/H_2O/DMF (1:1:1) and allowed to stir for ½ h at room temperature. Then a mixture of 0.5 eq. $CuSO_4 \cdot 5H_2O$, 0.5 eq. THPTA, 1.0 eq. Na-ascorbate and 1.0 eq. DIPEA in H_2O previously prepared under argon at room temperature (½ h) was added. The mixture was stirred overnight at room temperature. 1 M HCl was added (pH~1) and the product was precipitated. To obtain the crude product, the solids were collected over vacuum filtration und dried under vacuum. The products were purified using preparative HPLC with the solvents water (containing 0.05% FA) and MeCN (containing 0.05% FA) (gradient elution, MeCN:H_2O 1.5:8.5 -> 0:10).

Synthesis and characterization of 5-azido-2-bromo-4-methylpyridine (11)

The azide was synthesized as described previously.[1]

Synthesis and characterization of 4-ethynylbenzoic acid (12)

The acid was synthesized as described previously.[1]

Synthesis and characterization of 4-(1-(6-bromo-4-methylpyridin-3-yl)-1H-1,2,3-triazol-4-yl)benzoic acid (13)

The triazole was synthesized as described previously.[1]

Synthesis and characterization of 4-(1-(6-(3-ethynylphenoxy)-4-methylpyridin-3-yl)-1H-1,2,3-triazol-4-yl)benzoic acid (14)[2]

Compound **13** (1.0 eq., 30 mg, 0.08 mmol) was dissolved in DMF (2 mL) under argon. Cs_2CO_3 (3.0 eq., 82 mg, 0,24 mmol), CuI (0.1 eq., 1.5 mg, 0.008 mmol), $Co(acac)_2$ (0.1 eq., 2.1 mg, 0.008 mmol) and 3-ethynylphenol (3.0 eq., 27 µL, 0.24 mmol) were added. The mixture was stirred for 5 minutes at 160°C in the microwave. 1 M HCl was added (pH~1) and the product was precipitated. To obtain the crude product (99%, 0.08 mmol, 31 mg), the solids were collected over vacuum filtration und dried under vacuum. The product was purified using preparative HPLC with the solvents water (containing 0.05% FA) and MeCN (containing 0.05% FA) (gradient elution, MeCN:H_2O 1.5:8.5 -> 0:10).

HRMS (ESI): $[M+H]^+$ calculated: 397.12952 found: 397.12877

^{1}H-NMR (500 MHz, DMSO-*d6*, *δ* in ppm): 13.04 (br s, 1 H), 9.11 (s, 1 H), 8.33 (s, 1 H), 8.06 (s, 4 H), 7.47 (t, *J*=7.93 Hz, 1 H), 7.37 (d, *J*=7.78 Hz, 1 H), 7.30 - 7.34 (m, 1 H), 7.26 - 7.30 (m, 2 H), 4.28 (s, 1 H), 2.28 (s, 3 H).

^{13}C-NMR (126 MHz, DMSO-*d6*, *δ* in ppm): 167.1, 163.3, 153.5, 147.4, 146.0, 144.3, 134.4, 130.4, 130.3, 129.9, 128.6, 125.5, 124.8, 124.7, 123.3, 122.7, 113.1, 82.8, 81.8, 17.4.

Synthesis and characterization of 4-(1-(6-(3-formylphenyl)-4-methylpyridin-3-yl)-1H-1,2,3-triazol-4-yl)benzoic acid (15)[1]

Compound **13** (1.0 eq., 500 mg, 1.39 mmol) was dissolved in 10 mL H_2O/1,4-dioxane (1:1) under argon. Na_2CO_3 (3.0 eq., 442 mg, 4.17 mmol), $Pd(PPh_3)_4$ (0.1 eq., 161 mg, 0.14 mmol) and 3-formylphenylboronic acid (2.0 eq., 417 mg, 2.78 mmol) were added. The mixture was stirred overnight at 80°C. 1 M HCl was added (pH~1) and the product was precipitated. To obtain the crude product (97%, 1.35 mmol, 518 mg), the solids were collected over vacuum filtration und dried under vacuum. The product was purified using preparative HPLC with the solvents water (containing 0.05% FA) and MeCN (containing 0.05% FA) (gradient elution, MeCN:H_2O 1.5:8.5 -> 0:10).

HRMS (ESI): $[M+H]^+$ calculated: 385.12952 found: 385.12813

^{1}H-NMR (500 MHz, DMSO-*d6*, *δ* in ppm): 10.16 (s, 1 H), 9.25 (s, 1 H), 8.87 (s, 1 H), 8.74 (s, 1 H), 8.53 (d, *J*=7.93 Hz, 1 H), 8.34 (s, 1 H), 8.08 (s, 4 H), 8.03 - 8.07 (m, 1 H), 7.80 (t, *J*=7.71 Hz, 1 H), 2.44 (s, 3 H).

^{13}C-NMR (126 MHz, DMSO-*d6*, *δ* in ppm): 193.0, 166.9, 155.3, 145.8, 143.4, 138.2, 136.7, 133.9, 132.5, 132.4, 130.2, 130.0, 129.7, 127.8, 125.1, 124.2, 122.8, 17.3.

Synthesis and characterization of 4-(1-(6-(3-ethynylphenyl)-4-methylpyridin-3-yl)-1H-1,2,3-triazol-4-yl)benzoic acid 16)

Compound **15** (1.0 eq., 642 mg, 1.67 mmol) was dissolved in 20 mL MeOH. Cs_2CO_3 (4.0 eq., 2176 mg, 6.68 mmol) and the Ohira-Bestmann reagent (10% in MeCN, 1.2 eq, 300 µL, 2.00 mmol) were added. The reaction was stirred at room temperature for 2 h. 1 M HCl was added (pH~1) and the product was precipitated. To obtain the crude product (95%, 1.59 mmol, 605 mg), the solids were collected over vacuum filtration und dried under vacuum. The product was purified using preparative HPLC with the solvents water (containing 0.05% FA) and MeCN (containing 0.05% FA) (gradient elution, MeCN:H_2O 1.5:8.5 -> 0:10).

HRMS (ESI): $[M+H]^+$ calculated: 381.13460 found: 381.13325

^{1}H-NMR (500 MHz, DMSO-*d6*, *δ* in ppm): 9.23 (s, 1 H), 8.82 (s, 1 H), 8.28 (d, *J*=3.20 Hz, 2 H), 8.23 (br d, *J*=7.63 Hz, 1 H), 8.08 (s, 4 H), 7.61 (s, 1 H), 7.58 (d, *J*=7.63 Hz, 1 H), 4.30 (s, 1 H), 2.41 (s, 3 H).

^{13}C-NMR (126 MHz, DMSO-*d6*, *δ* in ppm): 167.4, 155.9, 146.3, 146.2, 143.8, 138.2, 134.5, 133.1, 132.9, 130.5, 130.3, 129.8, 127.7, 125.7, 124.7, 123.2, 122.8, 83.5, 81.6, 17.8.

Synthesis and characterization of (2S,4R)-1-((S)-2-(4-bromobutanamido)-3,3-dimethylbutanoyl)-4-hydroxy-N-(4-(4-methylthiazol-5-yl)benzyl)pyrrolidine-2-carboxamide (17)

Compound **17** was synthesized according to **GP1 a)** using 150 mg of the VHL-ligand (1.0 eq., 0.32 mmol), 10 mL DMF, 45 mg of 4-bromobutyric acid (1.0 eq., 0.32 mmol), 138 mg of HATU (1.1 eq., 0.35 mmol) and 141 µL of DIPEA (2.5 eq, 0.8 mmol). The obtained crude product was used without further purification (91%, 169 mg, 0.29 mmol).

LCMS (ESI): $[M+2H]^{2+}$ calculated: 581.57 found: 581.38

Synthesis and characterization of (2S,4R)-1-((S)-2-(4-azidobutanamido)-3,3-dimethylbutanoyl)-4-hydroxy-N-(4-(4-methylthiazol-5-yl)benzyl)pyrrolidine-2-carboxamide (18)

Compound **17** (1.0 eq., 562 mg, 0.97 mmol) was dissolved in 10 mL DMF under argon. NaN_3 (4.0 eq., 252 mg, 3.88 mmol) was added and the mixture was stirred overnight at 80°C. H_2O was added and the mixture was extracted three times with DCM. The combined organic layers were dried over Na_2SO_4 and the solvent was removed under vacuum to obtain the crude product (99%, 0.96 mmol, 520 mg).

LCMS (ESI): $[M+H]^+$ calculated: 542.68 found: 542.45

Synthesis and characterization of (2S,4R)-1-((S)-2-(3-(2-(2-azidoethoxy)ethoxy)propanamido)-3,3-dimethylbutanoyl)-4-hydroxy-N-(4-(4-methyl thiazol-5-yl)benzyl)pyrrolidine-2-carboxamide (19)

Compound **19** was synthesized according to **GP1 a)** using 70 mg of the VHL-ligand (1.0 eq., 0.15 mmol), 5 mL DMF, 30 mg of Azido-PEG2-acid (1.0 eq., 0.15 mmol), 63 mg of HATU (1.1 eq., 0.17 mmol) and 64 µL of DIPEA (2.5 eq, 0.38 mmol). The obtained crude product was used without further purification. The yield was determined after the next step.

HRMS (ESI): $[M+H]^+$ calculated: 616.29118 found: 616.28693

^{1}H-NMR (500 MHz, DMSO-*d6*, *δ* in ppm): 8.99 (s, 1 H), 8.61 (t, *J*=5.95 Hz, 1 H), 7.96 (d, *J*=9.31 Hz, 1 H), 7.42 (m, *J*=8.24 Hz, 2 H), 7.38 (m, *J*=8.09 Hz, 2 H), 5.16 (d, *J*=3.51 Hz, 1 H), 4.56 (d, *J*=9.46 Hz, 1 H), 4.43 (m, 2 H), 4.35 (br s, 1 H), 4.21 (dd, *J*=15.87, 5.34 Hz, 1 H), 3.56 (m, 10 H), 2.54 (m, 3 H), 2.44 (s, 3 H), 2.36 (m, 1 H), 2.02 (br d, *J*=7.93 Hz, 1 H), 1.90 (m, 1 H), 0.93 (s, 9 H).

^{13}C-NMR (126 MHz, DMSO-*d6*, *δ* in ppm): 172.0, 170.0, 169.5, 151.6, 147.8, 139.6, 131.2, 129.7, 128.7, 127.4, 69.6, 69.5, 69.3, 68.9, 67.0, 58.7, 56.4, 56.3, 50.0, 41.7, 40.4, 38.0, 35.6, 35.4, 26.4, 16.0.

Synthesis and characterization of (2S,4R)-1-((S)-1-azido-17-(tert-butyl)-15-oxo-3,6,9,12-tetraoxa-16-azaoctadecan-18-oyl)-4-hydroxy-N-(4-(4-methylthiazol-5-yl)benzyl)pyroli dine-2-carboxamide (20)

Compound **20** was synthesized according to **GP1 a)** using 70 mg of the VHL-ligand (1.0 eq., 0.15 mmol), 5 mL DMF, 44 mg of Azido-PEG4-acid (1.0 eq., 0.15 mmol), 63 mg of HATU (1.1 eq., 0.17 mmol) and 64 µL of DIPEA (2.5 eq, 0.38 mmol). The obtained crude product was used without further purification. The yield was determined after the next step.

LCMS (ESI): $[M+H]^+$ calculated: 704.86 found: 704.44

Synthesis and characterization of (2S,4R)-1-((S)-1-azido-23-(tert-butyl)-21-oxo-3,6,9,12,15,18-hexaoxa-22-azatetracosan-24-oyl)-4-hydroxy-N-(4-(4-methylthiazol-5-yl) benzyl)pyrrolidine-2-carboxamide (21)

Compound **21** was synthesized according to **GP1 a)** using 70 mg of the VHL-ligand (1.0 eq., 0.15 mmol), 5 mL DMF, 57 mg of Azido-PEG6-acid (1.0 eq., 0.15 mmol), 63 mg of HATU (1.1 eq., 0.17 mmol) and 64 µL of DIPEA (2.5 eq, 0.38 mmol). The obtained crude product was used without further purification. The yield was determined after the next step.

LCMS (ESI): $[M+H]^+$ calculated: 792.97 found: 792.51

Synthesis and characterization of 4-(1-(6-(3-(1-(4-(((S)-1-((2S,4R)-4-hydroxy-2-((4-(4-methylthiazol-5-yl)benzyl)carbamoyl)pyrolidin-1-yl)-3,3-dimethyl-1-oxobutan-2-yl) amino)-4-oxobutyl)-1H-1,2,3-triazol-4-yl)phenoxy)-4-methylpyridin-3-yl)-1H-1,2,3-triazol-4-yl)benzoic acid (1)

Compound **1** was synthesized according to **GP2 a)** using 17 mg (1.0 eq., 0.044 mmol) of compound **14**, 29 mg (1.2 eq., 0.053 mmol) of compound **18**, 11 mg (1.0 eq., 0.044 mmol) of $CuSO_4 \cdot 5H_2O$, 17 mg (2.0 eq., 0.088 mmol) of Na-ascorbate and 15 µL (2.0 eq., 0.088 mmol) of DIPEA. 93% (38 mg, 0.041 mmol) of the product were obtained.

HRMS (ESI): $[M+H]^+$ calculated: 938.37664 found: 938.37270

^{1}H-NMR (500 MHz, DMSO-*d6*, *δ* in ppm): 9.11 (s, 1 H), 8.97 (s, 1 H), 8.65 (s, 1 H), 8.55 (t, *J*=5.95 Hz, 1 H), 8.34 (s, 1 H), 8.05 (s, 4 H), 8.01 (d, *J*=9.31 Hz, 1 H), 7.75 (d, *J*=7.93 Hz, 1 H), 7.67 (s, 1 H), 7.53 (t, *J*=7.93 Hz, 1 H), 7.41 (m, *J*=7.93 Hz, 2 H), 7.37 (m, *J*=8.09 Hz, 2 H), 7.25 - 7.32 (m, 1 H), 7.17 (dd, *J*=8.09, 2.29 Hz, 1 H), 5.08 - 5.20 (m, 1 H), 4.54 (d, *J*=9.16 Hz, 1 H), 4.35 - 4.47 (m, 4 H), 4.21 (dd, *J*=15.79, 5.42 Hz, 1 H), 3.60 - 3.73 (m, 2 H), 2.43 (s, 3 H), 2.26 - 2.31 (m, 4 H), 2.18 - 2.25 (m, 1 H), 1.99 - 2.14 (m, 4 H), 1.86 - 1.94 (m, 1 H), 0.94 (s, 9 H).

^{13}C-NMR (126 MHz, DMSO-*d6*, *δ* in ppm): 171.8, 171.0, 169.5, 167.0, 163.4, 157.7, 153.9, 151.4, 147.6, 147.1, 145.7, 145.5, 144.1, 139.4, 134.1, 132.6, 131.1, 130.4, 130.0, 129.5, 128.8, 128.5, 127.3, 125.2, 124.5, 121.8, 121.6, 120.6, 117.8, 116.1, 112.8, 112.0, 68.8, 58.6, 56.5, 56.3, 49.1, 41.5, 37.8, 35.1, 31.4, 26.3, 25.9, 17.1, 15.8.

Synthesis and characterization of 4-(1-(6-(3-(1-(4-(((S)-1-((2S,4R)-4-hydroxy-2-((4-(4-methylthiazol-5-yl)benzyl)carbamoyl)pyrolidin-1-yl)-3,3-dimethyl-1-oxobutan-2-yl) amino)-4-oxobutyl)-1H-1,2,3-triazol-4-yl)phenyl)-4-methylpyridin-3-yl)-1H-1,2,3-triazol-4-yl)benzoic acid (3)

Compound **3** was synthesized according to **GP2 a)** using 42 mg (1.0 eq., 0.11 mmol) of compound **16**, 76 mg (1.2 eq., 0.14 mmol) of compound **18**, 27 mg (1.0 eq., 0.11 mmol) of $CuSO_4 \cdot 5H_2O$, 44 mg (2.0 eq., 0.22 mmol) of Na-ascorbate and 37 µL (2.0 eq., 0.22 mmol) of DIPEA. 99% (101 mg, 0.11 mmol) of the product were obtained.

HRMS (ESI): $[M+H]^+$ calculated: 922.38173 found: 922.38052

^{1}H-NMR (500 MHz, DMSO-*d6*, *δ* in ppm): 9.24 (s, 1 H), 8.96 (s, 1 H), 8.84 (s, 1 H), 8.73 (s, 1 H), 8.67 (s, 1 H), 8.55 (br t, *J*=5.95 Hz, 1 H), 8.25 - 8.28 (m, 1 H), 7.95 - 8.15 (m, 8 H), 7.62 (t, *J*=7.78 Hz, 2 H), 7.38 - 7.42 (m, 2 H), 7.33 - 7.38 (m, 2 H), 5.14 (br s, 1 H), 4.55 (d, *J*=9.31 Hz, 1 H), 4.43 (s, 3 H), 4.19 (s, 1 H), 3.67 (br s, 2 H), 2.42 (s, 6 H), 2.21 - 2.34 (m, 2 H), 2.01 - 2.16 (m, 3 H), 1.84 - 1.94 (m, 1 H), 0.95 (s, 9 H).

^{13}C-NMR (126 MHz, DMSO-*d6*, *δ* in ppm): 171.9, 171.1, 169.7, 167.0, 156.5, 151.5, 147.7, 146.1, 145.9, 145.8, 143.4, 139.5, 138.1, 134.3, 132.4, 131.6, 131.2, 130.3, 130.2, 129.6, 128.7, 128.6, 127.4, 126.3, 124.5, 123.6, 122.7, 121.8, 68.9, 58.7, 56.6, 56.4, 49.2, 41.6, 38.0, 35.2, 31.5, 26.4, 26.0, 17.5, 15.9.

Synthesis and characterization of 4-(1-(6-(3-(1-(2-(2-(3-(((S)-1-((2S,4R)-4-hydroxy-2-((4-(4-methylthiazol-5-yl)benzyl)carbamoyl)pyrolidin-1-yl)-3,3-dimethyl-1-oxobutan-2-yl) amino)-3-oxopropoxy)ethoxy)ethyl)-1H-1,2,3-triazol-4-yl)phenyl)-4-methylpyridin-3-yl)-1H-1,2,3-triazol-4-yl)benzoic acid (5)

Compound **5** was synthesized according to **GP2 a)** using 50 mg (1.0 eq., 0.13 mmol) of compound **16**, 96 mg (1.2 eq., 0.16 mmol) of compound **19**, 32 mg (1.0 eq., 0.13 mmol) of $CuSO_4{\cdot}5H_2O$, 52 mg (2.0 eq., 0.26 mmol) of Na-ascorbate and 44 µL (2.0 eq., 0.26 mmol) of DIPEA. 82% (106 mg, 0.11 mmol) of the product were obtained (over two steps).

HRMS (ESI): $[M+H]^+$ calculated: 996.41851 found: 996.41410

^{1}H-NMR (500 MHz, DMSO-*d6*, *δ* in ppm): 9.19 (s, 1 H), 8.97 (s, 1 H), 8.84 (s, 1 H), 8.70 (s, 1 H), 8.67 (s, 1 H), 8.57 (br t, *J*=6.03 Hz, 1 H), 8.27 (s, 1 H), 8.14 (br d, *J*=7.93 Hz, 1 H), 8.01 - 8.04 (m, 4 H), 7.98 (br d, *J*=7.63 Hz, 1 H), 7.92 (br d, *J*=9.31 Hz, 1 H), 7.63 (t, *J*=7.78 Hz, 1 H), 7.35 - 7.42 (m, 4 H), 4.50 - 4.61 (m, 3 H), 4.39 - 4.45 (m, 2 H), 4.34 (br s, 1 H), 4.21 (br dd, *J*=15.79, 5.42 Hz, 1 H), 3.89 (br t, *J*=5.11 Hz, 2 H), 3.44 - 3.67 (m, 9 H), 2.32 - 2.44 (m, 8 H), 1.97 - 2.08 (m, 1 H), 1.89 (ddd, *J*=12.82, 8.39, 4.58 Hz, 1 H), 0.89 - 0.93 (m, 9 H).

^{13}C-NMR (126 MHz, DMSO-*d6*, *δ* in ppm): 171.6, 169.6, 169.2, 156.1, 151.1, 147.4, 146.0, 145.6, 145.5, 143.0, 139.1, 137.8, 132.5, 132.1, 131.2, 130.8, 129.7, 129.3, 129.2, 128.3, 127.1, 126.1, 125.9, 124.7, 123.7, 123.2, 122.1, 69.2, 69.1, 68.5, 68.4, 66.6, 58.4, 56.1, 55.9, 49.4, 41.3, 37.6, 35.3, 35.0, 26.0, 17.1, 15.6.

Synthesis and characterization of 4-(1-(6-(3-(1-((S)-17-((2S,4R)-4-hydroxy-2-((4-(4-methylthiazol-5-yl)benzyl)carbamoyl)pyrolidine-1-carbonyl)-18,18-dimethyl-15-oxo-3,6, 9,12-tetraoxa-16-azanonadecyl)-1H-1,2,3-triazol-4-yl)phenyl)-4-methylpyridin-3-yl)-1H-1,2,3-triazol-4-yl)benzoic acid (7)

Compound **7** was synthesized according to **GP2 a)** using 50 mg (1.0 eq., 0.13 mmol) of compound **16**, 113 mg (1.2 eq., 0.16 mmol) of compound **20**, 32 mg (1.0 eq., 0.13 mmol) of $CuSO_4 \cdot 5H_2O$, 52 mg (2.0 eq., 0.26 mmol) of Na-ascorbate and 44 µL (2.0 eq., 0.26 mmol) of DIPEA. 62% (88 mg, 0.08 mmol) of the product were obtained (over two steps).

HRMS (ESI): $[M+H]^+$ calculated: 1084.47093 found: 1084.46582

^{1}H-NMR (500 MHz, DMSO-*d6*, *δ* in ppm): 9.24 (s, 1 H), 8.97 (s, 1 H), 8.84 (s, 1 H), 8.69 (s, 1 H), 8.67 (s, 1 H), 8.56 (t, *J*=6.03 Hz, 1 H), 8.27 (s, 1 H), 8.14 (br d, *J*=7.94 Hz, 1 H), 8.05 - 8.12 (m, 4 H), 7.98 (d, *J*=7.63 Hz, 1 H), 7.90 (d, *J*=9.31 Hz, 1 H), 7.63 (t, *J*=7.78 Hz, 1 H), 7.33 - 7.44 (m, 4 H), 4.61 (t, *J*=5.04 Hz, 2 H), 4.54 (d, *J*=9.46 Hz, 1 H), 4.38 - 4.45 (m, 2 H), 4.34 (br s, 1 H), 4.17 - 4.26 (m, 1 H), 3.90 (t, *J*=5.19 Hz, 2 H), 3.60 - 3.67 (m, 2 H), 3.36 - 3.57 (m, 16 H), 2.43 (d, *J*=2.75 Hz, 6 H), 2.32 (br d, *J*=14.65 Hz, 1 H), 1.98 - 2.07 (m, 1 H), 1.89 (s, 1 H), 0.91 (s, 9 H).

^{13}C-NMR (126 MHz, DMSO-*d6*, *δ* in ppm): 171.8, 169.7, 169.4, 166.9, 156.3, 151.3, 147.5, 145.8, 145.6, 143.2, 139.3, 137.9, 134.0, 132.3, 131.4, 131.0, 130.0, 129.5, 129.4, 128.5, 127.2, 126.2, 126.1, 125.2, 124.2, 123.3, 122.5, 122.0, 69.5, 69.2, 68.7, 68.5, 66.7, 58.5, 56.2, 56.1, 49.5, 41.5, 37.8, 35.5, 35.2, 26.2, 26.1, 17.3, 15.8.

Synthesis and characterization of 4-(1-(6-(3-(1-((S)-23-((2S,4R)-4-hydroxy-2-((4-(4-methylthiazol-5-yl)benzyl)carbamoyl)pyrolidine-1-carbonyl)-24,24-dimethyl-21-oxo-3,6, 9,12,15,18-hexaoxa-22-azapentacosyl)-1H-1,2,3-triazol-4-yl)phenyl)-4-methylpyridin-3-yl)-1H-1,2,3-triazol-4-yl)benzoic acid (9)

Compound **9** was synthesized according to **GP2 a)** using 50 mg (1.0 eq., 0.13 mmol) of compound **16**, 127 mg (1.2 eq., 0.16 mmol) of compound **21**, 32 mg (1.0 eq., 0.13 mmol) of $CuSO_4 \cdot 5H_2O$, 52 mg (2.0 eq., 0.26 mmol) of Na-ascorbate and 44 µL (2.0 eq., 0.26 mmol) of DIPEA. 35% (54 mg, 0.05 mmol) of the product were obtained (over two steps).

HRMS (ESI): $[M+H]^+$ calculated: 1172.52336 found: 1172.51886

^{1}H-NMR (500 MHz, DMSO-*d6*, *δ* in ppm): 9.23 (s, 1 H), 8.97 (s, 1 H), 8.84 (s, 1 H), 8.69 (s, 1 H), 8.67 (s, 1 H), 8.56 (t, *J*=6.03 Hz, 1 H), 8.27 (s, 1 H), 8.14 (d, *J*=7.93 Hz, 1 H), 8.05 - 8.11 (m, 4 H), 7.98 (d,

J=7.63 Hz, 1 H), 7.90 (d, *J*=9.46 Hz, 1 H), 7.63 (t, *J*=7.78 Hz, 1 H), 7.34 - 7.44 (m, 4 H), 4.60 (t, *J*=5.04 Hz, 2 H), 4.54 (d, *J*=9.46 Hz, 1 H), 4.39 - 4.45 (m, 2 H), 4.34 (br s, 1 H), 4.21 (dd, *J*=15.87, 5.49 Hz, 1 H), 3.90 (t, *J*=5.19 Hz, 2 H), 3.41 - 3.63 (m, 26 H), 2.43 (d, *J*=3.05 Hz, 6 H), 2.29 - 2.39 (m, 1 H), 2.01 (br d, *J*=7.63 Hz, 1 H), 1.87 - 1.93 (m, 1 H), 0.92 (s, 9 H).

^{13}C-NMR (126 MHz, DMSO-*d6*, *δ* in ppm): 171.9, 169.9, 169.5, 167.1, 156.5, 151.4, 147.7, 146.0, 145.9, 145.8, 143.4, 139.5, 138.1, 134.0, 132.4, 131.5, 131.2, 130.1, 129.6, 128.6, 127.4, 126.4, 126.3, 125.3, 124.4, 123.5, 122.7, 122.2, 69.7, 69.6, 69.4, 68.9, 68.6, 66.9, 58.7, 56.4, 56.3, 49.7, 41.6, 37.9, 35.6, 35.3, 26.3, 17.4, 15.9.

Synthesis and characterization of 4-bromo-N-(2-(2,6-dioxopiperidin-3-yl)-1,3-dioxoisoindolin-4-yl)butanamide (22)[3]

Pomalidomide (1.0 eq., 300 mg, 1.10 mmol) was dissolved in 5 mL THF. 4-Bromobutanoyl chloride (2.0 eq., 255 µL, 2.20 mmol) was added and the mixture was stirred for 4 h under argon at 60°C. Tthe solvent was removed under vacuum, then DCM was added and the solution was washed three times with sat. $NaHCO_3$. The organic layer was dried over Na_2SO_4 and the solvent was removed under vacuum to yield 99% (1.1 mmol, 464 mg) of the crude product.

LCMS (ESI): $[M+H]^+$ calculated: 422.24 found: 422.12

Synthesis and characterization of 4-azido-N-(2-(2,6-dioxopiperidin-3-yl)-1,3-dioxoisoindolin-4-yl)butanamide (23)[3]

Compound **22** (1.0 eq., 464 mg, 1.1 mmol) was dissolved in 4 mL DMF under argon. NaN_3 (2.0 eq., 143 mg, 2.20 mmol) was added and the mixture was stirred overnight at 80°C. H_2O was added and the mixture was extracted three times with EtOAc. The combined organic layers were dried over Na_2SO_4 and the solvent was removed under vacuum to obtain the crude product (99%, 1.1 mmol, 422 mg).

LCMS (ESI): $[M+H]^+$ calculated: 385.36 found: 385.11

Synthesis and characterization of 3-(2-(2-azidoethoxy)ethoxy)-N-(2-(2,6-dioxopiperidin-3-yl)-1,3-dioxoisoindolin-4-yl)propanamide (29)

Compound **24** was synthesized according to **GP1 b)** using 100 mg of Pomalidomide (1.0 eq., 0.37 mmol), 4 mL DMF, 75 mg of Azido-PEG2-acid (1.0 eq., 0.37 mmol), 660 µL of T3P (50% in MeCN,

3.0 eq., 1.11 mmol) and 157 µL of DIPEA (2.5 eq, 0.93 mmol). The obtained crude product was used without further purification (64%, 0.24 mmol, 110 mg).

LCMS (ESI): $[M+H]^+$ calculated: 459.44 found: 459.23

Synthesis and characterization of 1-azido-N-(2-(2,6-dioxopiperidin-3-yl)-1,3-dioxoisoindolin-4-yl)-3,6,9,12-tetraoxapentadecan-15-amide (25)

Compound **25** was synthesized according to **GP1 b)** using 100 mg of Pomalidomide (1.0 eq., 0.37 mmol), 4 mL DMF, 108 mg of Azido-PEG4-acid (1.0 eq., 0.37 mmol), 660 µL of T3P (50% in MeCN, 3.0 eq., 1.11 mmol) and 157 µL of DIPEA (2.5 eq, 0.93 mmol). The obtained crude product was used without further purification (76%, 0.28 mmol, 153 mg).

LCMS (ESI): $[M+H]^+$ calculated: 547.54 found: 547.32

Synthesis and characterization of 1-azido-N-(2-(2,6-dioxopiperidin-3-yl)-1,3-dioxoisoindolin-4-yl)-3,6,9,12,15,18-hexaoxahenicosan-21-amide (26)

Compound **26** was synthesized according to **GP1 b)** using 100 mg of Pomalidomide (1.0 eq., 0.37 mmol), 4 mL DMF, 140 mg of Azido-PEG6-acid (1.0 eq., 0.37 mmol), 660 µL of T3P (50% in MeCN, 3.0 eq., 1.11 mmol) and 157 µL of DIPEA (2.5 eq, 0.93 mmol). The obtained crude product was used without further purification (73%, 0.27 mmol, 171 mg).

LCMS (ESI): $[M+H]^+$ calculated: 635.65 found: 635.34

Synthesis and characterization of 4-(1-(6-(3-(1-(4-((2-(2,6-dioxopiperidin-3-yl)-1,3-dioxoisoindolin-4-yl)amino)-4-oxobutyl)-1H-1,2,3-triazol-4-yl)phenoxy)-4-methylpyridin-3-yl)-1H-1,2,3-triazol-4-yl)benzoic acid (2)

Compound **2** was synthesized according to **GP2 a)** using 55 mg (1.0 eq., 0.14 mmol) of compound **14**, 65 mg (1.2 eq., 0.17 mmol) of compound **23**, 35 mg (1.0 eq., 0.14 mmol) of $CuSO_4 \cdot 5H_2O$, 55 mg (2.0 eq., 0.28 mmol) of Na-ascorbate and 48 µL (2.0 eq., 0.28 mmol) of DIPEA. 99% (109 mg, 0.14 mmol) of the product were obtained.

HRMS (ESI): $[M+H]^+$ calculated: 781.24773 found: 781.24598

^{1}H-NMR (500 MHz, DMSO-*d6*, *δ* in ppm): 11.09 - 11.21 (m, 1 H), 9.76 - 9.83 (m, 1 H), 9.04 - 9.10 (m, 1 H), 8.66 - 8.70 (m, 1 H), 8.31 - 8.42 (m, 2 H), 7.96 - 8.10 (m, 4 H), 7.79 - 7.84 (m, 1 H), 7.72 - 7.76 (m, 1 H), 7.64 - 7.67 (m, 1 H), 7.60 - 7.63 (m, 1 H), 7.50 - 7.55 (m, 1 H), 7.26 - 7.30 (m, 1 H), 7.16 - 7.20 (m,

1 H), 5.09 - 5.18 (m, 1 H), 4.46 - 4.56 (m, 2 H), 2.52 - 2.65 (m, 4 H), 2.27 - 2.32 (m, 3 H), 2.19 - 2.24 (m, 2 H), 2.05 - 2.08 (m, 1 H), 1.21 - 1.27 (m, 2 H).

^{13}C-NMR (126 MHz, DMSO-*d6*, *δ* in ppm): 172.8, 171.0, 169.8, 167.4, 166.6, 163.4, 154.0, 147.2, 146.1, 145.7, 144.2, 136.2, 136.0, 133.3, 132.6, 131.5, 130.4, 129.7, 126.8, 125.1, 124.4, 123.5, 122.0, 121.7, 120.7, 118.2, 117.5, 116.2, 112.9, 112.8, 112.1, 49.0, 48.9, 33.0, 30.9, 25.3, 22.0, 17.2.

Synthesis and characterization of 4-(1-(6-(3-(1-(4-((2-(2,6-dioxopiperidin-3-yl)-1,3-dioxoisoindolin-4-yl)amino)-4-oxobutyl)-1H-1,2,3-triazol-4-yl)phenyl)-4-methylpyridin-3-yl)-1H-1,2,3-triazol-4-yl)benzoic acid (4)

Compound **4** was synthesized according to **GP2 a)** using 37 mg (1.0 eq., 0.10 mmol) of compound **16**, 45 mg (1.2 eq., 0.12 mmol) of compound **23**, 25 mg (1.0 eq., 0.10 mmol) of $CuSO_4 \cdot 5H_2O$, 40 mg (2.0 eq., 0.20 mmol) of Na-ascorbate and 34 µL (2.0 eq., 0.20 mmol) of DIPEA. 99% (76 mg, 0.10 mmol) of the product were obtained.

HRMS (ESI): $[M+H]^+$ calculated: 765.25282 found: 765.25283

^{1}H-NMR (500 MHz, DMSO-*d6*, *δ* in ppm): 11.13 (br s, 1 H), 9.80 (s, 1 H), 9.23 (s, 1 H), 8.84 (s, 1 H), 8.78 (s, 1 H), 8.65 (s, 1 H), 8.39 (d, *J*=8.39 Hz, 1 H), 8.27 (s, 1 H), 8.11 - 8.18 (m, 1 H), 8.08 (br s, 4 H), 7.97 (br d, *J*=7.63 Hz, 1 H), 7.81 (t, *J*=7.86 Hz, 1 H), 7.53 - 7.70 (m, 2 H), 5.13 (dd, *J*=12.82, 5.49 Hz, 1 H), 4.54 (br t, *J*=6.87 Hz, 2 H), 3.10 - 3.22 (m, 4 H), 2.51 - 2.62 (m, 3 H), 2.38 - 2.47 (m, 3 H), 2.18 - 2.36 (m, 2 H).

^{13}C-NMR (126 MHz, DMSO-*d6*, *δ* in ppm): 172.9, 171.2, 169.9, 167.6, 166.8, 160.0, 156.7, 146.3, 146.0, 143.5, 138.2, 136.4, 136.2, 132.6, 131.7, 130.0, 127.0, 126.7, 126.5, 126.4, 125.4, 124.5, 123.7, 122.8, 122.1, 118.7, 117.7, 57.7, 49.2, 49.1, 33.3, 31.1, 25.5, 23.2, 22.1, 19.4, 17.6, 13.7.

Synthesis and characterization of 4-(1-(6-(3-(1-(2-(2-(3-((2-(2,6-dioxopiperidin-3-yl)-1,3-dioxoisoindolin-4-yl)amino)-3-oxopropoxy)ethoxy)ethyl)-1H-1,2,3-triazol-4-yl)phenyl)-4-methylpyridin-3-yl)-1H-1,2,3-triazol-4-yl)benzoic acid (6)

Compound **6** was synthesized according to **GP2 b)** using 34 mg (1.0 eq., 0.09 mmol) of compound **16**, 50 mg (1.2 eq., 0.11 mmol) of compound **24**, 11 mg (0.5 eq., 0.05 mmol) of $CuSO_4 \cdot 5H_2O$, 18 mg (1.0 eq., 0.09 mmol) of Na-ascorbate, 27 mg (0.5 eq., 0.05 mmol) of THPTA and 15 µL (1.0 eq., 0.09 mmol) of DIPEA. 99% (75 mg, 0.09 mmol) of the product were obtained.

HRMS (ESI): $[M+H]^+$ calculated: 839.28960 found: 839.28963

^{1}H-NMR (500 MHz, DMSO-*d6*, *δ* in ppm): 11.16 (s, 1 H), 9.81 (s, 1 H), 9.19 - 9.25 (m, 1 H), 8.82 (s, 1 H), 8.61 - 8.68 (m, 1 H), 8.48 (d, *J*=8.39 Hz, 1 H), 8.24 (s, 1 H), 8.07 - 8.13 (m, 5 H), 7.95 (d, *J*=7.63 Hz, 1 H), 7.76 (t, *J*=7.93 Hz, 1 H), 7.53 - 7.62 (m, 2 H), 5.13 (dd, *J*=12.89, 5.42 Hz, 1 H), 4.54 - 4.61 (m, 2 H), 3.89 (t, *J*=5.04 Hz, 2 H), 3.70 (t, *J*=5.95 Hz, 2 H), 3.53 - 3.63 (m, 5 H), 2.84 - 2.93 (m, 1 H), 2.58 - 2.71 (m, 3 H), 2.38 - 2.44 (m, 3 H), 2.02 - 2.10 (m, 1 H).

^{13}C-NMR (126 MHz, DMSO-*d6*, *δ* in ppm): 173.1, 170.7, 170.2, 168.0, 167.0, 156.8, 146.3, 146.1, 143.6, 138.3, 136.7, 136.4, 132.7, 131.8, 131.7, 129.9, 126.6, 126.5, 126.2, 124.6, 123.8, 123.0, 122.5, 118.5, 116.8, 70.0, 69.8, 69.0, 66.4, 50.0, 49.2, 37.8, 31.2, 22.3, 17.8.

Synthesis and characterization of 4-(1-(6-(3-(1-(15-((2-(2,6-dioxopiperidin-3-yl)-1,3-dioxoisoindolin-4-yl)amino)-15-oxo-3,6,9,12-tetraoxapentadecyl)-1H-1,2,3-triazol-4-yl) phenyl)-4-methylpyridin-3-yl)-1H-1,2,3-triazol-4-yl)benzoic acid (8)

Compound **8** was synthesized according to **GP2 b)** using 34 mg (1.0 eq., 0.09 mmol) of compound **16**, 60 mg (1.2 eq., 0.11 mmol) of compound **25**, 11 mg (0.5 eq., 0.05 mmol) of $CuSO_4{\cdot}5H_2O$, 18 mg (1.0 eq., 0.09 mmol) of Na-ascorbate, 27 mg (0.5 eq., 0.05 mmol) of THPTA and 15 µL (1.0 eq., 0.09 mmol) of DIPEA. 74% (64 mg, 0.07 mmol) of the product were obtained.

HRMS (ESI): $[M+H]^+$ calculated: 927.34203 found: 927.34235

^{1}H-NMR (500 MHz, DMSO-*d6*, *δ* in ppm): 11.15 (s, 1 H), 9.83 (s, 1 H), 9.20 - 9.26 (m, 1 H), 8.80 - 8.87 (m, 1 H), 8.63 - 8.72 (m, 2 H), 8.52 (d, *J*=8.39 Hz, 1 H), 8.21 - 8.32 (m, 1 H), 8.07 - 8.18 (m, 4 H), 7.92 - 8.03 (m, 2 H), 7.80 (t, *J*=7.86 Hz, 1 H), 7.55 - 7.66 (m, 2 H), 5.13 (dd, *J*=12.89, 5.42 Hz, 1 H), 4.55 - 4.65 (m, 2 H), 3.83 - 3.95 (m, 3 H), 3.68 (t, *J*=5.95 Hz, 2 H), 3.40 - 3.58 (m, 11 H), 2.83 - 2.93 (m, 1 H), 2.56 - 2.71 (m, 3 H), 2.39 - 2.45 (m, 4 H), 2.02 - 2.10 (m, 1 H).

^{13}C-NMR (126 MHz, DMSO-*d6*, *δ* in ppm): 172.9, 170.5, 169.9, 167.8, 166.8, 156.6, 146.1, 145.9, 143.5, 138.3, 138.2, 136.6, 136.3, 134.3, 132.6, 131.7, 131.6, 129.7, 126.5, 126.4, 126.1, 124.5, 123.6, 122.8, 122.3, 118.4, 116.7, 69.9, 69.8, 69.7, 68.8, 66.2, 66.1, 51.4, 49.9, 49.1, 37.6, 34.5, 31.1, 22.1, 17.6.

Synthesis and characterization of 4-(1-(6-(3-(1-(21-((2-(2,6-dioxopiperidin-3-yl)-1,3-dioxoisoindolin-4-yl)amino)-21-oxo-3,6,9,12,15,18-hexaoxahenicosyl)-1H-1,2,3-triazol-4-yl)phenyl)-4-methylpyridin-3-yl)-1H-1,2,3-triazol-4-yl)benzoic acid (10)

Compound **10** was synthesized according to **GP2 b)** using 34 mg (1.0 eq., 0.09 mmol) of compound **16**, 70 mg (1.2 eq., 0.11 mmol) of compound **26**, 11 mg (0.5 eq., 0.05 mmol) of $CuSO_4{\cdot}5H_2O$, 18 mg (1.0 eq.,

0.09 mmol) of Na-ascorbate, 27 mg (0.5 eq., 0.05 mmol) of THPTA and 15 µL (1.0 eq., 0.09 mmol) of DIPEA. 99% (91 mg, 0.09 mmol) of the product were obtained.

HRMS (ESI): $[M+H]^+$ calculated: 1015.39446 found: 1015.39439

^{1}H-NMR (500 MHz, DMSO-*d6*, *δ* in ppm): 11.14 (s, 1 H), 9.85 (s, 1 H), 9.23 (s, 1 H), 8.84 (s, 1 H), 8.64 - 8.70 (m, 2 H), 8.53 (d, *J*=8.39 Hz, 1 H), 8.24 - 8.30 (m, 1 H), 8.05 - 8.17 (m, 5 H), 7.97 (d, *J*=7.63 Hz, 1 H), 7.81 (t, *J*=7.93 Hz, 1 H), 7.56 - 7.67 (m, 2 H), 5.13 (dd, *J*=12.89, 5.42 Hz, 1 H), 4.60 (t, *J*=5.04 Hz, 2 H), 3.90 (t, *J*=5.04 Hz, 2 H), 3.71 (t, *J*=5.95 Hz, 2 H), 3.37 - 3.60 (m, 21 H), 2.83 - 2.94 (m, 1 H), 2.68 (t, *J*=5.95 Hz, 2 H), 2.60 (br d, *J*=17.85 Hz, 1 H), 2.42 (s, 3 H), 2.01 - 2.11 (m, 1 H).

^{13}C-NMR (126 MHz, DMSO-*d6*, *δ* in ppm): 172.4, 170.1, 169.4, 167.3, 166.3, 156.2, 145.6, 145.4, 143.0, 137.7, 136.1, 135.8, 133.9, 131.2, 131.1, 129.2, 126.0, 125.9, 125.6, 124.1, 123.2, 122.3, 121.9, 117.9, 116.3, 69.4, 69.3, 69.2, 68.3, 65.8, 61.7, 49.4, 48.6, 37.2, 30.6, 25.1, 21.6, 17.1.

1.2 Protein Expression

The protein expression and purification of the His-tagged oligomerization-deficient mutant of the KSHV LANA *C*-terminal DNA binding domain (DBD; aa1008-1146), as well as for the CRBN construct and VHL was carried out according to previous publications.[4–6]

1.3 Microscale Thermophoresis (MST) assay

Microscale Thermophoresis assay was done as mentioned in a previous publication.[1,6]

1

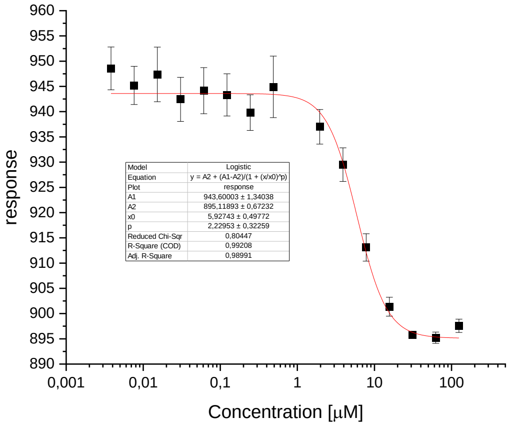

3

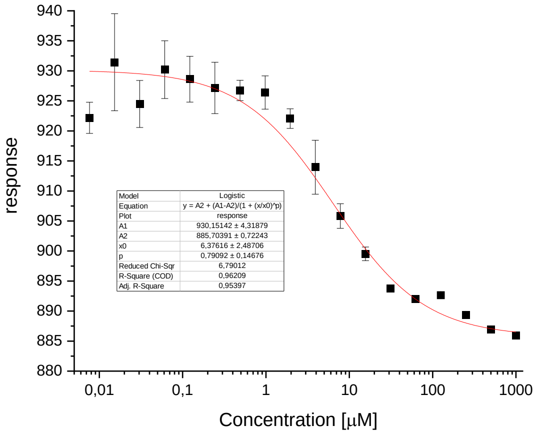

5

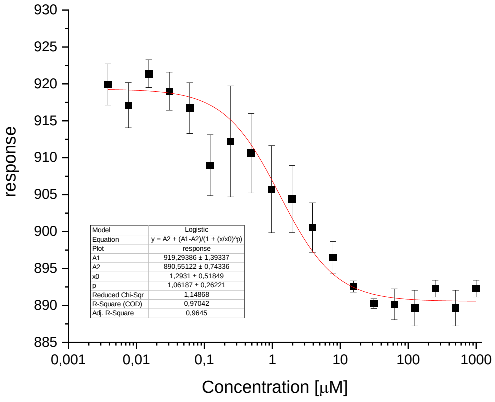

7

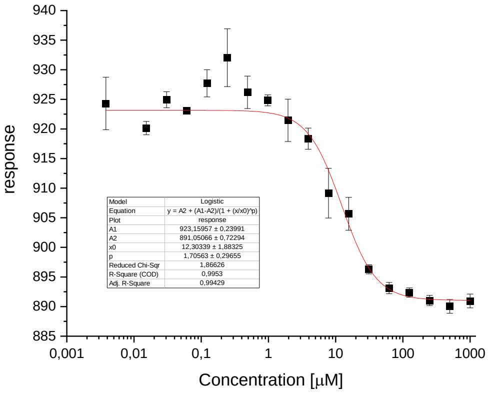

9

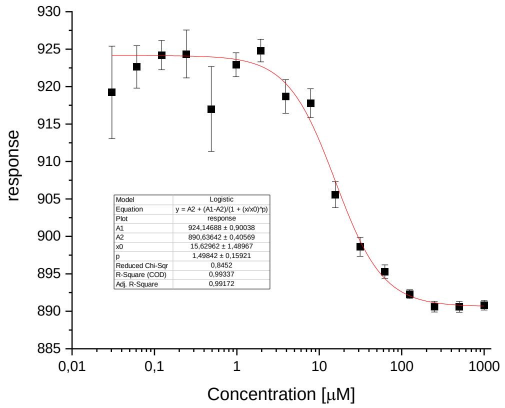

2

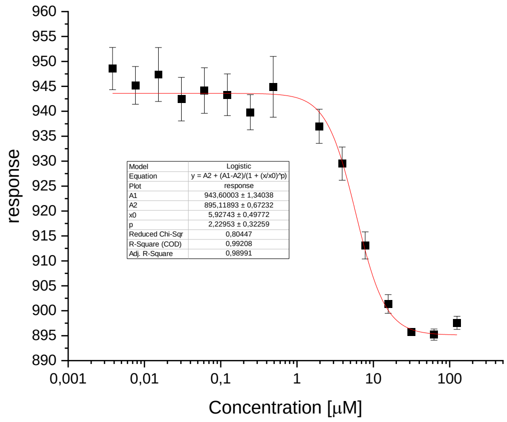

4

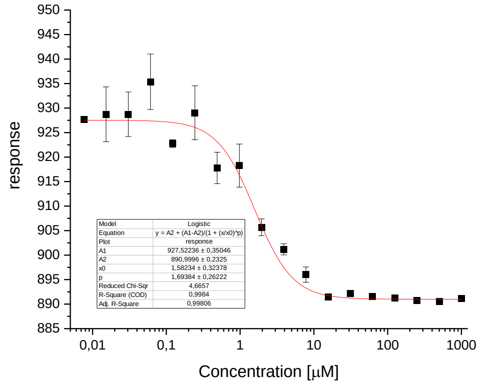

6

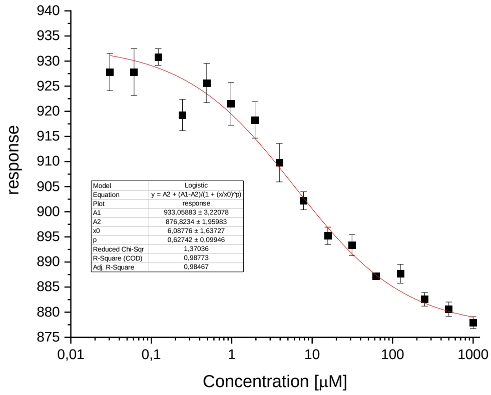

8

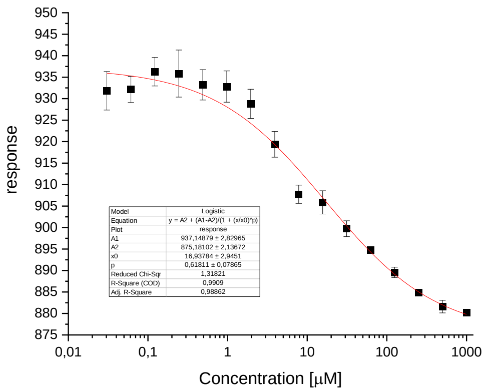

10

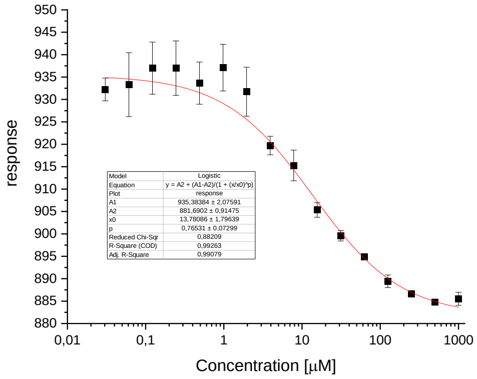

Figure S1. Binding affinity curves via MST for PROTACs **1-10**. Values are means of at least three replicates.

1.4 Electrophoretic Mobility Shift Assay (EMSA)

The EMSA was carried out with slight modifications as described previously (using 50 µM final concentration of the compounds)[1,6] based on the assay in Hellert et al. (2013).[7]

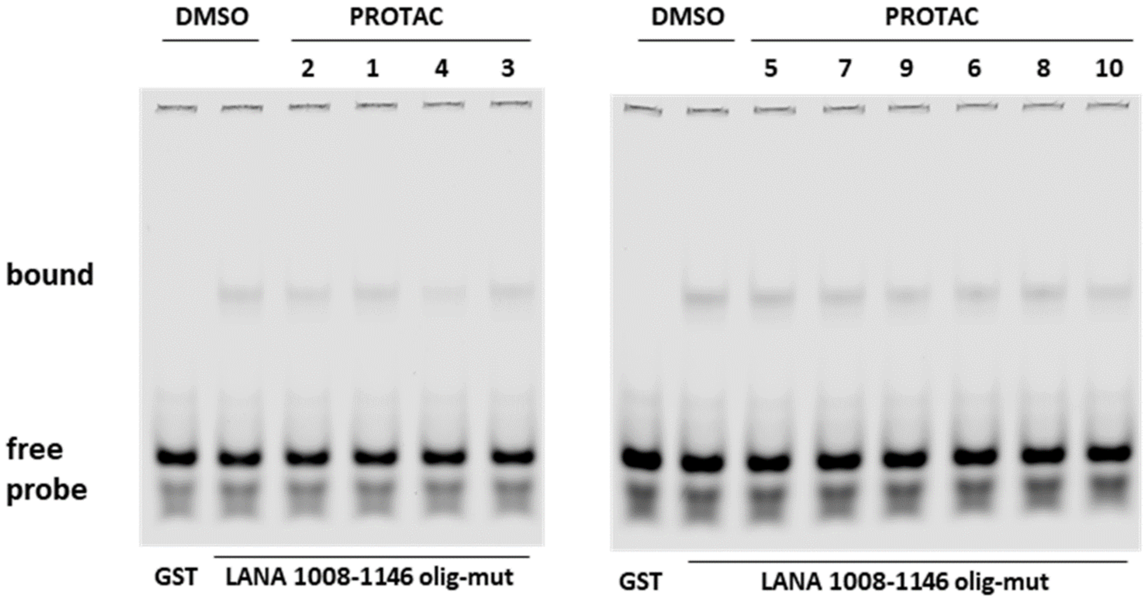

Figure S2. Gels of EMSA experiments at 50 µM for all PROTACs using LANA DBD mutant as well as LBS1.

1.5 Kinetic Solubility

The assay was performed as described previously.[1]

1.6 Chromatographic LogD$_{7.4}$

LogD$_{7.4}$ was analyzed using an HPLC−based method. The UV retention time of reference compounds with known LogD$_{7.4}$ was determined and plotted toward their LogD$_{7.4}$. Linear regression was used to determine the LogD$_{7.4}$ of unknown compounds. Analysis was performed using a Vanquish Flex HPLC system with variable wavelength detector (Thermo Fisher, Dreieich, Germany) with the following conditions: EC150/2 NUCLEODUR C18 Pyramid column, 5 µM (Macherey Nagel, Düren, Germany); eluent A: 50 mM NH_4OAc pH 7.4, eluent B: acetonitrile, and flow: 0.6 mL/min. The gradient was set to 0−100% B from 0 to 2.5 min, 100% B from 2.5 to 3.0 min, 100−0% B from 3.0 to 3.2 min, and 0% B from 3.2−5.0.

1.7 Metabolic Stability

The metabolic stability assay in mouse and human liver S9 fractions was performed as described previously.[1]

For the evaluation of phase I metabolic stability, the compound (1 µM) was incubated with 0.5 mg/mL pooled mouse liver microsomes (Xenotech, Kansas City, USA), 2 mM NADPH, 10 mM $MgCl_2$ at 37 °C for 120 min on a microplate shaker (Eppendorf, Hamburg, Germany). The metabolic stability of testosterone, verapamil and ketoconazole were determined in parallel to confirm the enzymatic activity of mouse liver microsomes. The incubation was stopped after defined time points by precipitation of aliquots of enzymes with 2 volumes of cold acetonitrile containing internal standard (15 nM diphenhydramine). Samples were stored on ice until the end of the incubation and precipitated protein was removed by centrifugation (15 min, 4 °C, 4,000 g). The remaining test compound at the different time points was analyzed by HPLC-MS/MS (Vanquish Flex coupled to a TSQ Altis Plus, Thermo Fisher, Dreieich, Germany) and used to determine half-life ($t_{1/2}$).

1.8 Cytotoxicity

The MTT assay in HEK293 cells was performed as described previously.[1]

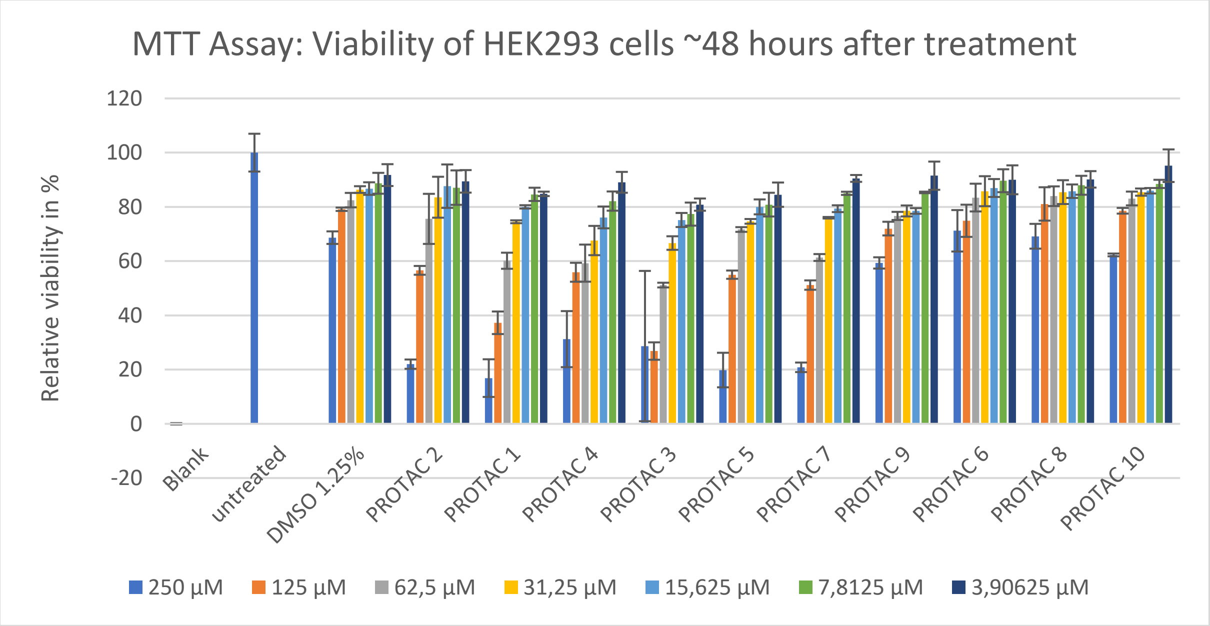

Figure S3. MTT assay for PROTACs **1-10** using HEK293 cells. Values are means of three replicates.

1.9 Cell permeability

The cell permeability assay was performed as described previously at a concentration of 10 µM.[1]

1.10 Native MS experiments

The native MS experiments were carried out using a Synapt XS instrument (Waters, Wilmslow, UK). Samples were desalted through buffer exchange into 200 mM ammonium acetate, performed using Zeba spin desalting columns (Thermo Fisher Scientific, Waltham, MA, USA) or Amicon centrifugal filters (Merck, Darmstadt, Germany). Each compound was mixed with the LANA protein or the E3 ligase complex at equimolar concentration (7-15 µM), incubated for at least 2 hours, and then introduced into the gas phase via nano-electrospray ionization (nanoESI) in positive-ion mode via glass emitters, fabricated in-house using a Sutter P-97 Flaming/Brown micropipette puller.

1.11 Cell-based assays

PROTACs (20 mM stocks in DMSO) were serially diluted in DMSO in three 1:2 steps to 10, 5 and 2.5 mM. BCBL-1 cells, a primary effusion lymphoma B-cell line latently infected with KSHV, were plated á $4x10^5$ cells per well in 1 mL medium (RPMI 1640, 20% FCS) in 24-well plates and treated with 1.25 µL of the diluted PROTACs or DMSO. The cells were incubated for 28 h at 37°C, 5% CO_2 in a humidified incubator. To harvest the cells they were pelleted and washed once with ice-cold phosphate-buffered saline (PBS). The cell pellet was resuspended in 50 µL modified RIPA buffer (20 mM Tris pH 7.6, 150 mM NaCl, 1mM EDTA, 1% NP40, 0.1% SDS) with freshly added Protease and Phosphatase inhibitors (1.5 µM Aprotinin, 1.46 µM Pepstatin A, 1 µM Benzamidine, 10 µM Leupeptin, 1 mM Na_3VO_4, 1 mM NaF, 100 µM PMSF), and lysed for 30 minutes at 4°C with rotation. Debris was pelleted 20 minutes at 4°C, 18,000 x g. 10 µL of the cleared supernatant was loaded per lane on 8% SDS acrylamide gels and separated by electrophoresis. Precision Plus Protein™ All Blue Prestained Protein Standards (Biorad, #1610373) was used as a size marker. Proteins were blotted to nitrocellulose membranes (Amersham, #10600003, Protran Premium 0.45 µM NC). Membranes were blocked with 5% milk in PBS with 0.01% Tween 20 for 1 hour and incubated overnight at 4°C with the indicated primary antibodies (α-LANA: Advanced Biotechnologies #13-210-100, Rat Anti-KSHV/HHV-8 ORF73 (LNA-1) monoclonal antibody, clone LN53; α-β-Actin: Sigma-Aldrich #A5441, mouse monoclonal antibody, clone AC-15)

diluted 1:1000 in the same buffer that was used for blocking. After washing the blots were incubated for one hour at room temperature with secondary antibodies (α-mouse: Dako, #P0260, polyclonal rabbit anti-mouse IgG HRP; α-rat: Southern Biotech, #3050-05, polyclonal goat anti-rat IgG (H+L) mouse-adsorbed HRP) diluted 1:1000 in the buffer used for blocking. After washing the blots were treated with SuperSignal West Femto Maximum Sensitivity Substrate (ThermoFisher, #34096) and the chemiluminescence imaged with the BioRad Chemidoc imager.

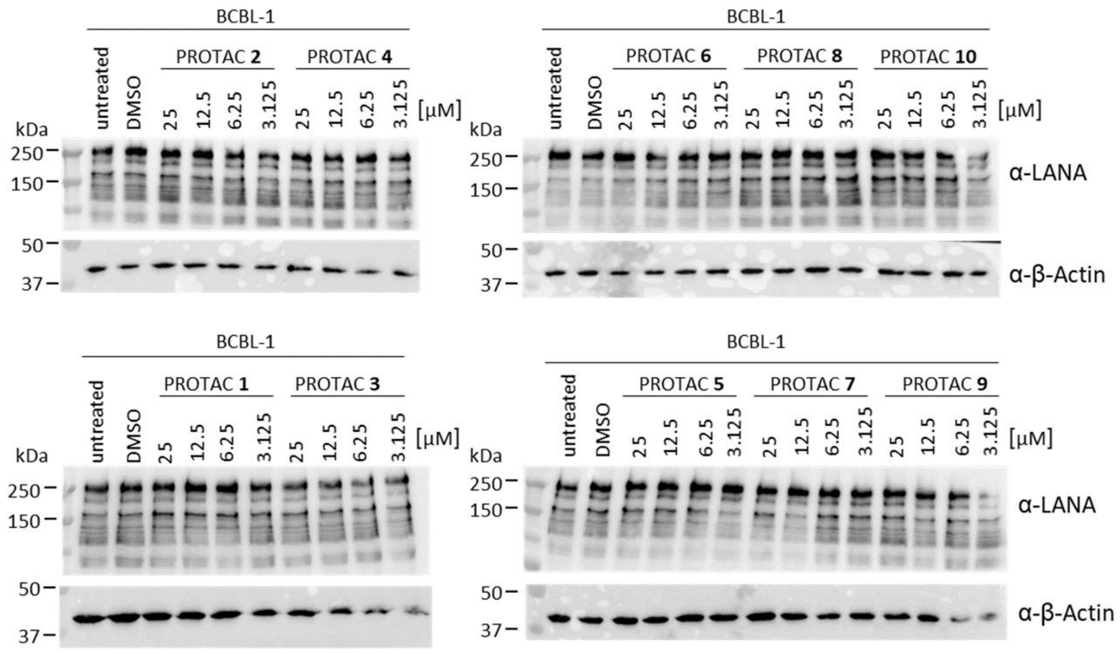

Figure S4. Western blots of PROTACs **1-10** after 28 hours of treatment.

2. ^{1}H and [13]C Spectra

4-(1-(6-(3-ethynylphenoxy)-4-methylpyridin-3-yl)-1H-1,2,3-triazol-4-yl)benzoic acid (**14**)

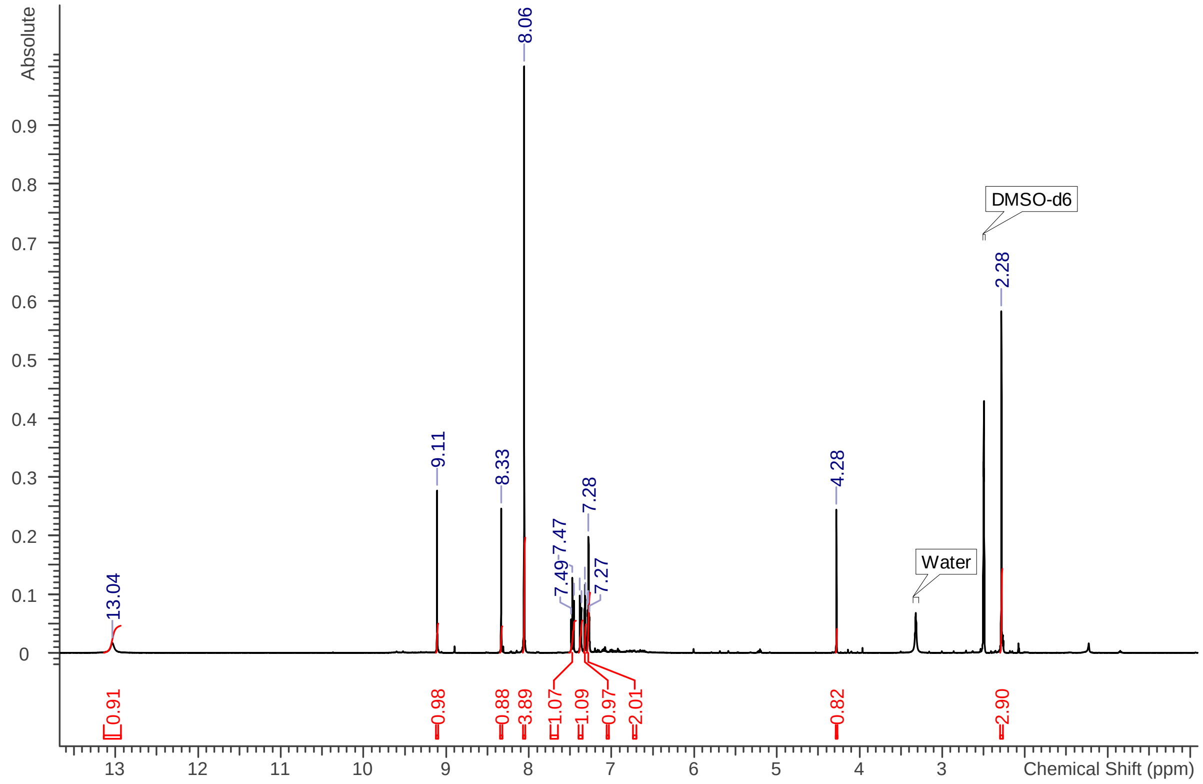

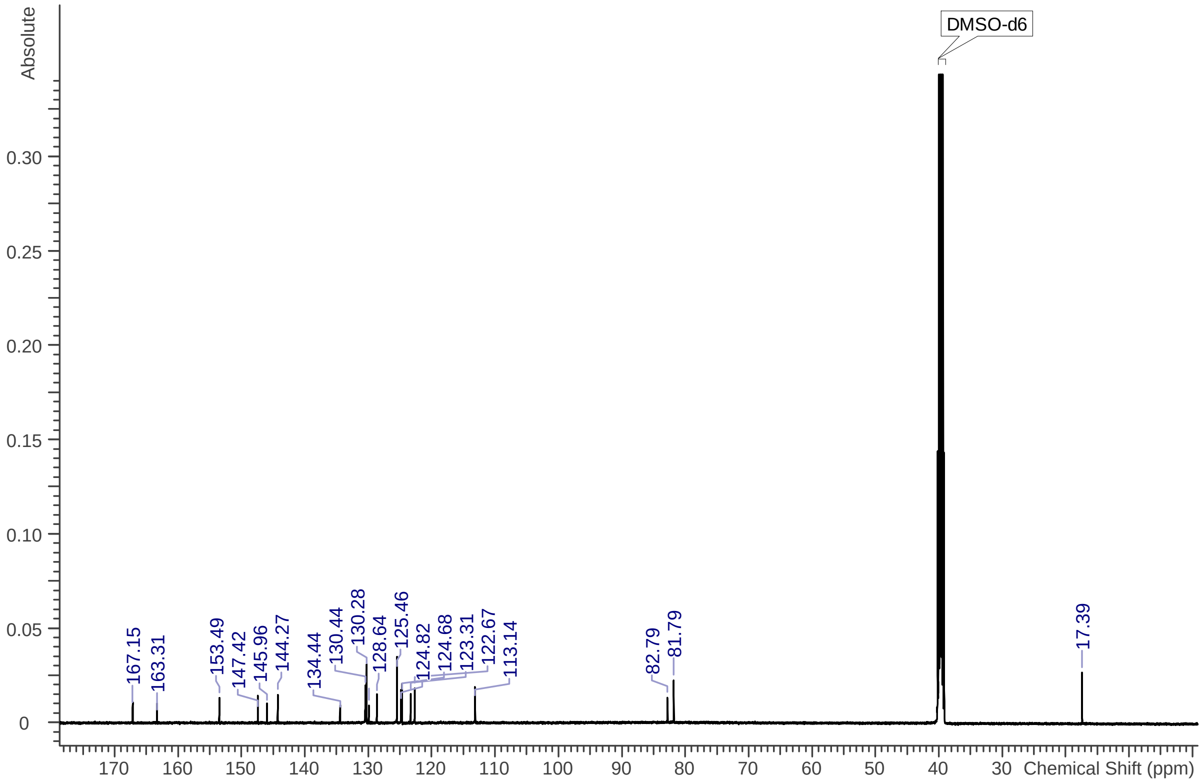

4-(1-(6-(3-formylphenyl)-4-methylpyridin-3-yl)-1H-1,2,3-triazol-4-yl)benzoic acid (**15**)

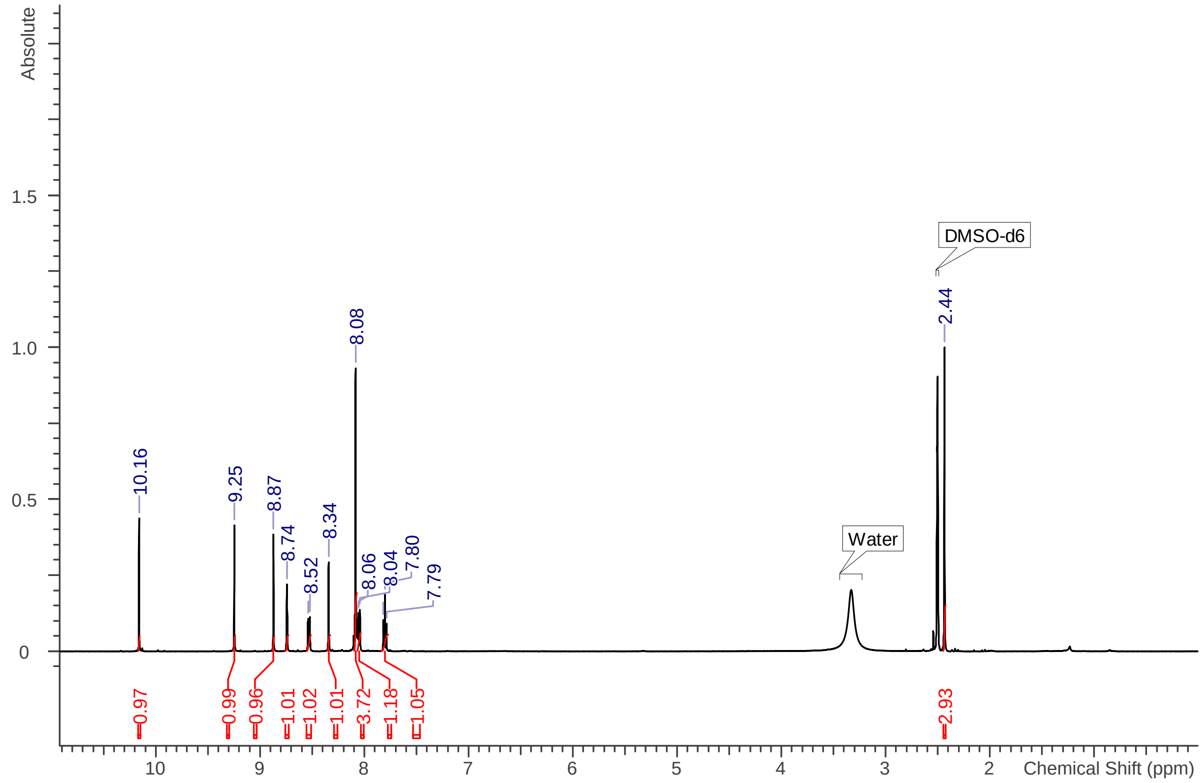

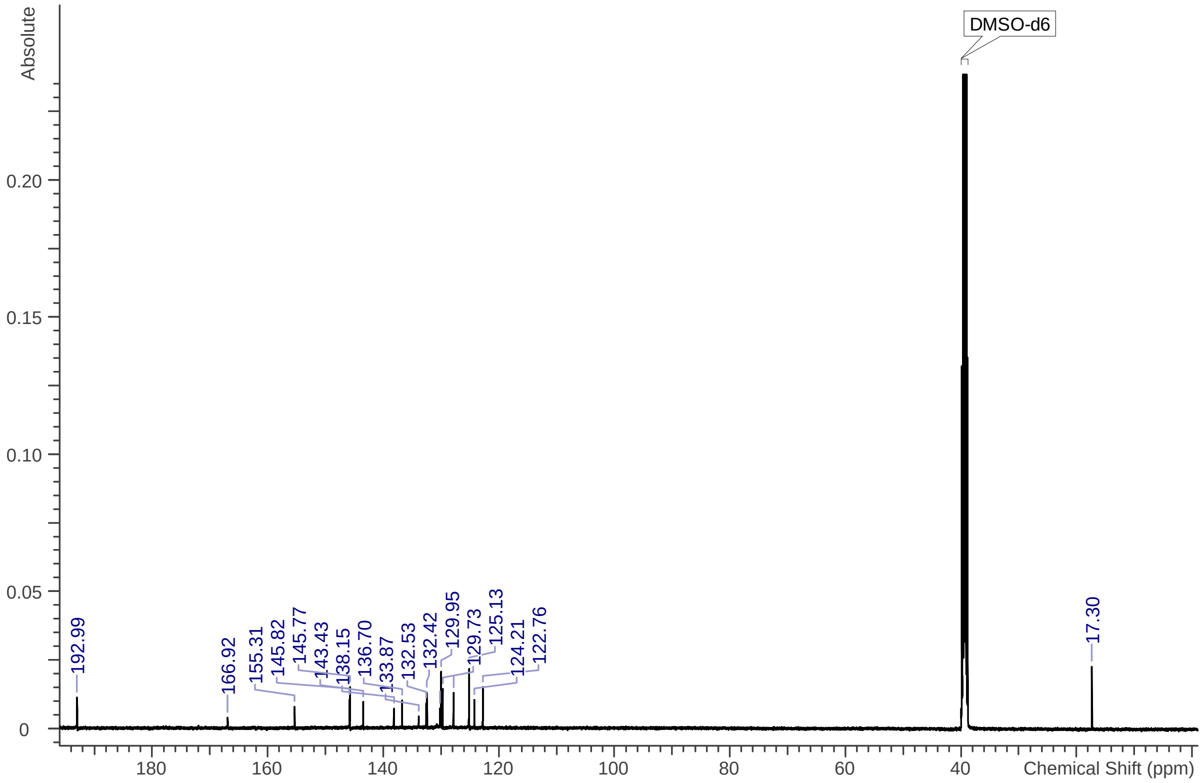

4-(1-(6-(3-ethynylphenyl)-4-methylpyridin-3-yl)-1H-1,2,3-triazol-4-yl)benzoic acid (**16**)

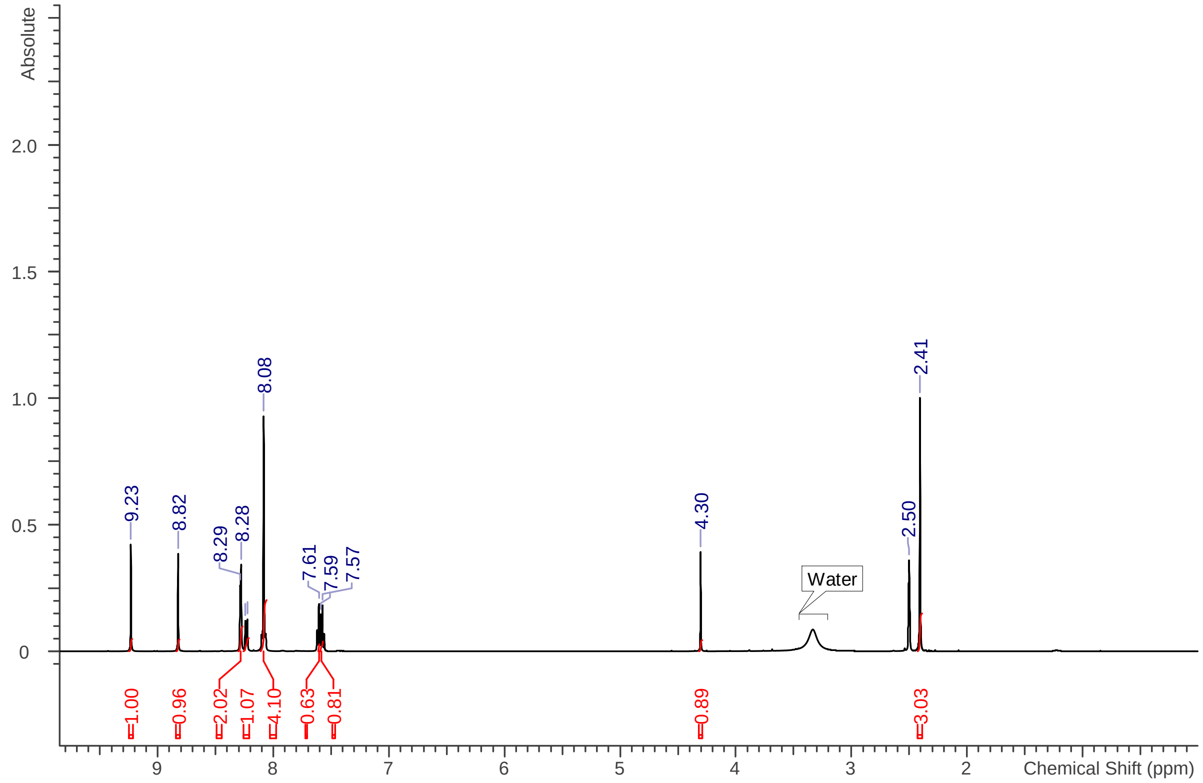

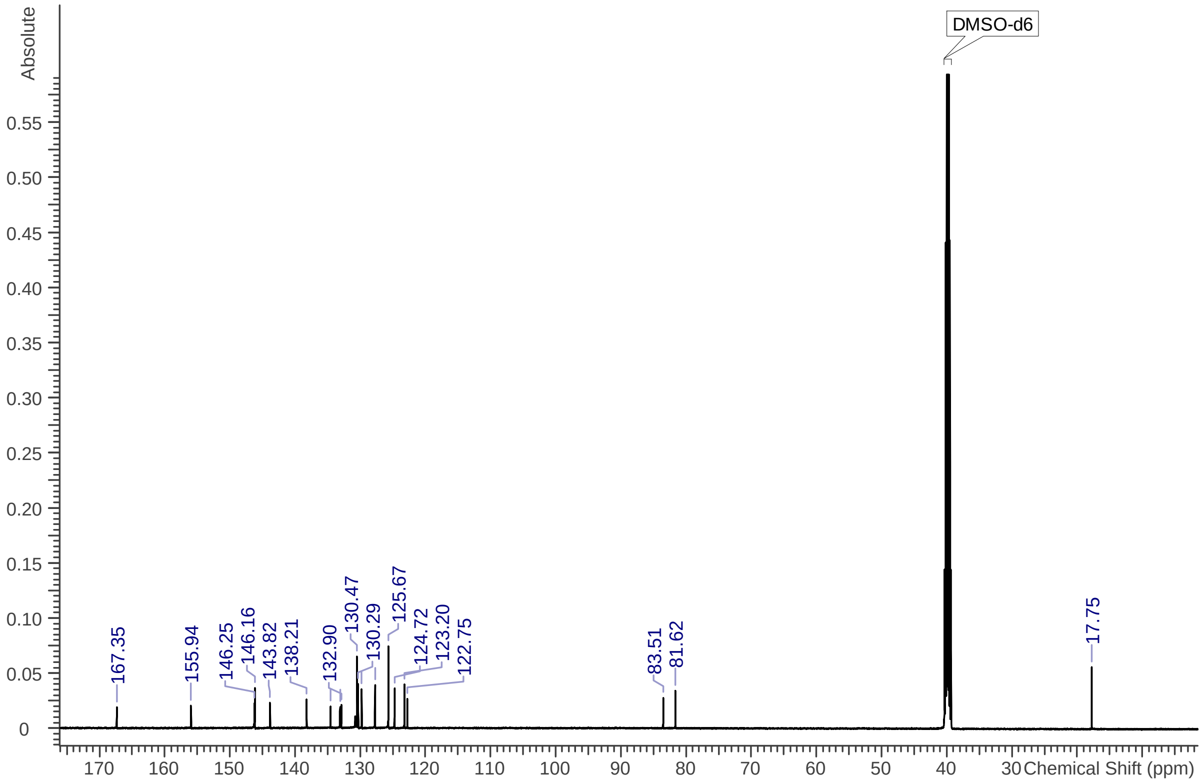

(2S,4R)-1-((S)-2-(3-(2-(2-azidoethoxy)ethoxy)propanamido)-3,3-dimethylbutanoyl)-4-hydroxy-N-(4-(4-methyl thiazol-5-yl)benzyl)pyrrolidine-2-carboxamide (**19**)

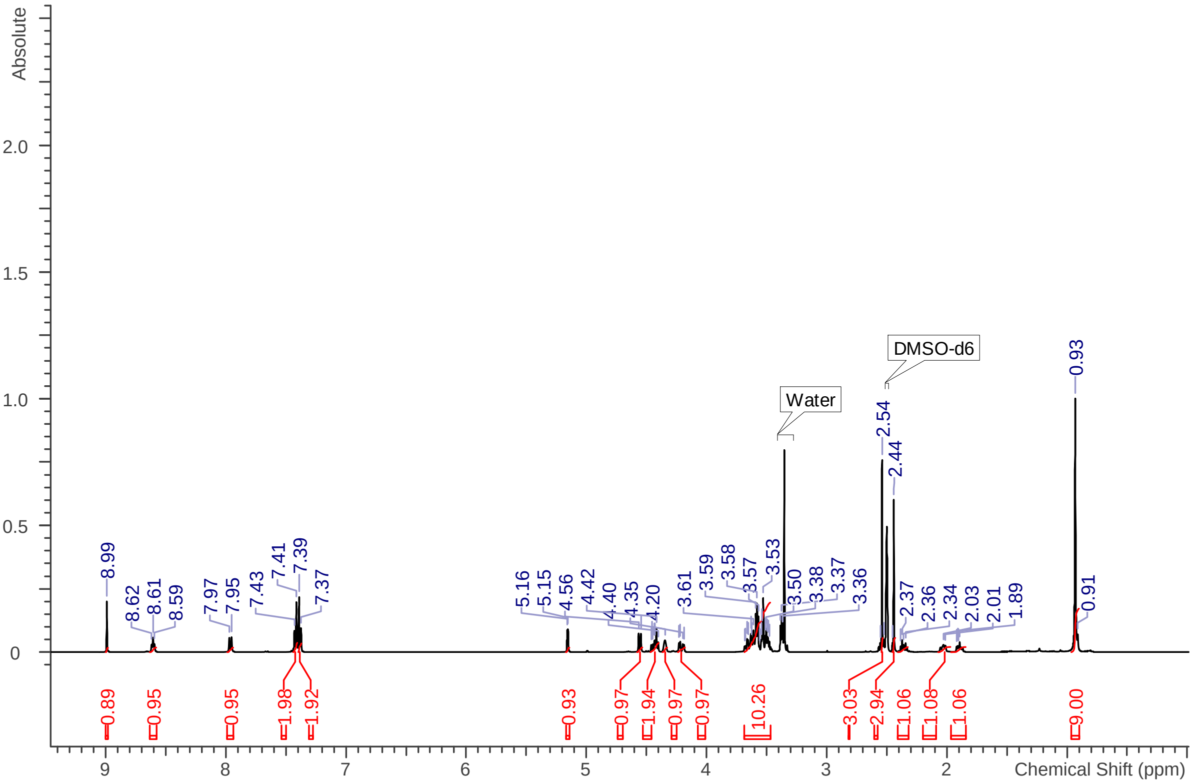

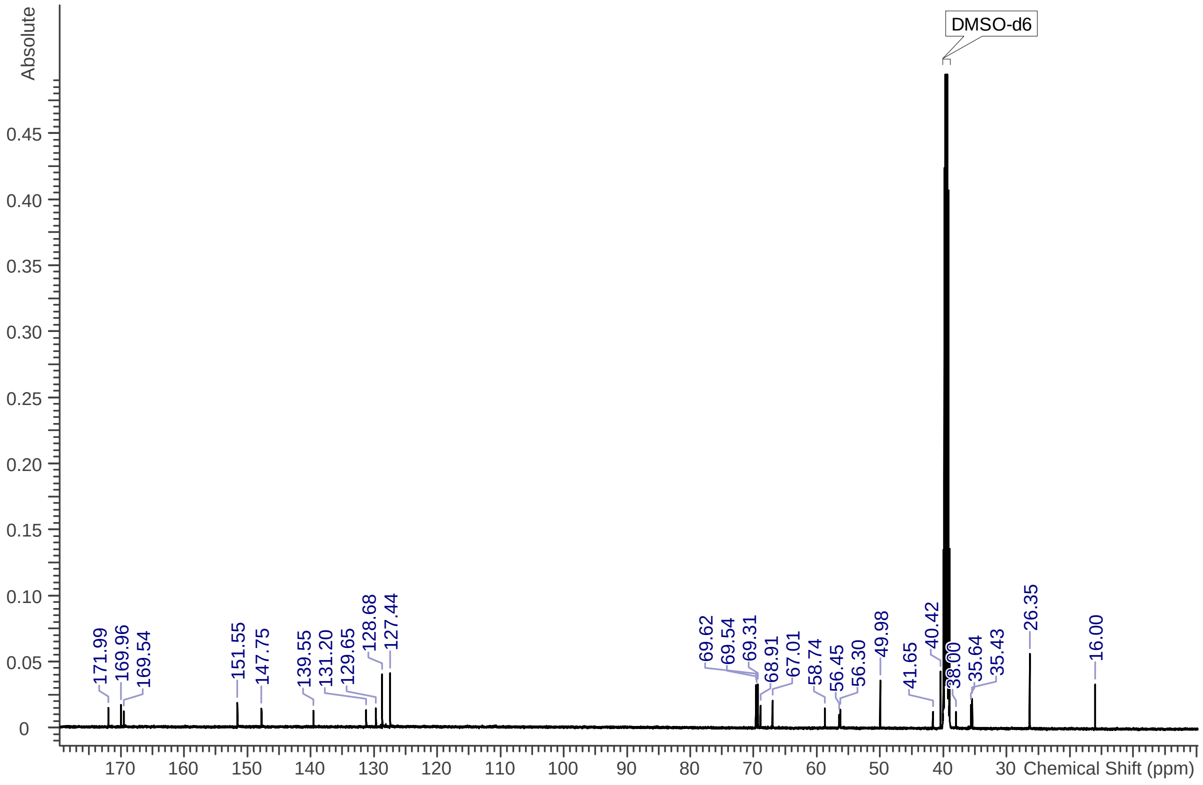

4-(1-(6-(3-(1-(4-(((S)-1-((2S,4R)-4-hydroxy-2-((4-(4-methylthiazol-5-yl)benzyl)carbamoyl)pyrolidin-1-yl)-3,3-dimethyl-1-oxobutan-2-yl)amino)-4-oxobutyl)-1H-1,2,3-triazol-4-yl)phenoxy)-4-methylpyridin-3-yl)-1H-1,2,3-triazol-4-yl)benzoic acid (**1**)

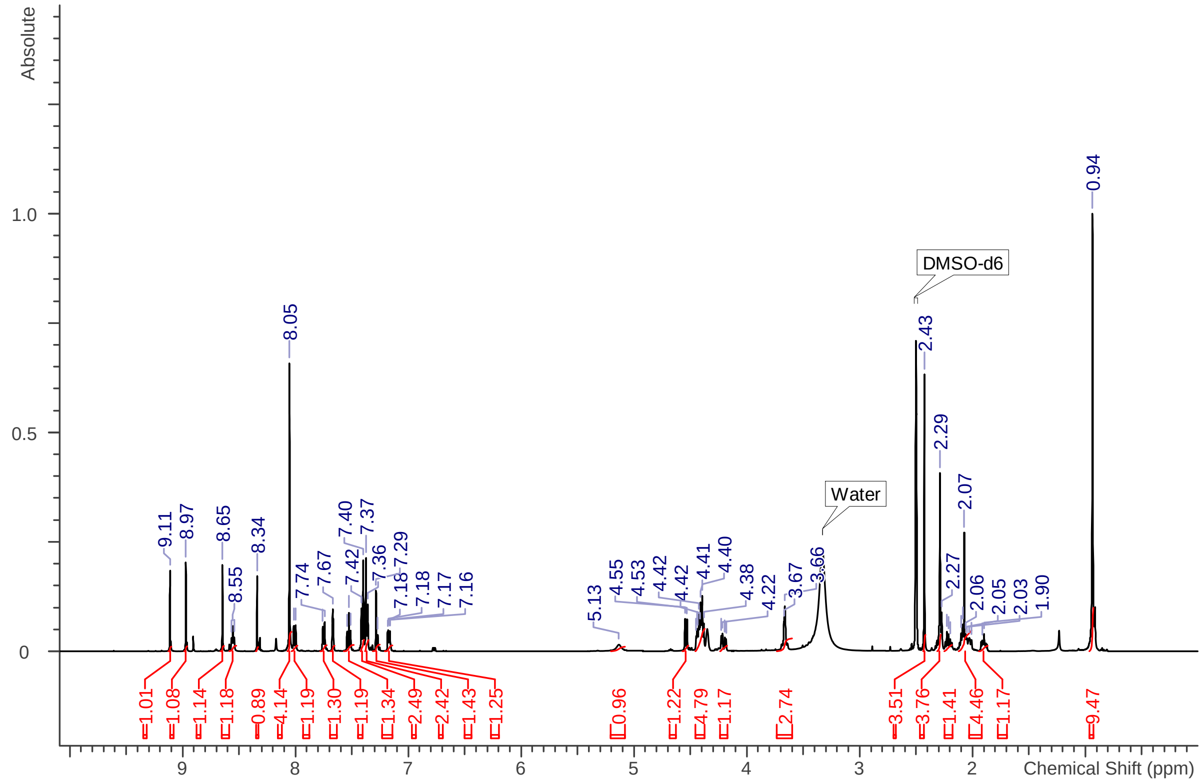

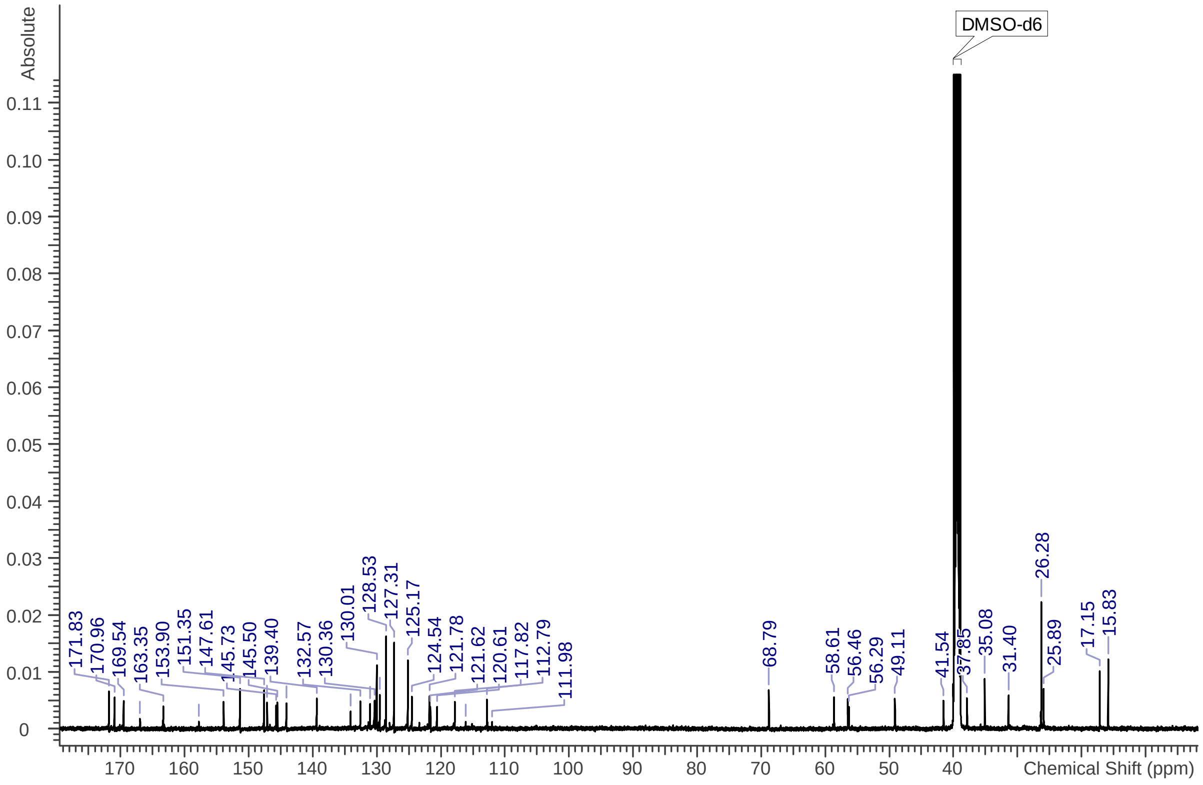

4-(1-(6-(3-(1-(4-(((S)-1-((2S,4R)-4-hydroxy-2-((4-(4-methylthiazol-5-yl)benzyl)carbamoyl)pyrolidin-1-yl)-3,3-dimethyl-1-oxobutan-2-yl)amino)-4-oxobutyl)-1H-1,2,3-triazol-4-yl)phenyl)-4-methylpyridin-3-yl)-1H-1,2,3-triazol-4-yl)benzoic acid (**3**)

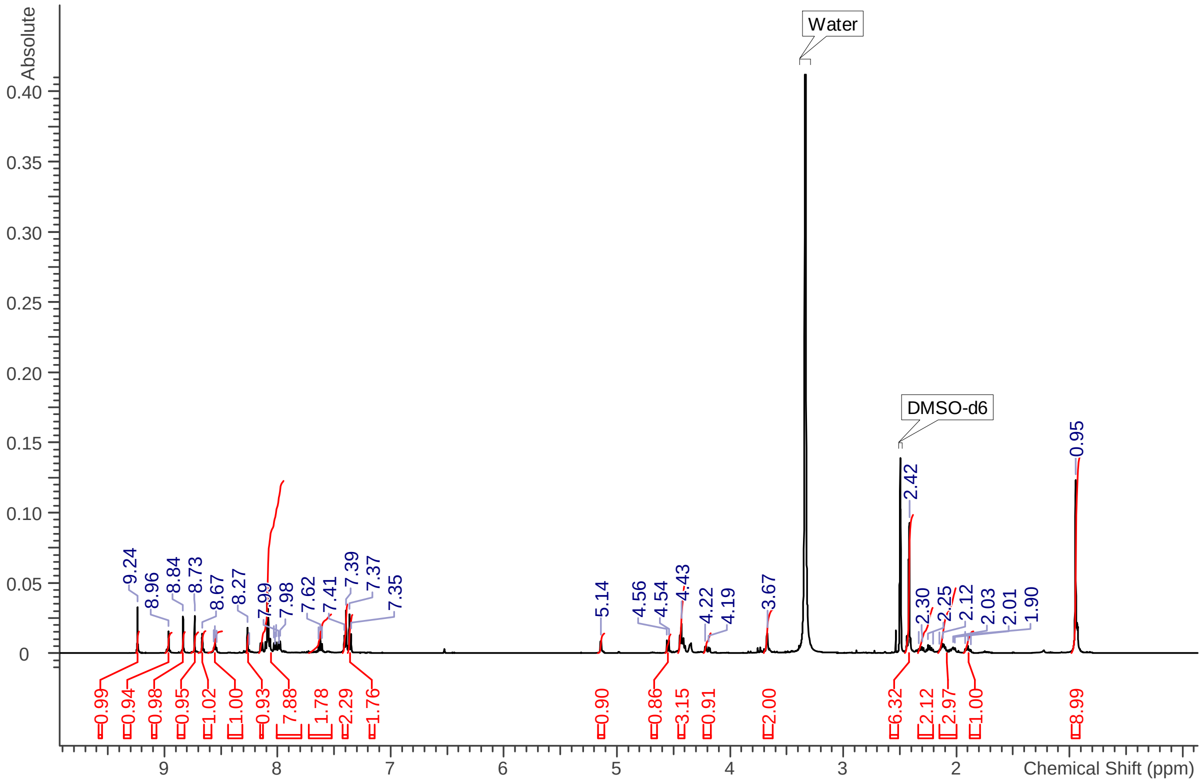

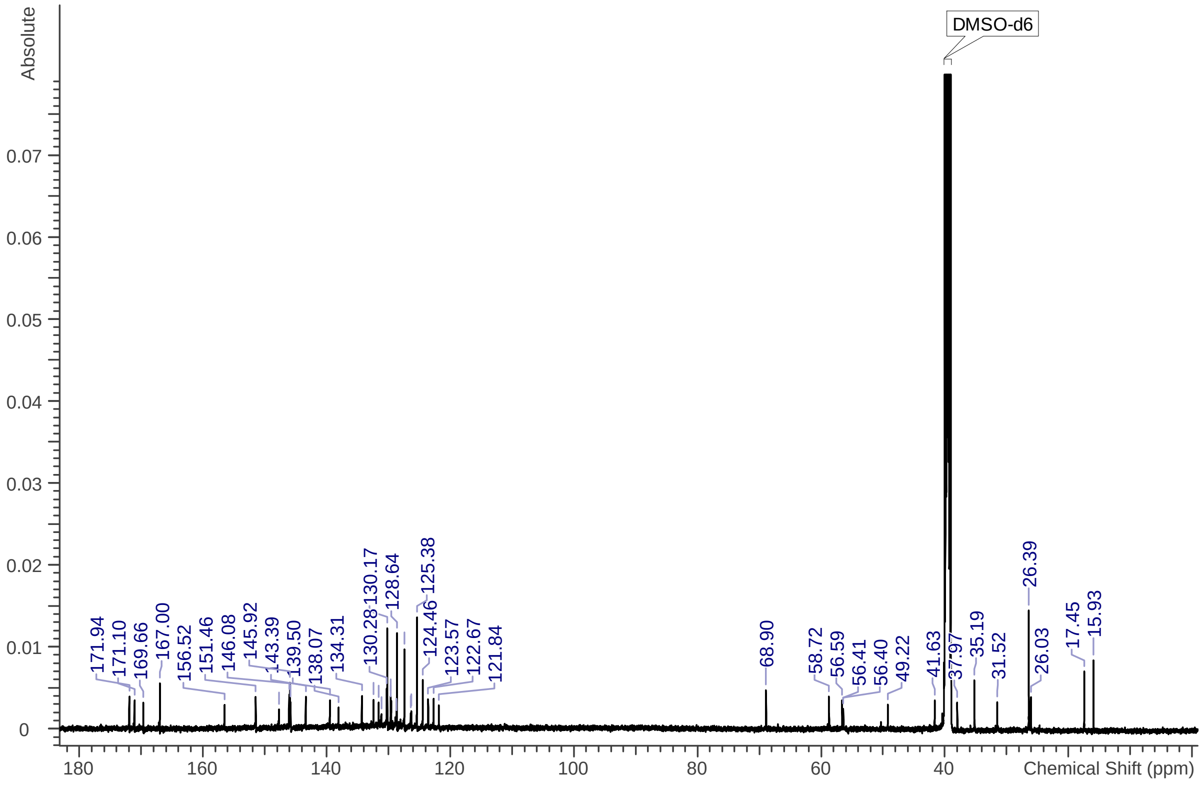

4-(1-(6-(3-(1-(2-(2-(3-(((S)-1-((2S,4R)-4-hydroxy-2-((4-(4-methylthiazol-5-yl)benzyl)carbamoyl) pyrolidin-1-yl)-3,3-dimethyl-1-oxobutan-2-yl)amino)-3-oxopropoxy)ethoxy)ethyl)-1H-1,2,3-triazol-4-yl)phenyl)-4-methylpyridin-3-yl)-1H-1,2,3-triazol-4-yl)benzoic acid (**5**)

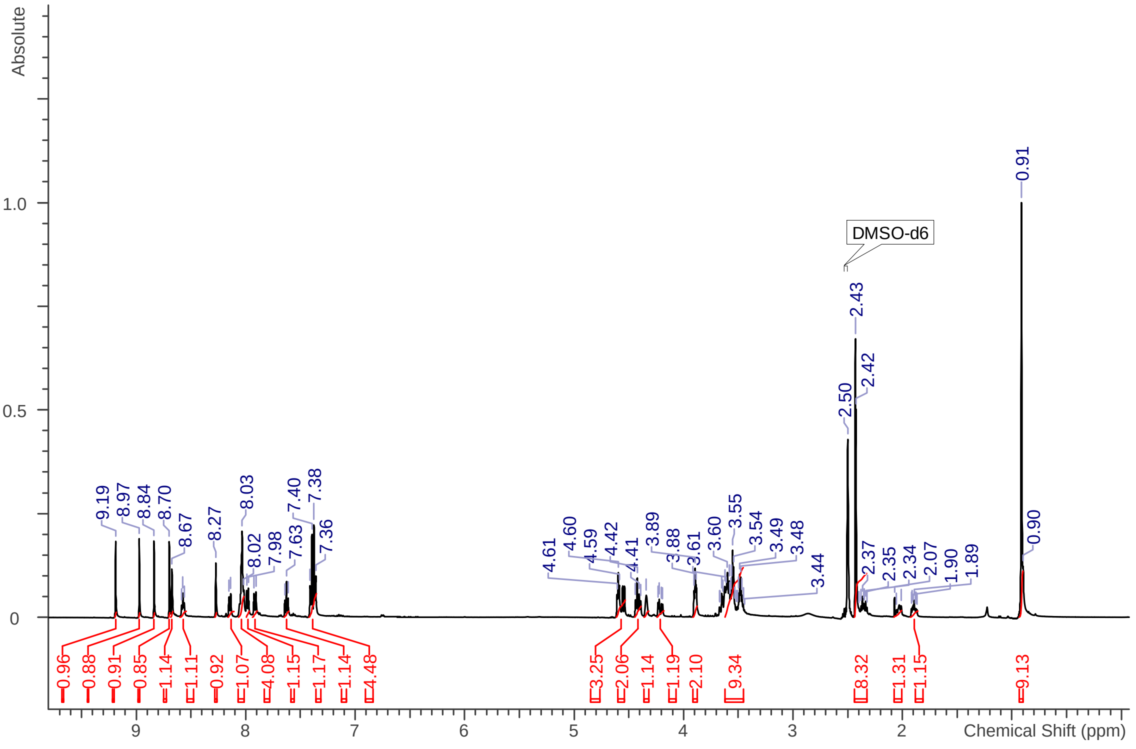

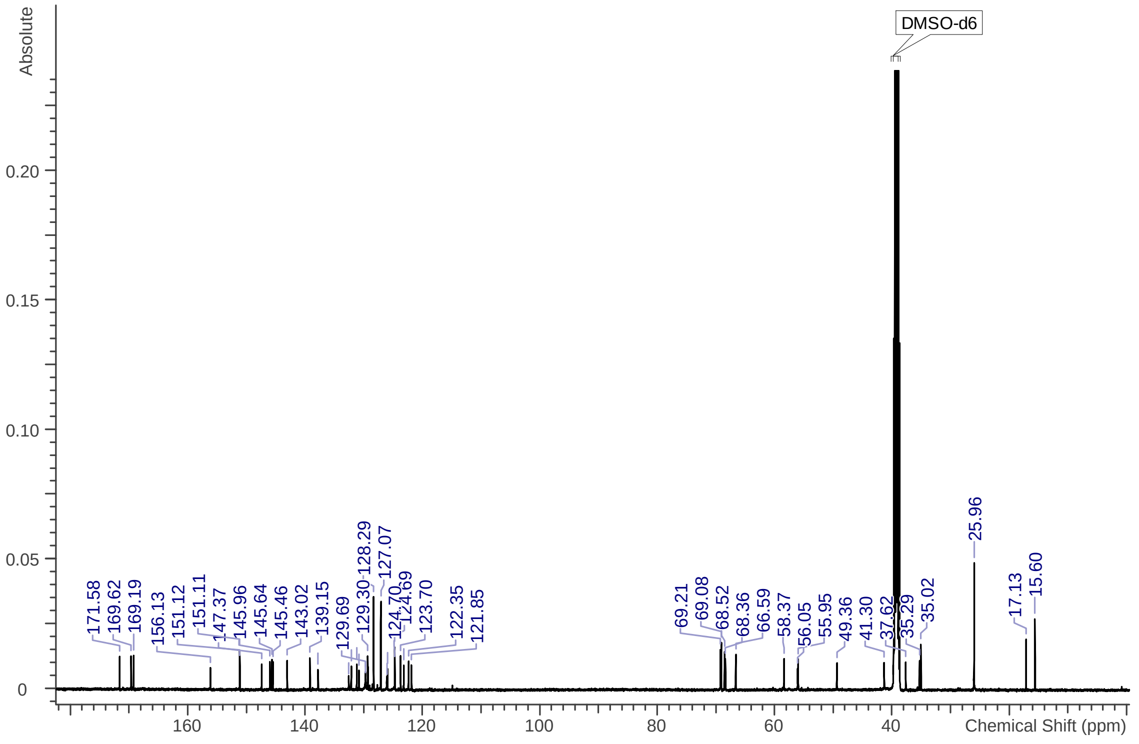

4-(1-(6-(3-(1-((S)-17-((2S,4R)-4-hydroxy-2-((4-(4-methylthiazol-5-yl)benzyl)carbamoyl)pyrolidine-1-carbonyl)-18,18-dimethyl-15-oxo-3,6,9,12-tetraoxa-16-azanonadecyl)-1H-1,2,3-tria zol-4-yl)phenyl)-4-methylpyridin-3-yl)-1H-1,2,3-triazol-4-yl)benzoic acid (**7**)

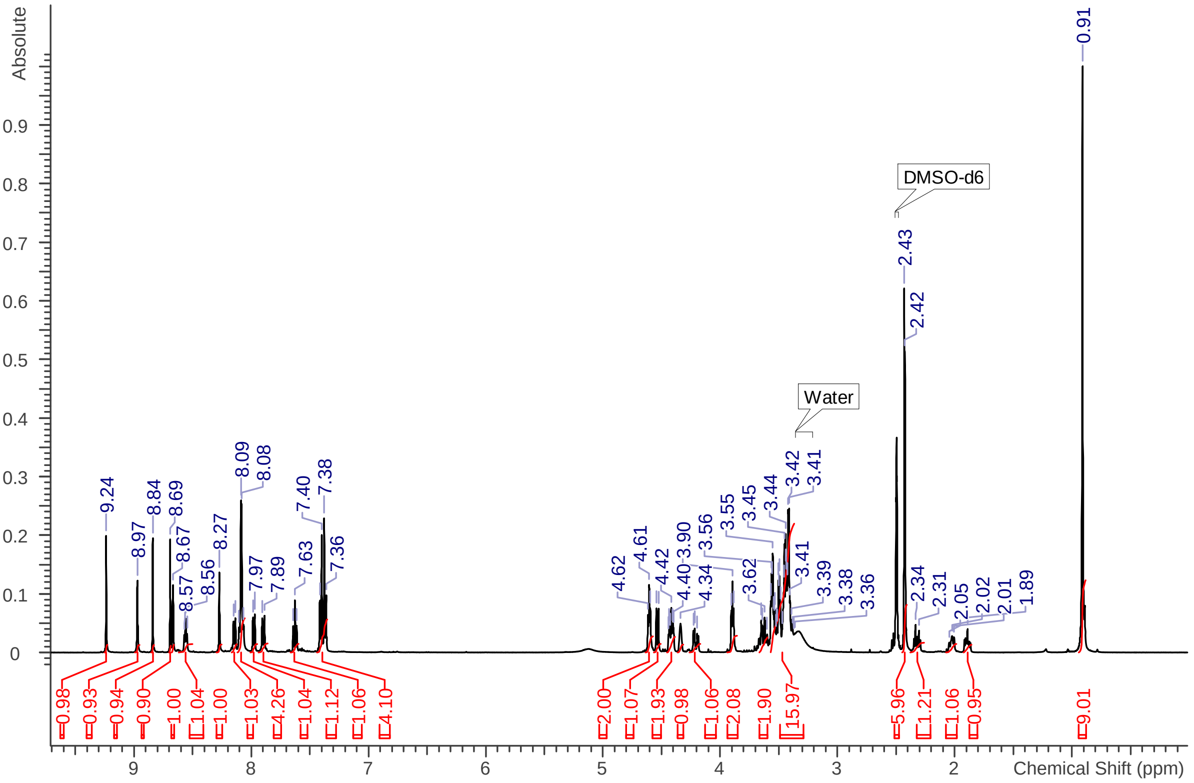

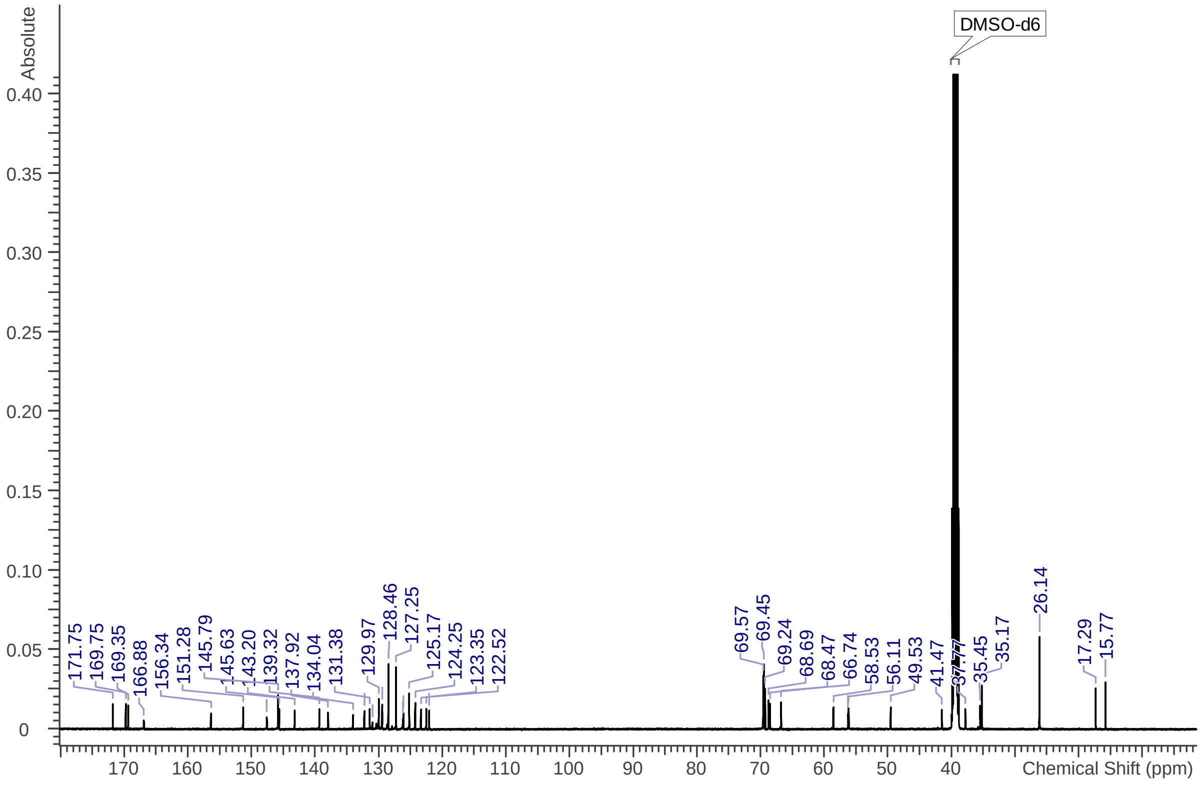

4-(1-(6-(3-(1-((S)-23-((2S,4R)-4-hydroxy-2-((4-(4-methylthiazol-5-yl)benzyl)carbamoyl)pyrolidine-1-carbonyl)-24,24-dimethyl-21-oxo-3,6,9,12,15,18-hexaoxa-22-azapentacosyl)-1H-1,2,3-triazol-4-yl)phenyl)-4-methylpyridin-3-yl)-1H-1,2,3-triazol-4-yl)benzoic acid (**9**)

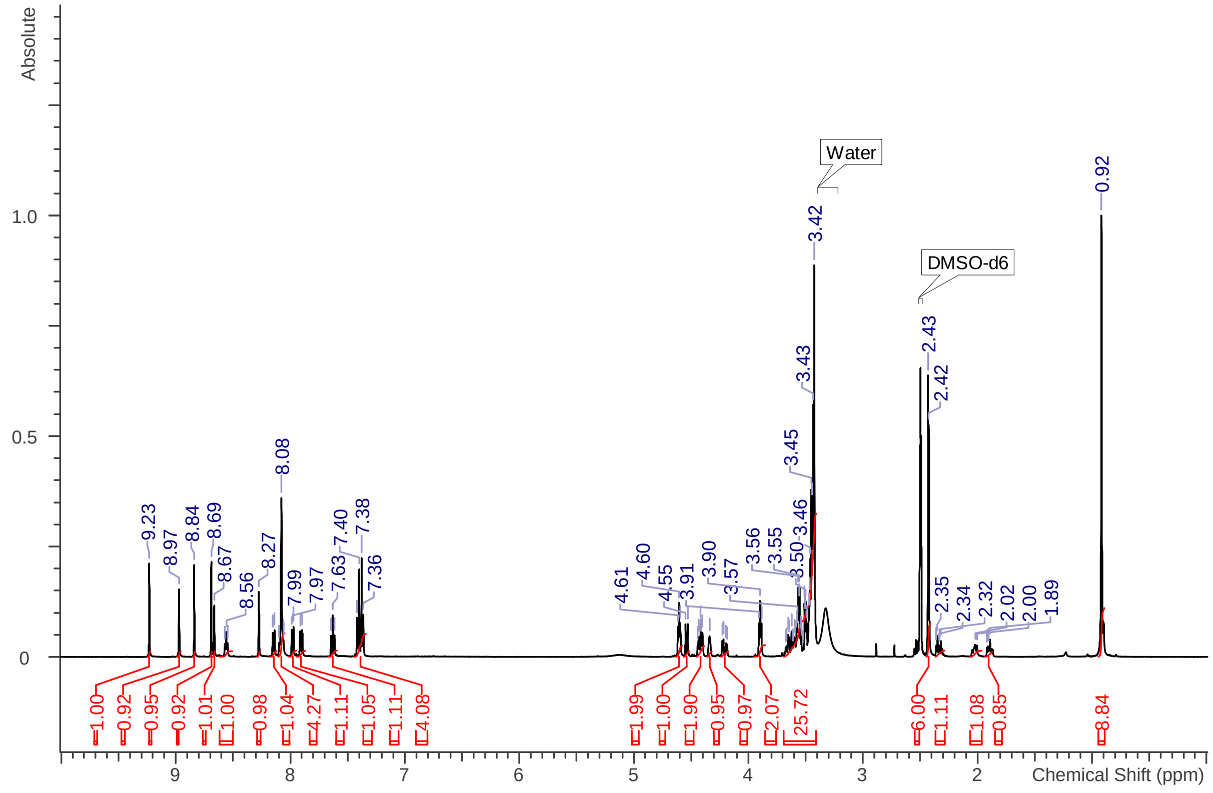

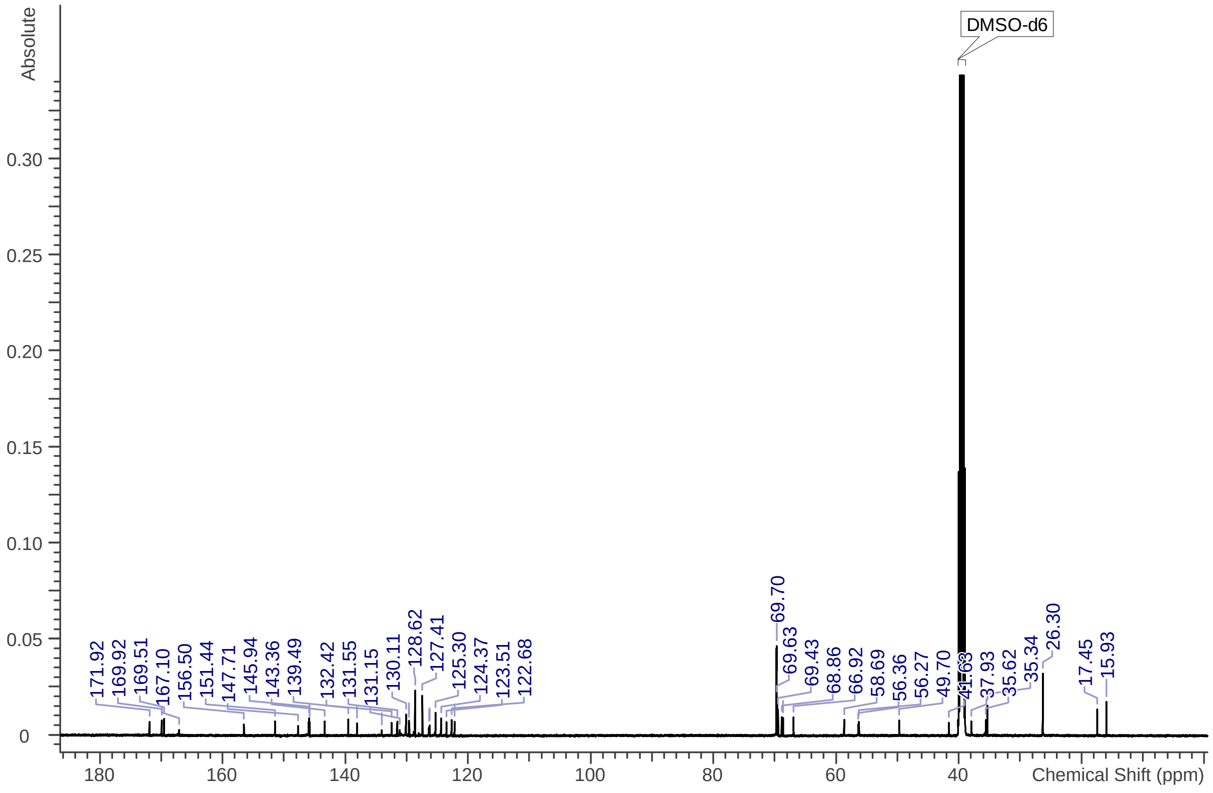

4-(1-(6-(3-(1-(4-((2-(2,6-dioxopiperidin-3-yl)-1,3-dioxoisoindolin-4-yl)amino)-4-oxobutyl)-1H-1,2,3-triazol-4-yl)phenoxy)-4-methylpyridin-3-yl)-1H-1,2,3-triazol-4-yl)benzoic acid (**2**)

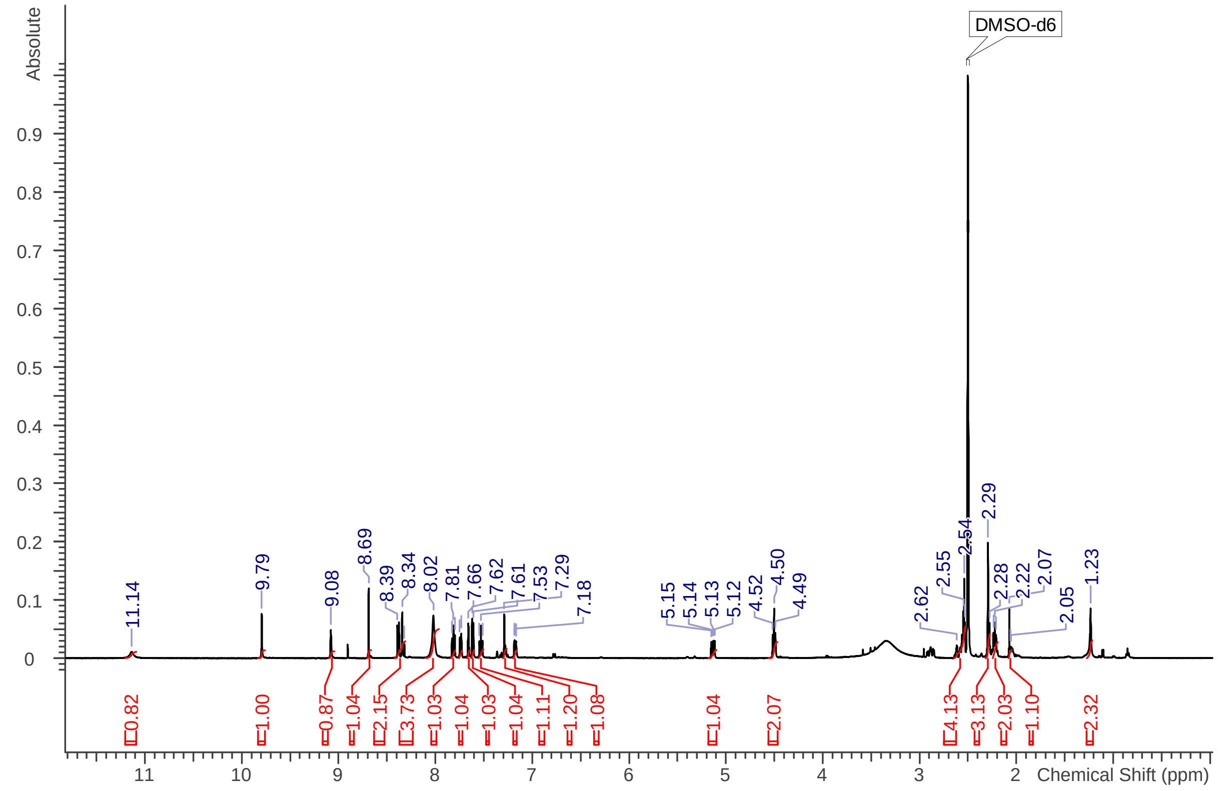

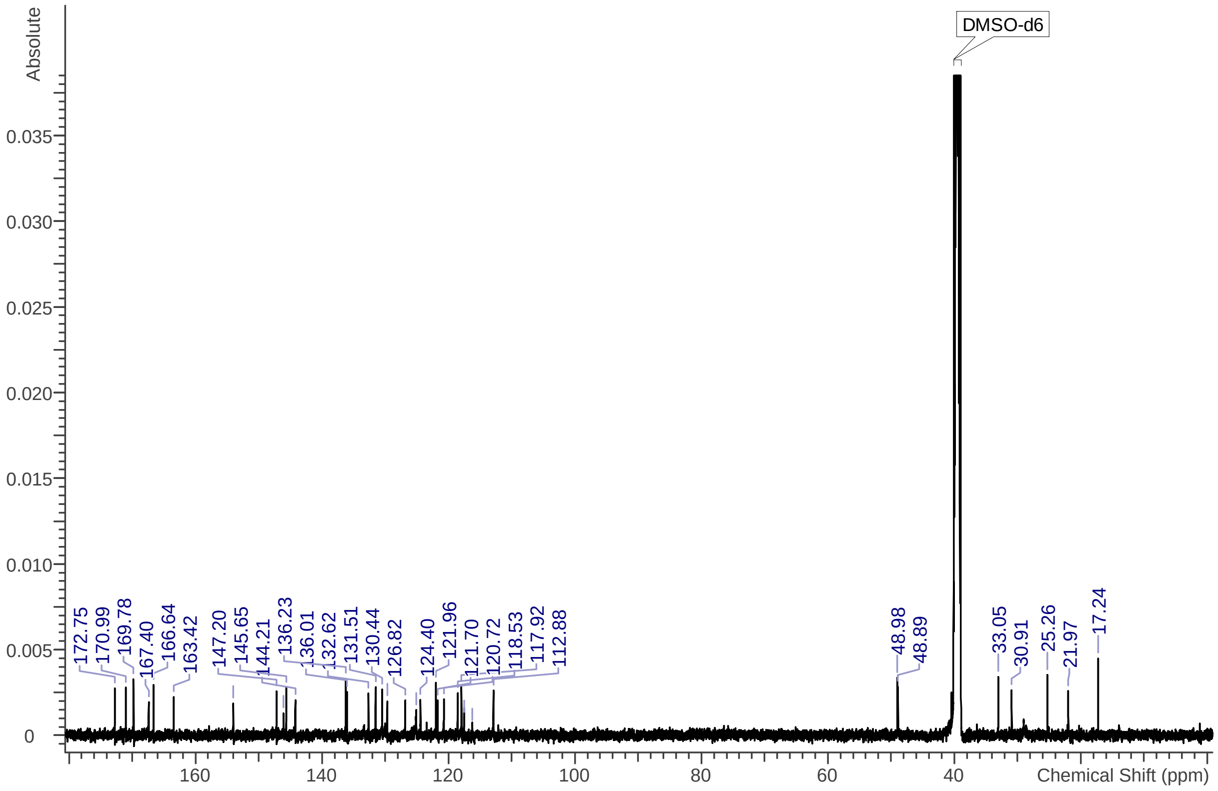

4-(1-(6-(3-(1-(4-((2-(2,6-dioxopiperidin-3-yl)-1,3-dioxoisoindolin-4-yl)amino)-4-oxobutyl)-1H-1,2,3-triazol-4-yl)phenyl)-4-methylpyridin-3-yl)-1H-1,2,3-triazol-4-yl)benzoic acid (**4**)

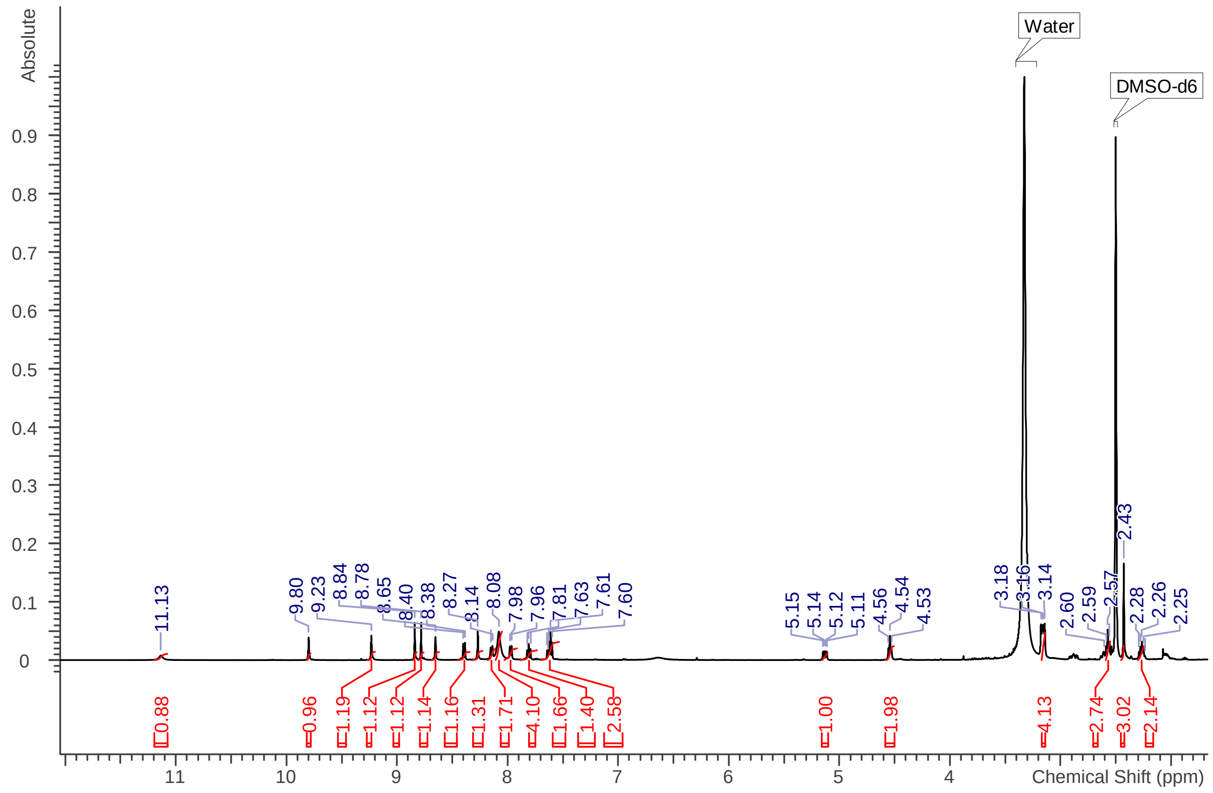

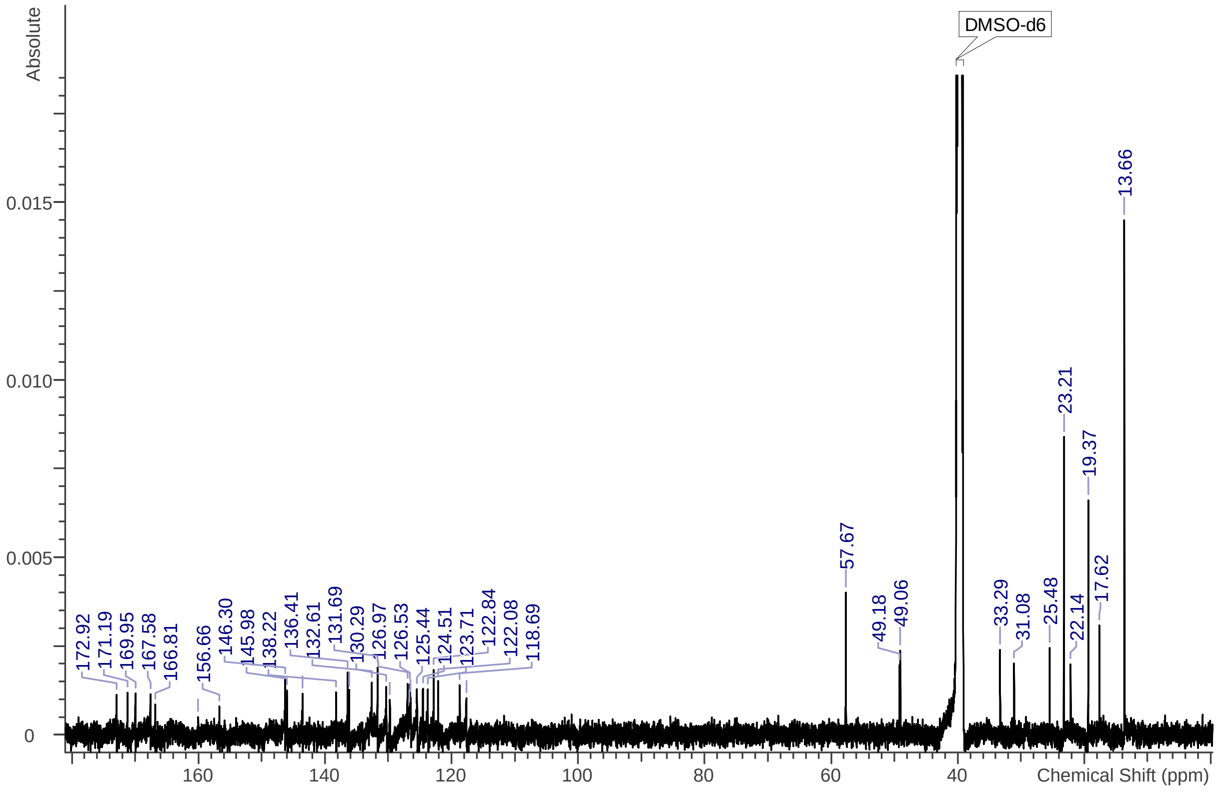

4-(1-(6-(3-(1-(2-(2-(3-((2-(2,6-dioxopiperidin-3-yl)-1,3-dioxoisoindolin-4-yl)amino)-3-oxopropoxy)ethoxy)ethyl)-1H-1,2,3-triazol-4-yl)phenyl)-4-methylpyridin-3-yl)-1H-1,2,3-triazol-4-yl) benzoic acid (**6**)

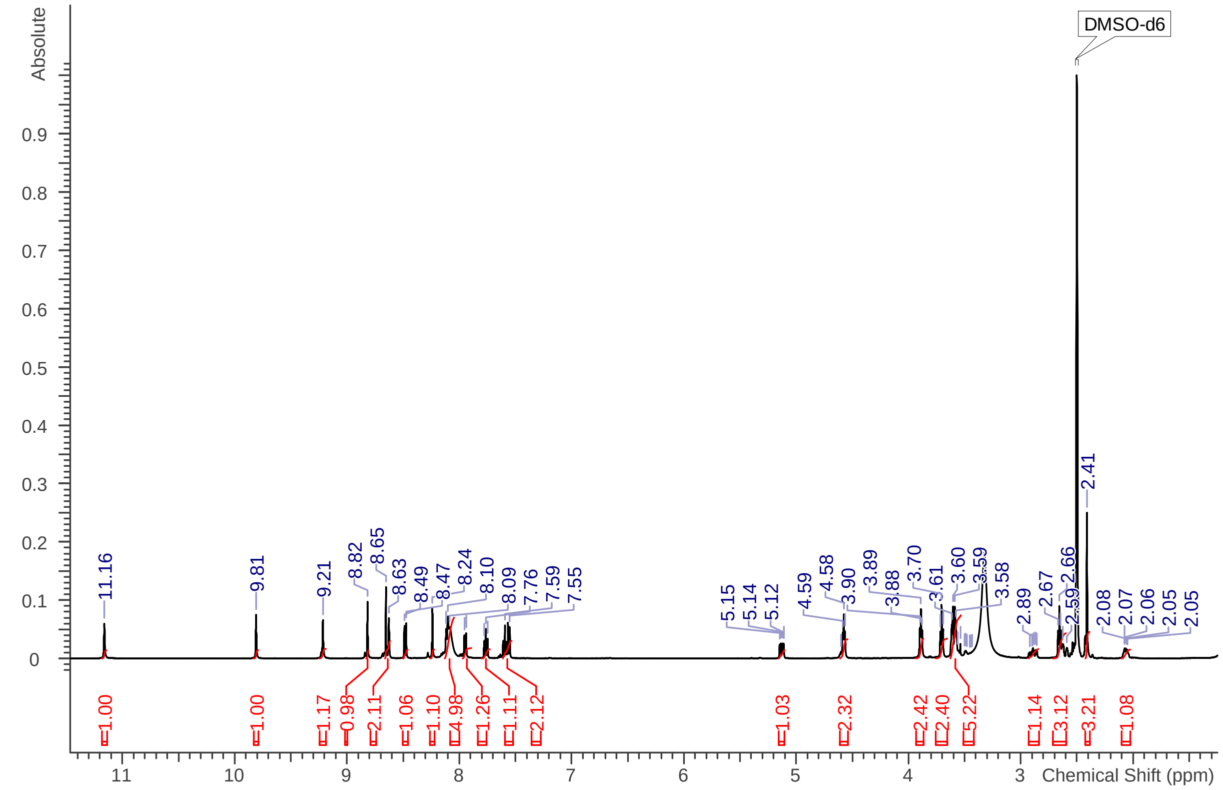

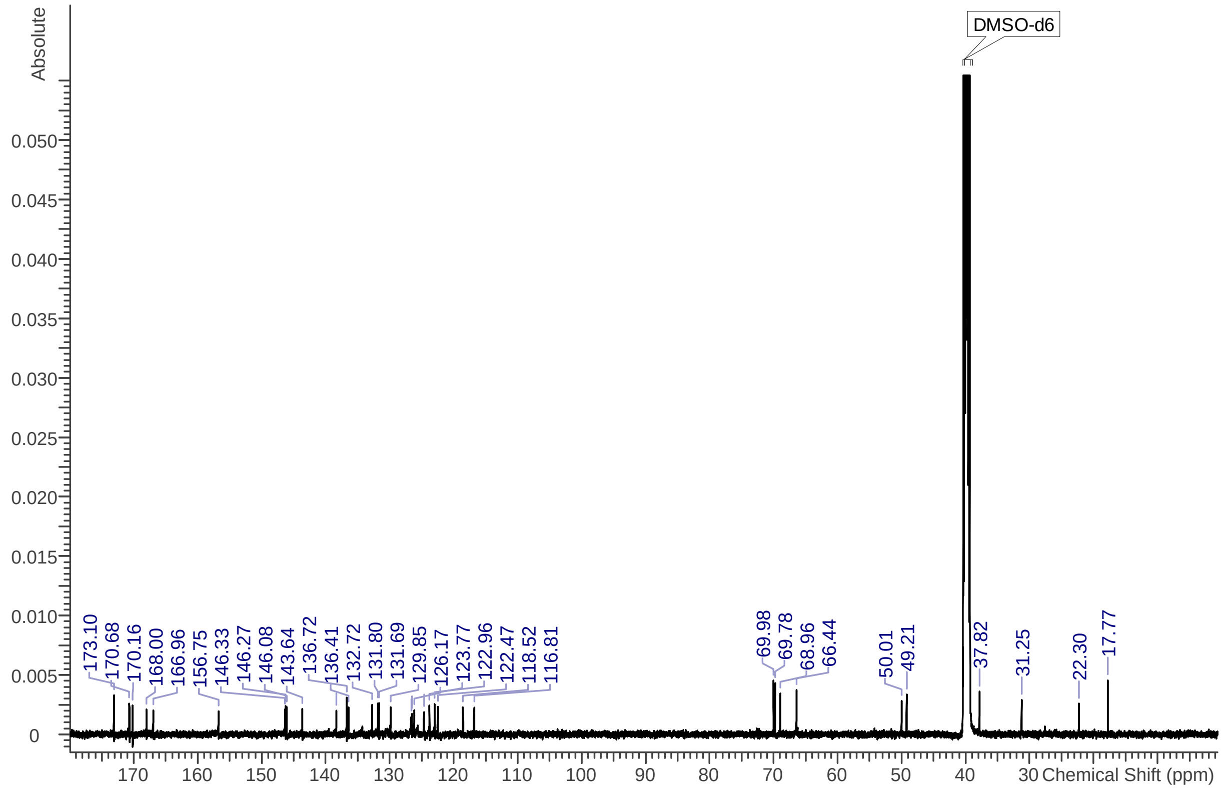

4-(1-(6-(3-(1-(15-((2-(2,6-dioxopiperidin-3-yl)-1,3-dioxoisoindolin-4-yl)amino)-15-oxo-3,6,9,12-tetraoxapentadecyl)-1H-1,2,3-triazol-4-yl)phenyl)-4-methylpyridin-3-yl)-1H-1,2,3-triazol-4-yl)benzoic acid (**8**)

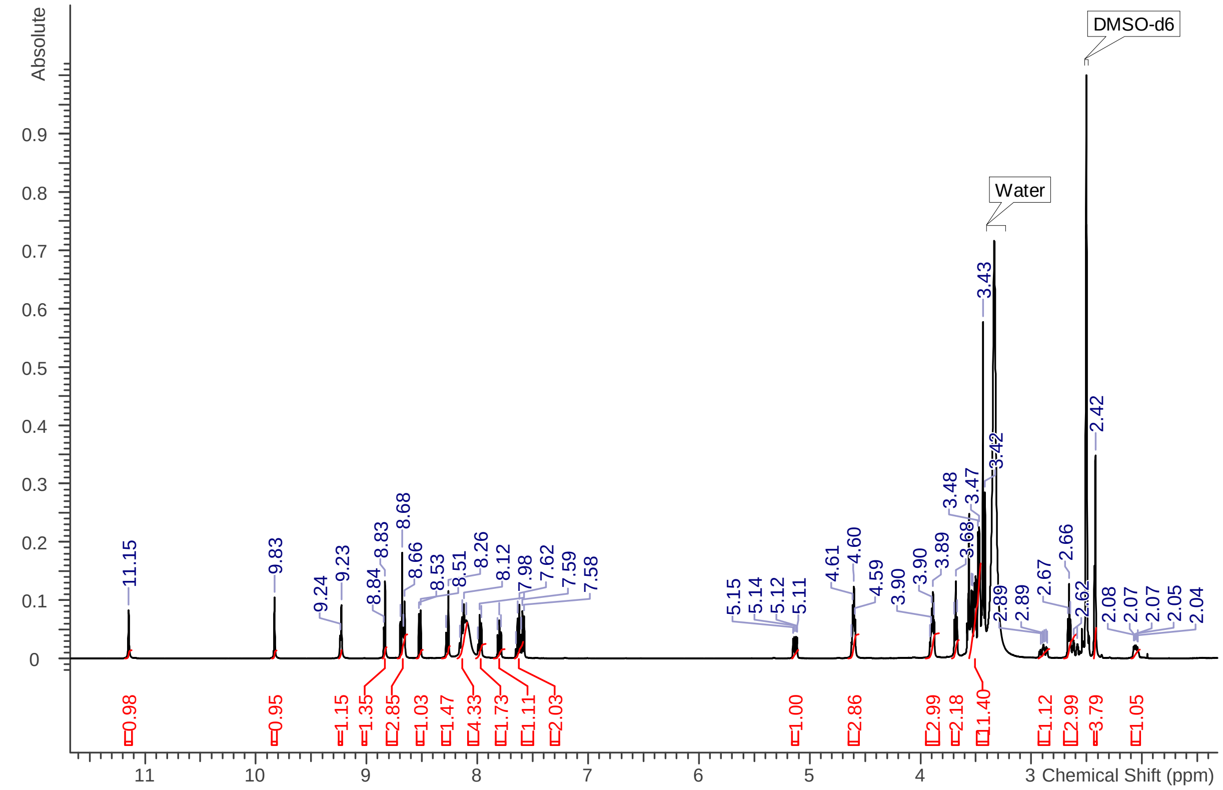

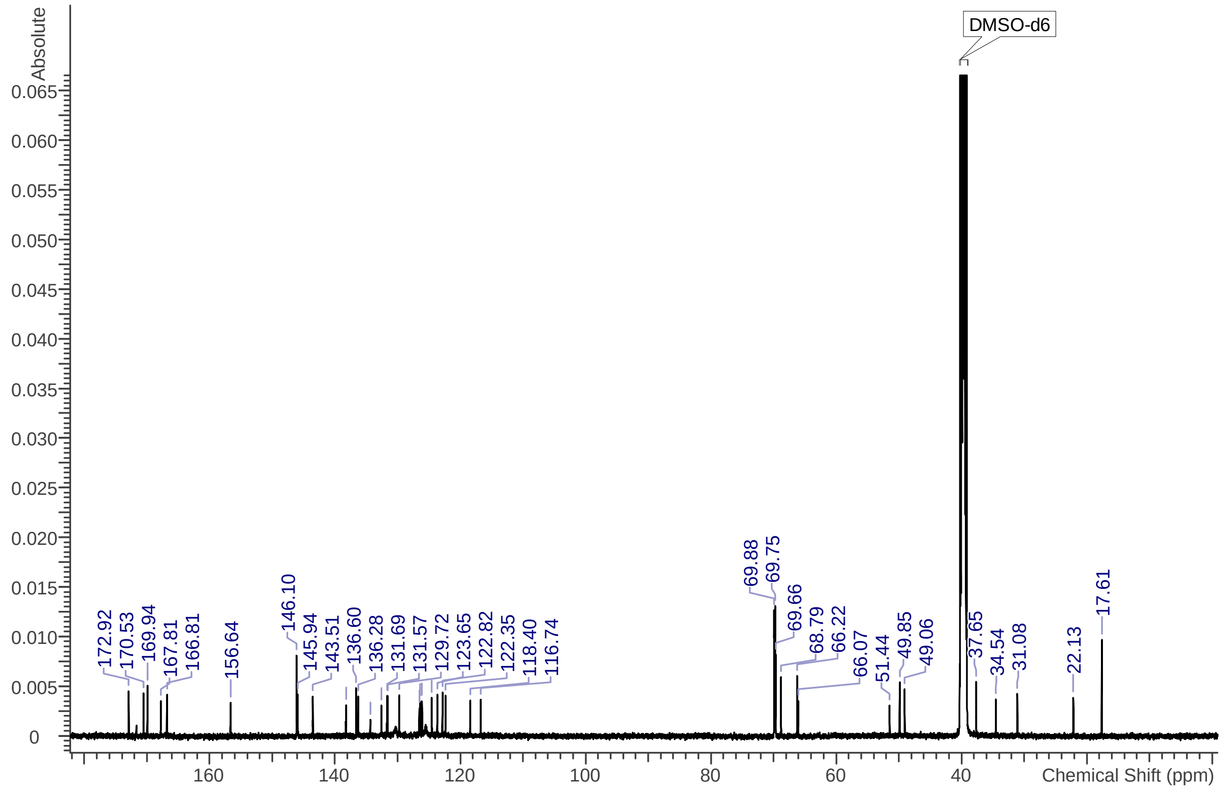

4-(1-(6-(3-(1-(21-((2-(2,6-dioxopiperidin-3-yl)-1,3-dioxoisoindolin-4-yl)amino)-21-oxo-3,6,9,12,15,18-hexaoxahenicosyl)-1H-1,2,3-triazol-4-yl)phenyl)-4-methylpyridin-3-yl)-1H-1,2,3-triazol-4-yl)benzoic acid (**10**)

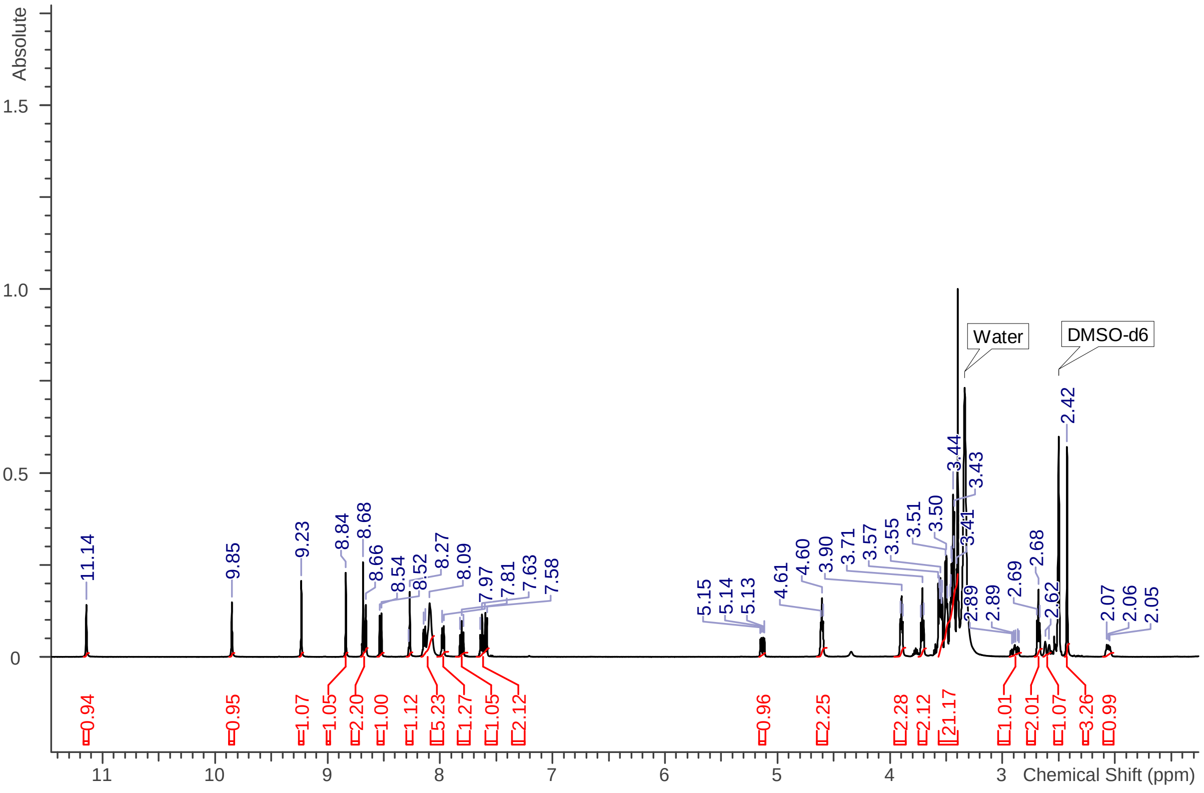

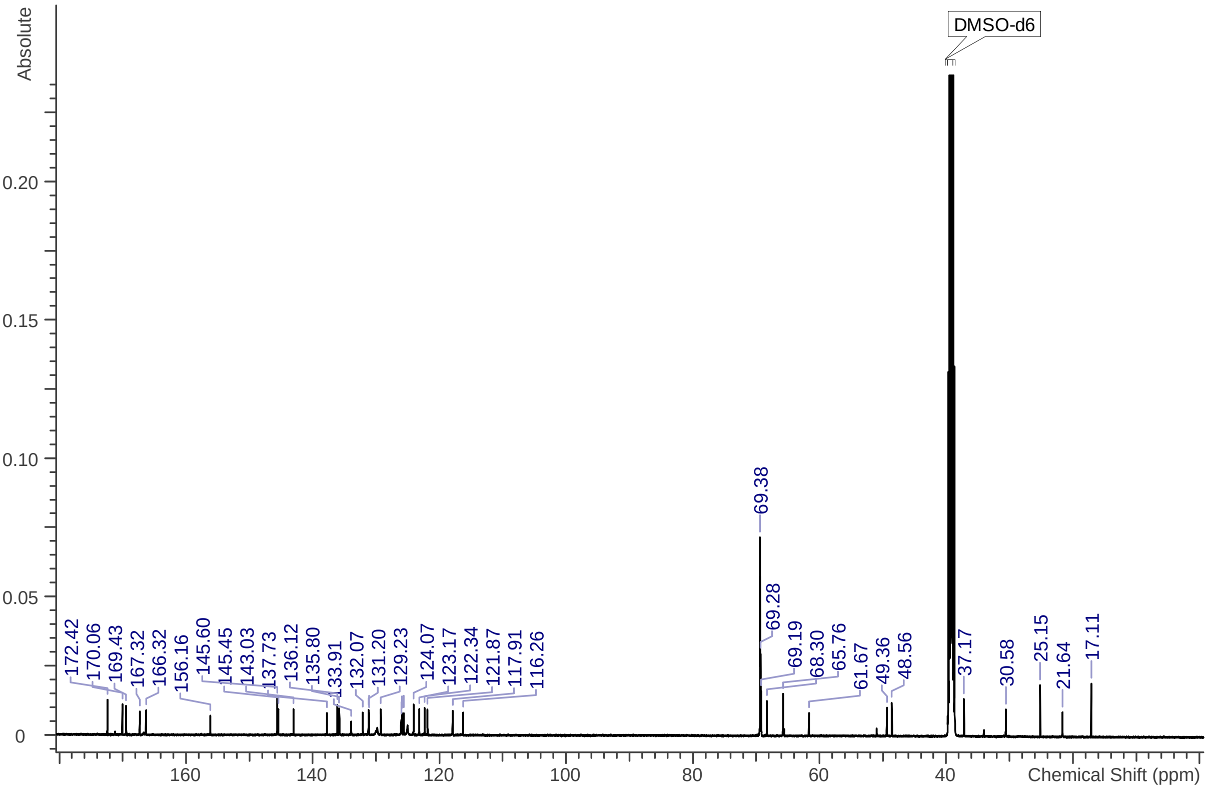

3. High Resolution Mass Spectra

4-(1-(6-(3-ethynylphenoxy)-4-methylpyridin-3-yl)-1H-1,2,3-triazol-4-yl)benzoic acid (**14**)

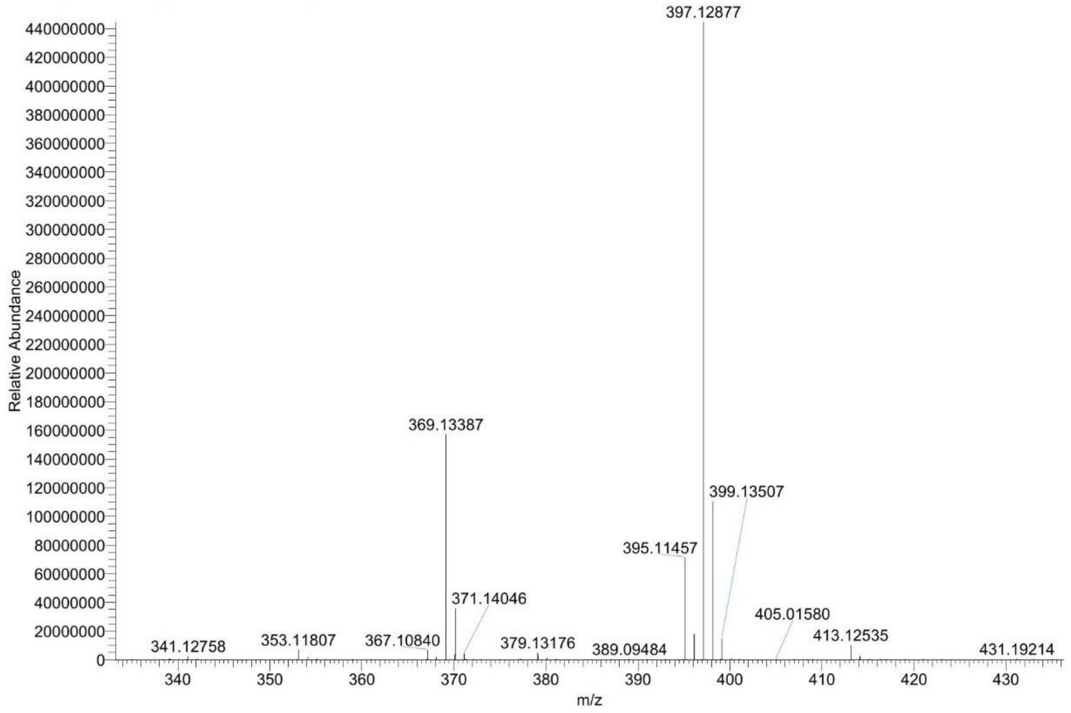

4-(1-(6-(3-formylphenyl)-4-methylpyridin-3-yl)-1H-1,2,3-triazol-4-yl)benzoic acid (**15**)

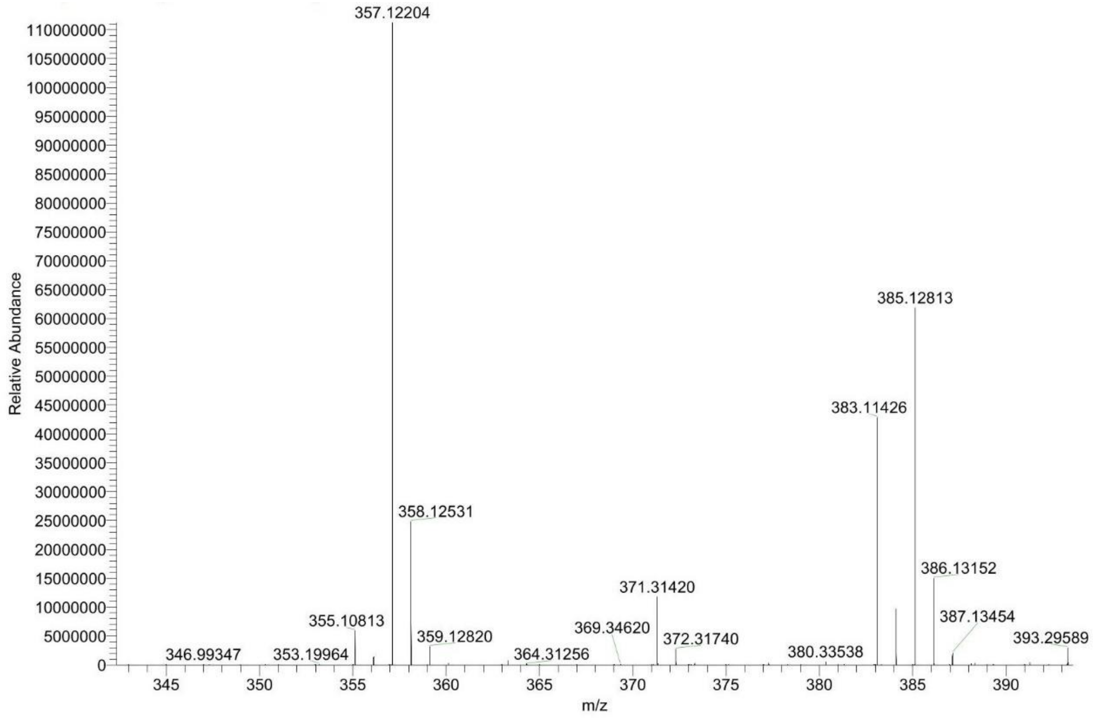

4-(1-(6-(3-ethynylphenyl)-4-methylpyridin-3-yl)-1H-1,2,3-triazol-4-yl)benzoic acid (**16**)

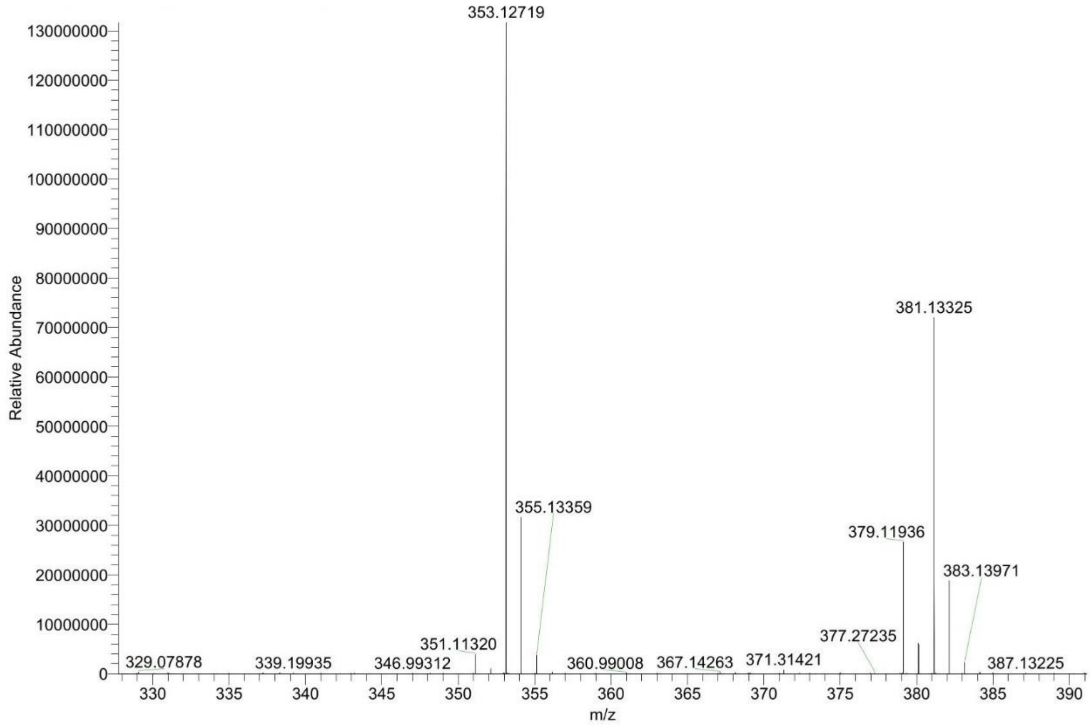

(2S,4R)-1-((S)-2-(3-(2-(2-azidoethoxy)ethoxy)propanamido)-3,3-dimethylbutanoyl)-4-hydroxy-N-(4-(4-methyl thiazol-5-yl)benzyl)pyrrolidine-2-carboxamide (**19**)

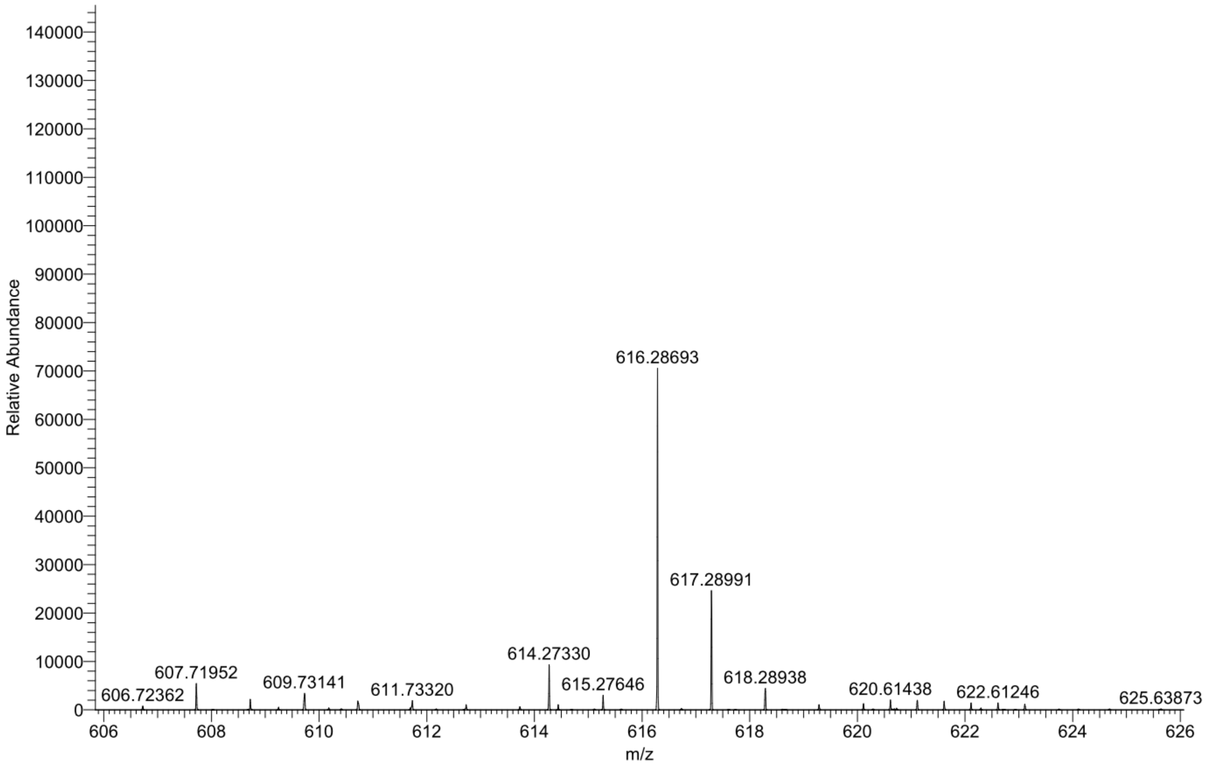

4-(1-(6-(3-(1-(4-(((S)-1-((2S,4R)-4-hydroxy-2-((4-(4-methylthiazol-5-yl)benzyl)carbamoyl)pyrolidin-1-yl)-3,3-dimethyl-1-oxobutan-2-yl)amino)-4-oxobutyl)-1H-1,2,3-triazol-4-yl)phenoxy)-4-methylpyridin-3-yl)-1H-1,2,3-triazol-4-yl)benzoic acid (**1**)

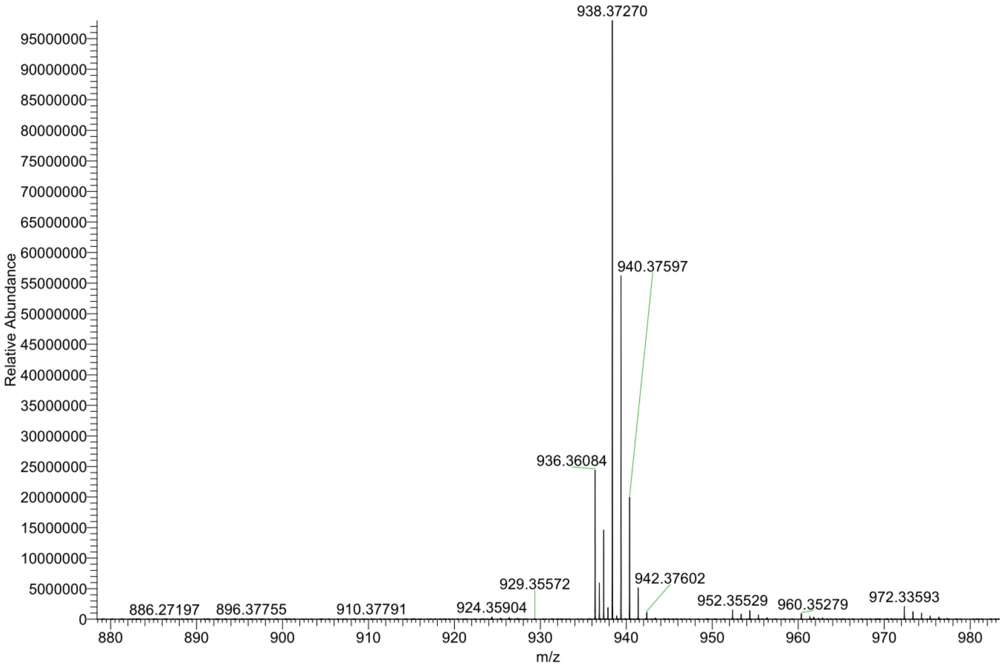

4-(1-(6-(3-(1-(4-(((S)-1-((2S,4R)-4-hydroxy-2-((4-(4-methylthiazol-5-yl)benzyl)carbamoyl)pyrolidin-1-yl)-3,3-dimethyl-1-oxobutan-2-yl)amino)-4-oxobutyl)-1H-1,2,3-triazol-4-yl)phenyl)-4-methylpyridin-3-yl)-1H-1,2,3-triazol-4-yl)benzoic acid (**3**)

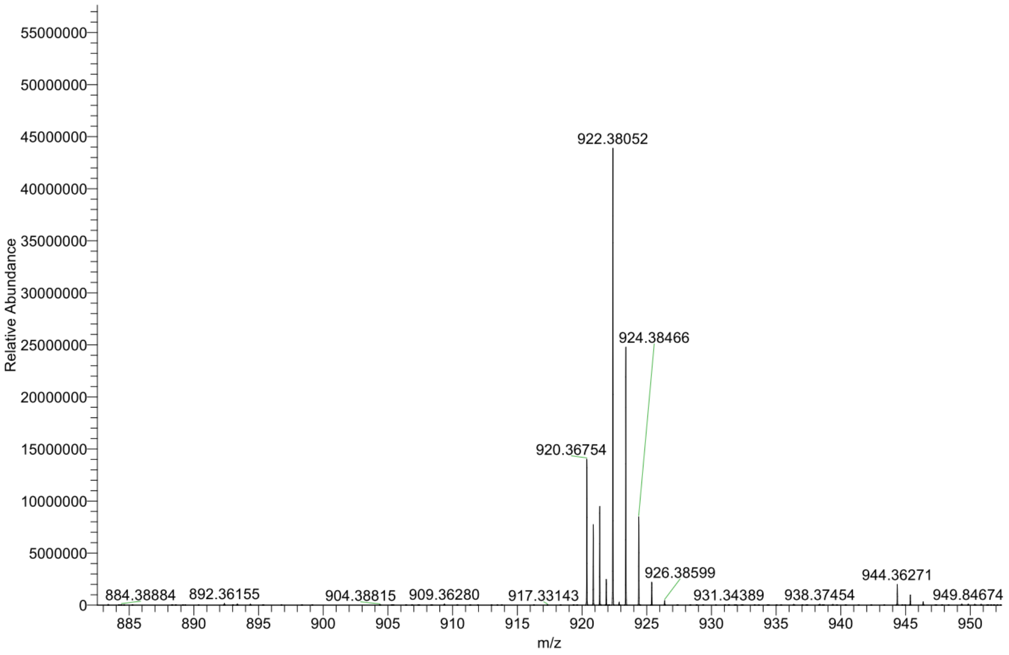

4-(1-(6-(3-(1-(2-(2-(3-(((S)-1-((2S,4R)-4-hydroxy-2-((4-(4-methylthiazol-5-yl)benzyl)carbamoyl) pyrolidin-1-yl)-3,3-dimethyl-1-oxobutan-2-yl)amino)-3-oxopropoxy)ethoxy)ethyl)-1H-1,2,3-triazol-4-yl)phenyl)-4-methylpyridin-3-yl)-1H-1,2,3-triazol-4-yl)benzoic acid (**5**)

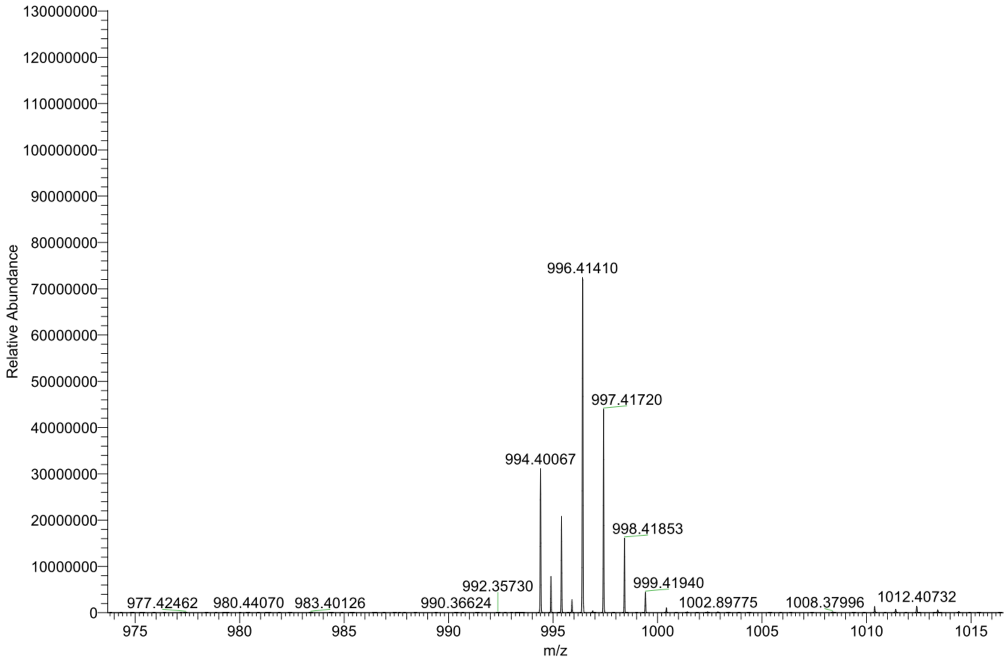

4-(1-(6-(3-(1-((S)-17-((2S,4R)-4-hydroxy-2-((4-(4-methylthiazol-5-yl)benzyl)carbamoyl)pyrolidine-1-carbonyl)-18,18-dimethyl-15-oxo-3,6,9,12-tetraoxa-16-azanonadecyl)-1H-1,2,3-tria zol-4-yl)phenyl)-4-methylpyridin-3-yl)-1H-1,2,3-triazol-4-yl)benzoic acid (**7**)

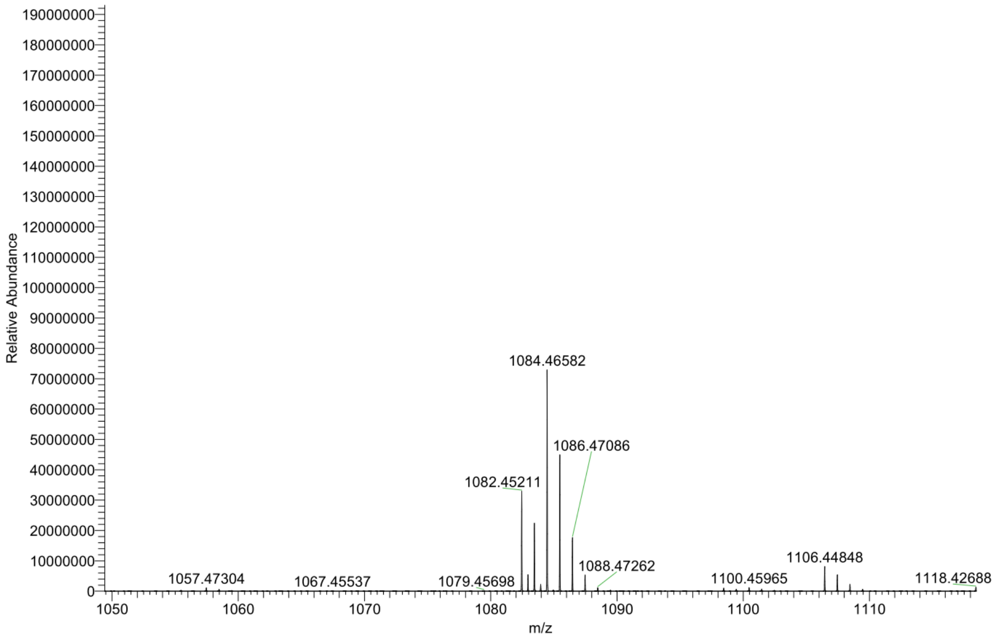

4-(1-(6-(3-(1-((S)-23-((2S,4R)-4-hydroxy-2-((4-(4-methylthiazol-5-yl)benzyl)carbamoyl)pyrolidine-1-carbonyl)-24,24-dimethyl-21-oxo-3,6,9,12,15,18-hexaoxa-22-azapentacosyl)-1H-1,2,3-triazol-4-yl)phenyl)-4-methylpyridin-3-yl)-1H-1,2,3-triazol-4-yl)benzoic acid (**9**)

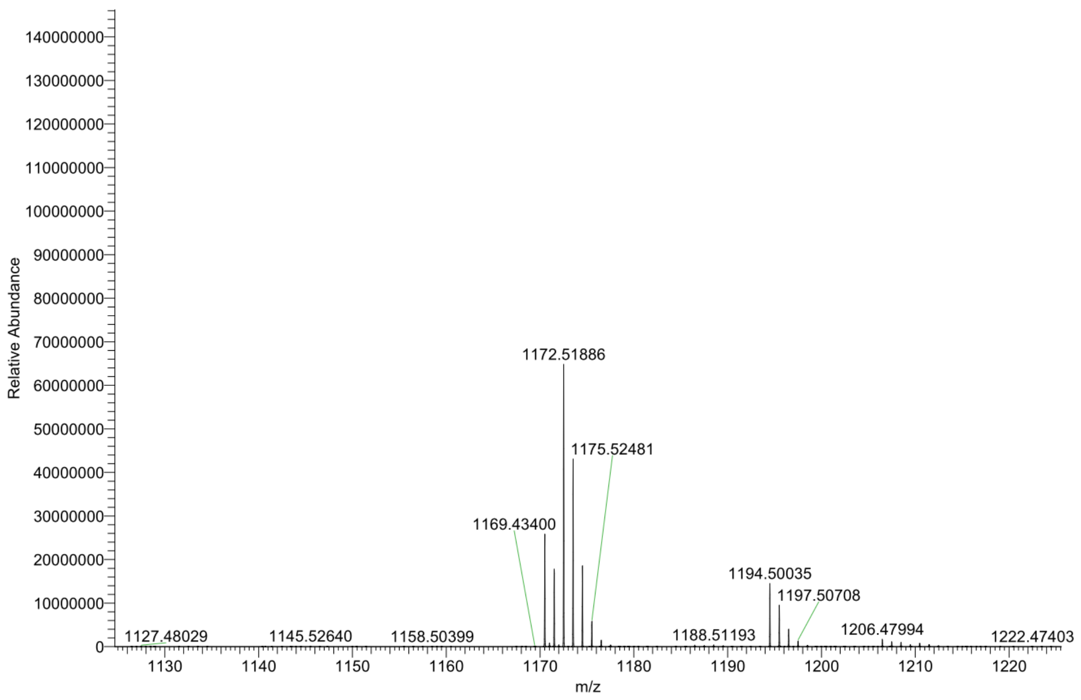

4-(1-(6-(3-(1-(4-((2-(2,6-dioxopiperidin-3-yl)-1,3-dioxoisoindolin-4-yl)amino)-4-oxobutyl)-1H-1,2,3-triazol-4-yl)phenoxy)-4-methylpyridin-3-yl)-1H-1,2,3-triazol-4-yl)benzoic acid (**2**)

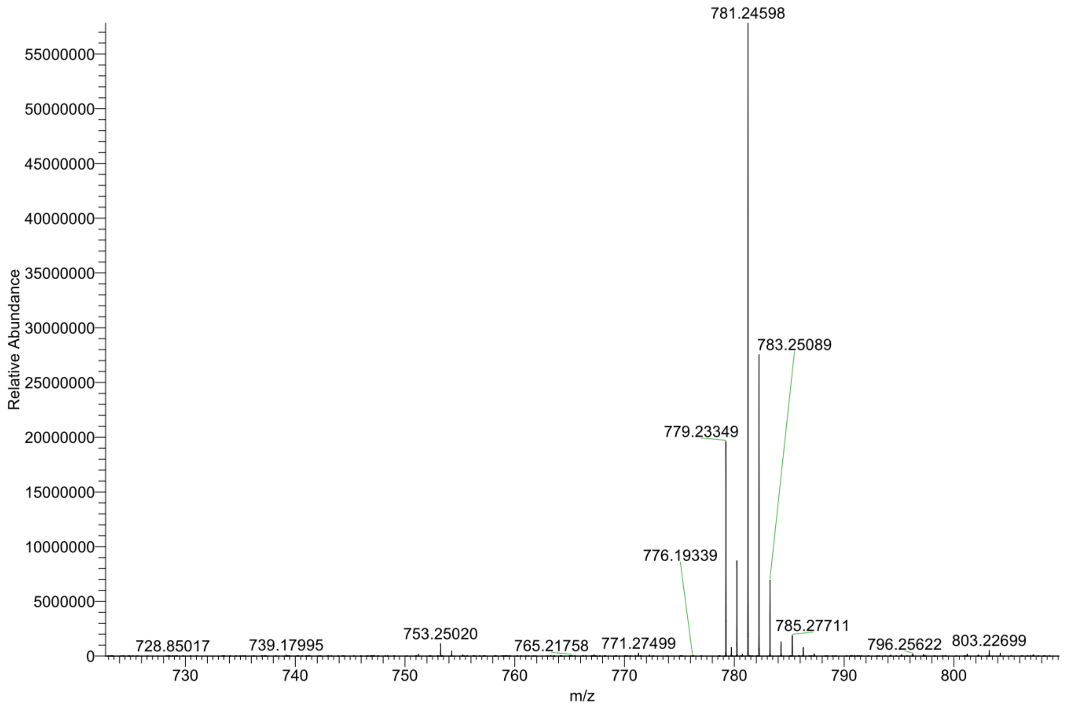

4-(1-(6-(3-(1-(4-((2-(2,6-dioxopiperidin-3-yl)-1,3-dioxoisoindolin-4-yl)amino)-4-oxobutyl)-1H-1,2,3-triazol-4-yl)phenyl)-4-methylpyridin-3-yl)-1H-1,2,3-triazol-4-yl)benzoic acid (**4**)

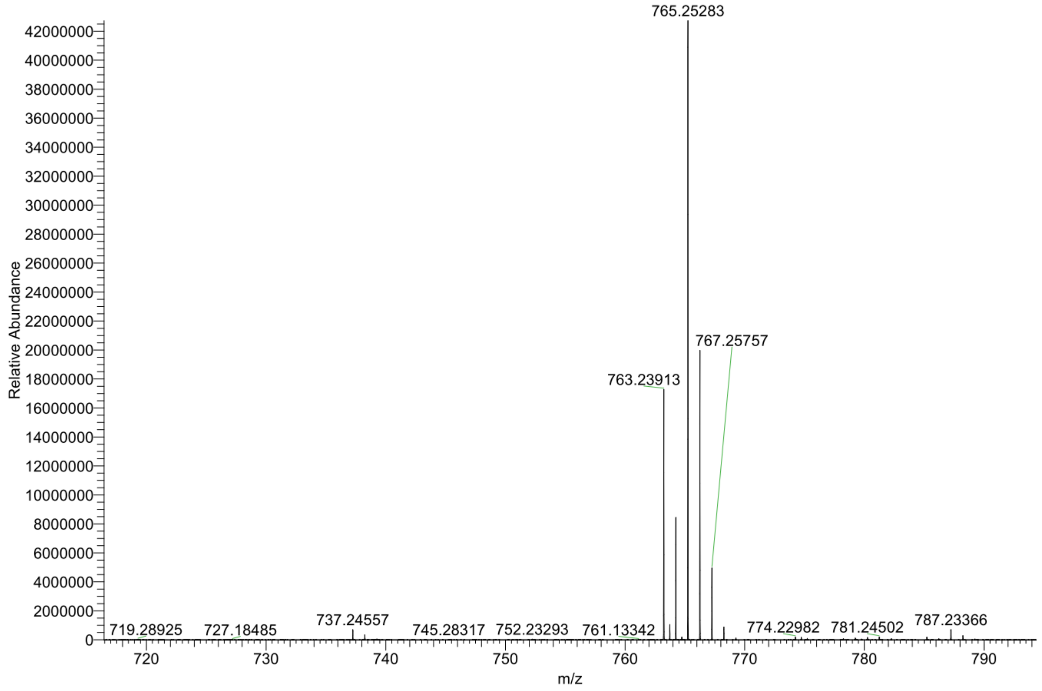

4-(1-(6-(3-(1-(2-(2-(3-((2-(2,6-dioxopiperidin-3-yl)-1,3-dioxoisoindolin-4-yl)amino)-3-oxopropoxy) ethoxy)ethyl)-1H-1,2,3-triazol-4-yl)phenyl)-4-methylpyridin-3-yl)-1H-1,2,3-triazol-4-yl) benzoic acid (**6**)

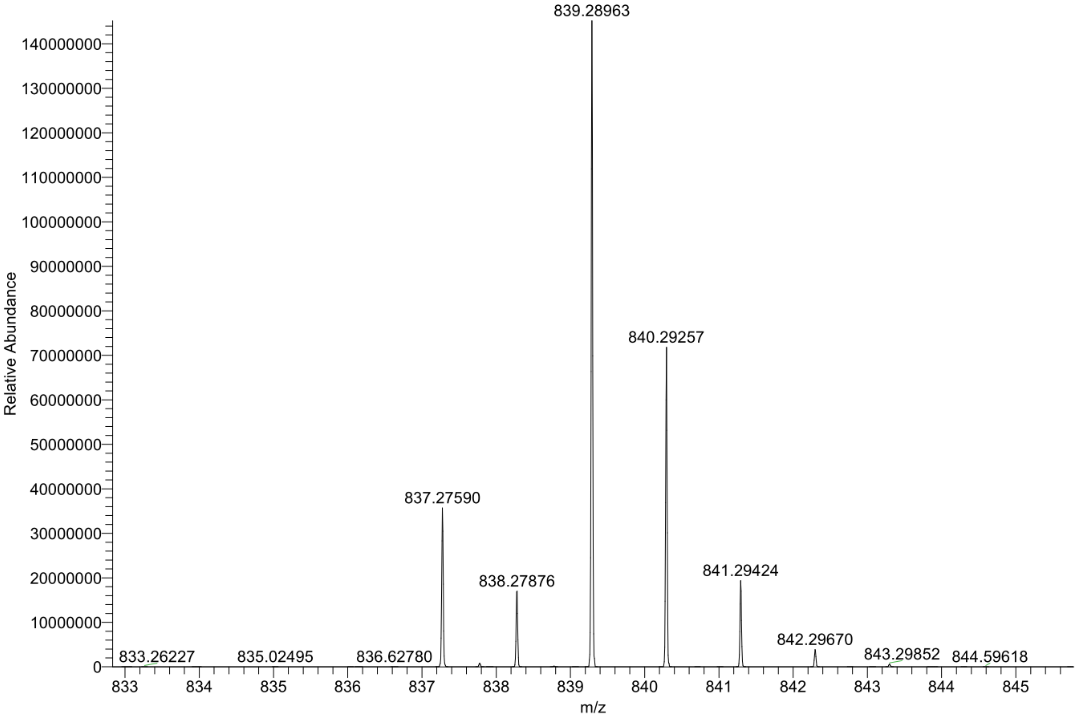

4-(1-(6-(3-(1-(15-((2-(2,6-dioxopiperidin-3-yl)-1,3-dioxoisoindolin-4-yl)amino)-15-oxo-3,6,9,12-tetraoxapentadecyl)-1H-1,2,3-triazol-4-yl)phenyl)-4-methylpyridin-3-yl)-1H-1,2,3-triazol-4-yl)benzoic acid (**8**)

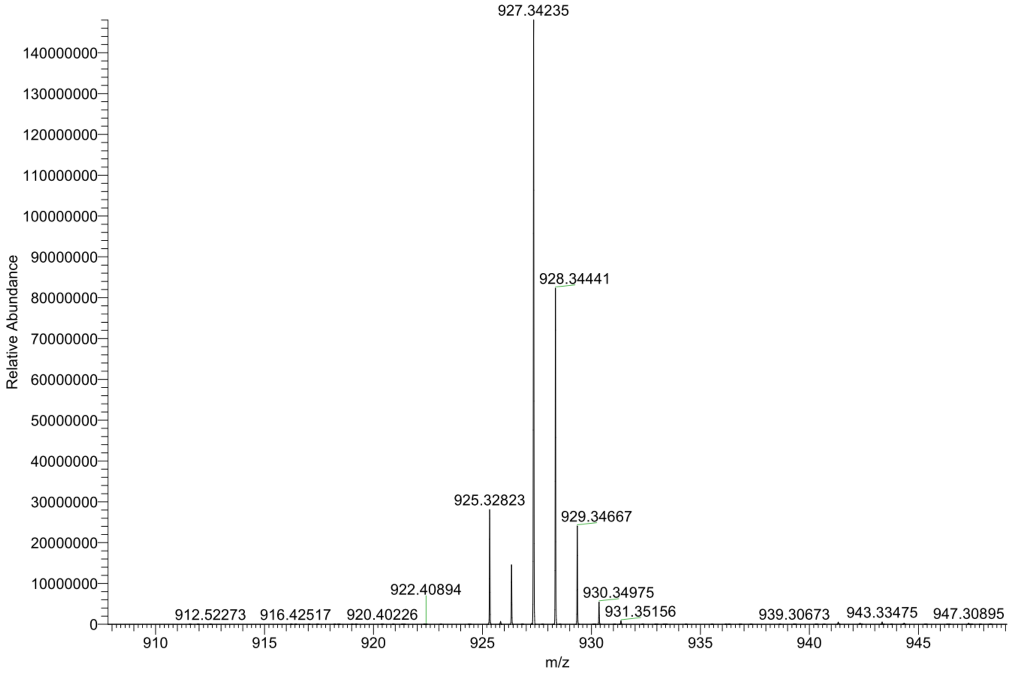

4-(1-(6-(3-(1-(21-((2-(2,6-dioxopiperidin-3-yl)-1,3-dioxoisoindolin-4-yl)amino)-21-oxo-3,6,9,12,15,18-hexaoxahenicosyl)-1H-1,2,3-triazol-4-yl)phenyl)-4-methylpyridin-3-yl)-1H-1,2,3-triazol-4-yl)benzoic acid (**10**)

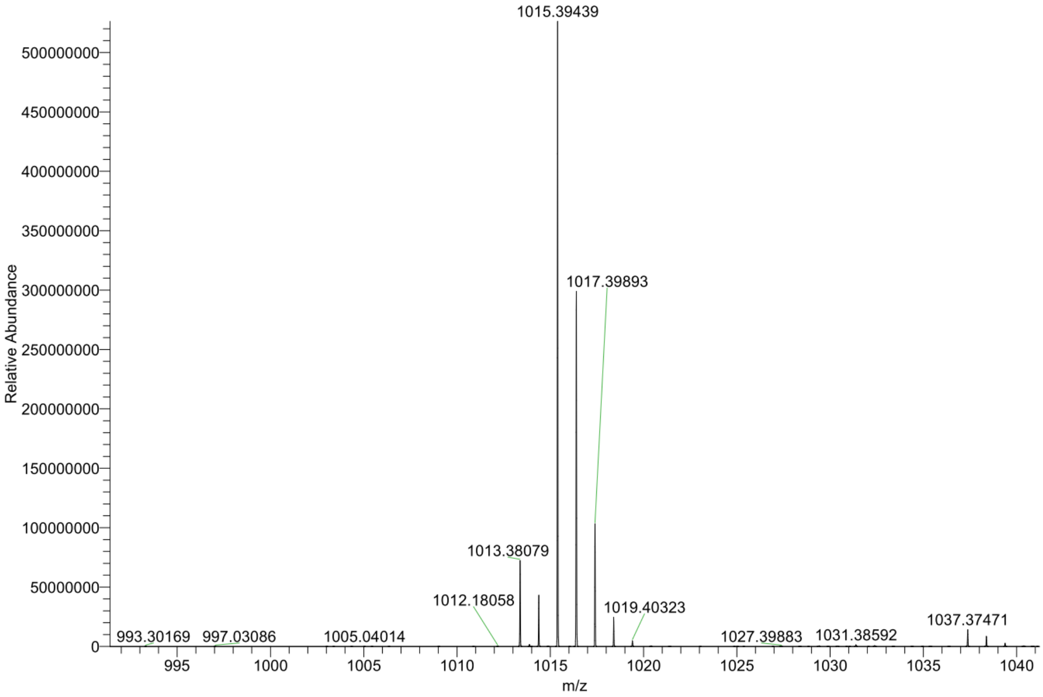

4. HPLC Traces

4-(1-(6-(3-(1-(4-(((S)-1-((2S,4R)-4-hydroxy-2-((4-(4-methylthiazol-5-yl)benzyl)carbamoyl)pyrolidin-1-yl)-3,3-dimethyl-1-oxobutan-2-yl)amino)-4-oxobutyl)-1H-1,2,3-triazol-4-yl)phenoxy)-4-methylpyridin-3-yl)-1H-1,2,3-triazol-4-yl)benzoic acid (**1**)

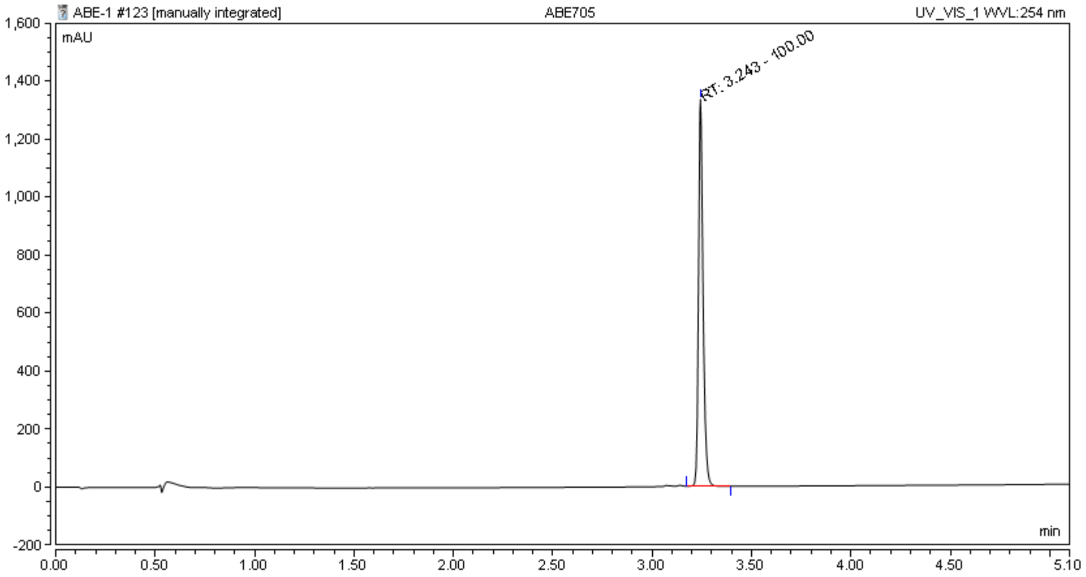

4-(1-(6-(3-(1-(4-(((S)-1-((2S,4R)-4-hydroxy-2-((4-(4-methylthiazol-5-yl)benzyl)carbamoyl)pyrolidin-1-yl)-3,3-dimethyl-1-oxobutan-2-yl)amino)-4-oxobutyl)-1H-1,2,3-triazol-4-yl)phenyl)-4-methylpyridin-3-yl)-1H-1,2,3-triazol-4-yl)benzoic acid (**3**)

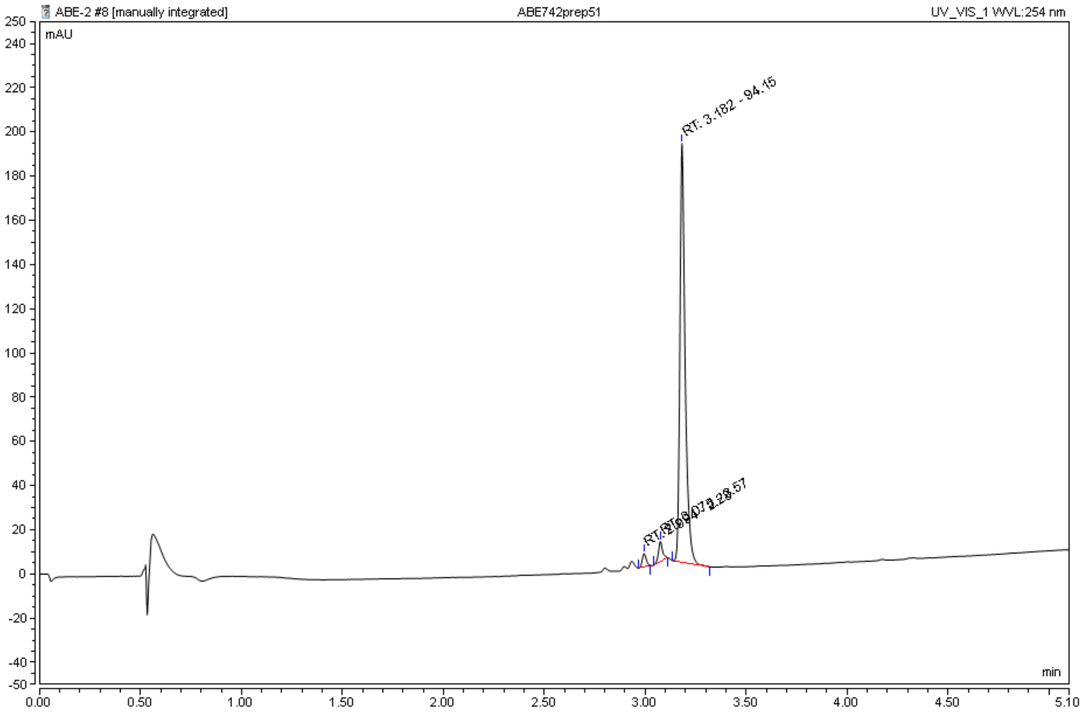

4-(1-(6-(3-(1-(2-(2-(3-(((S)-1-((2S,4R)-4-hydroxy-2-((4-(4-methylthiazol-5-yl)benzyl)carbamoyl) pyrolidin-1-yl)-3,3-dimethyl-1-oxobutan-2-yl)amino)-3-oxopropoxy)ethoxy)ethyl)-1H-1,2,3-triazol-4-yl)phenyl)-4-methylpyridin-3-yl)-1H-1,2,3-triazol-4-yl)benzoic acid (**5**)

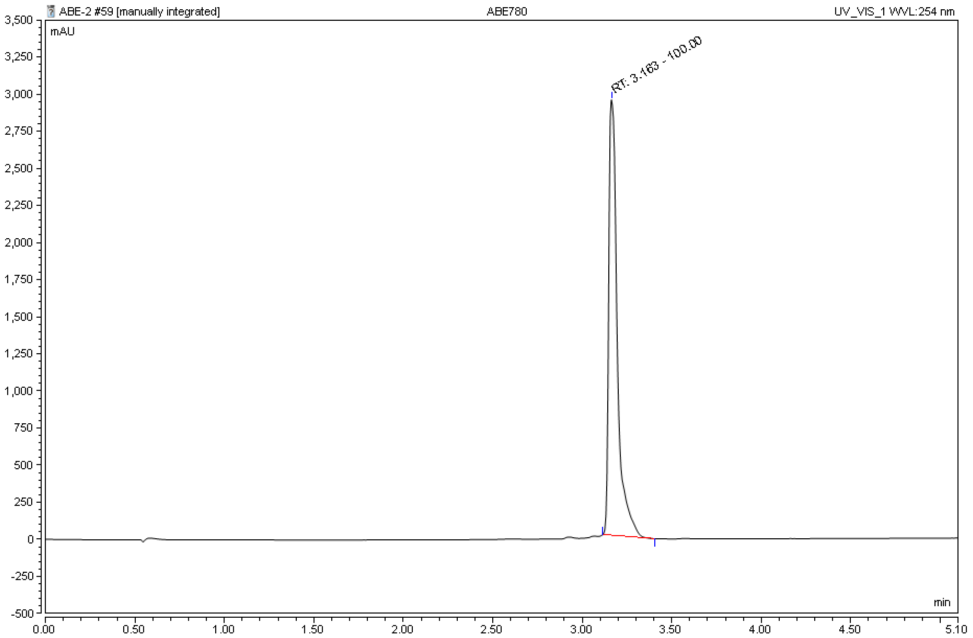

4-(1-(6-(3-(1-((S)-17-((2S,4R)-4-hydroxy-2-((4-(4-methylthiazol-5-yl)benzyl)carbamoyl)pyrolidine-1-carbonyl)-18,18-dimethyl-15-oxo-3,6,9,12-tetraoxa-16-azanonadecyl)-1H-1,2,3-tria zol-4-yl)phenyl)-4-methylpyridin-3-yl)-1H-1,2,3-triazol-4-yl)benzoic acid (**7**)

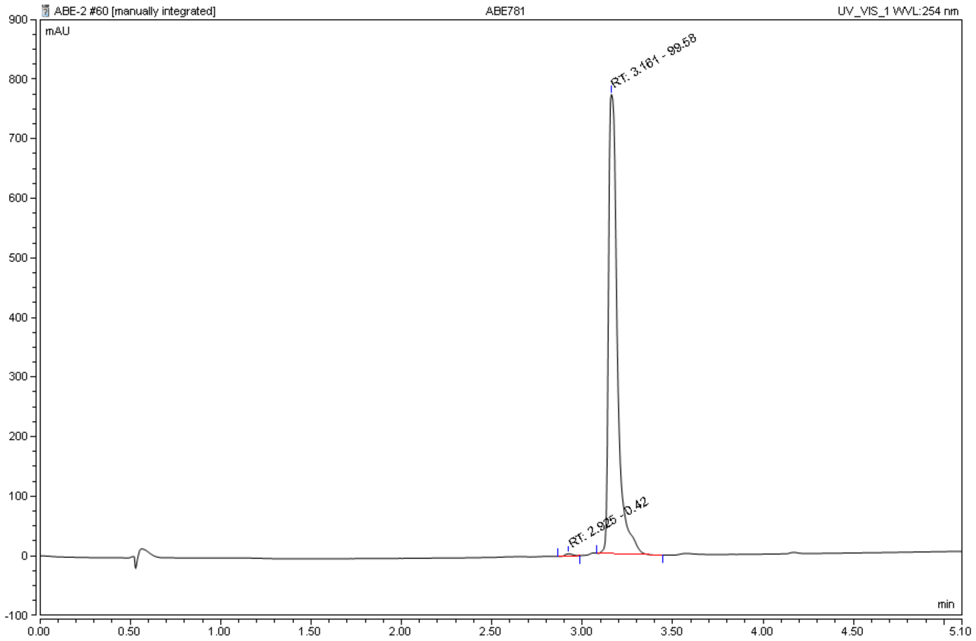

4-(1-(6-(3-(1-((S)-23-((2S,4R)-4-hydroxy-2-((4-(4-methylthiazol-5-yl)benzyl)carbamoyl)pyrolidine-1-carbonyl)-24,24-dimethyl-21-oxo-3,6,9,12,15,18-hexaoxa-22-azapentacosyl)-1H-1,2,3-triazol-4-yl)phenyl)-4-methylpyridin-3-yl)-1H-1,2,3-triazol-4-yl)benzoic acid (**9**)

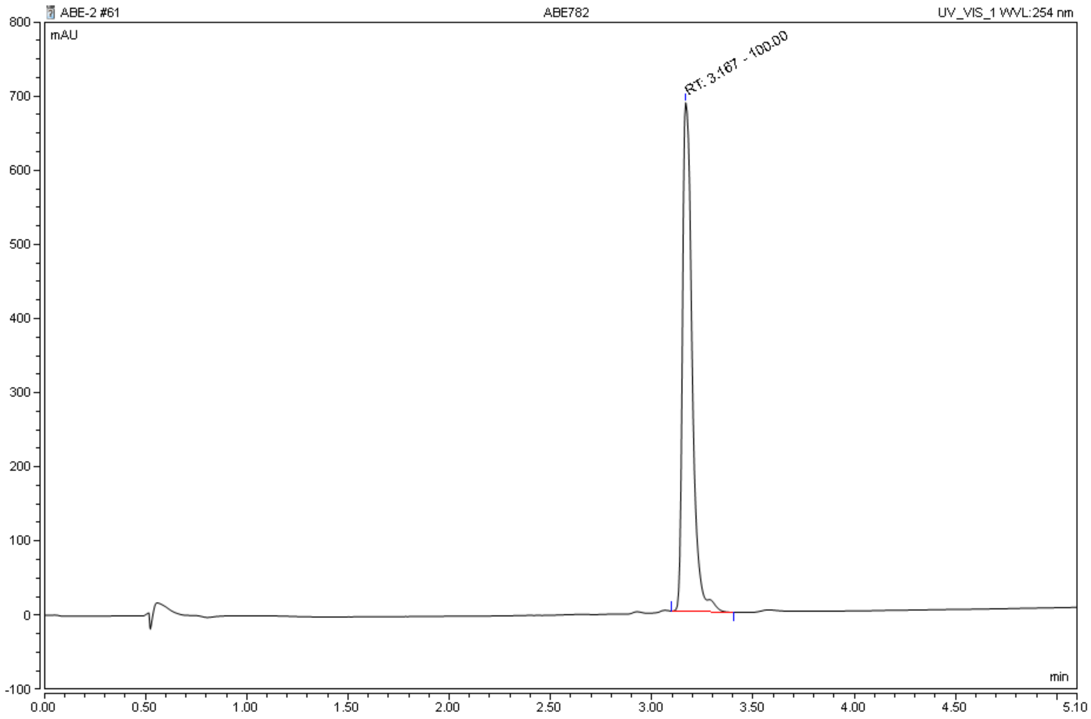

4-(1-(6-(3-(1-(4-((2-(2,6-dioxopiperidin-3-yl)-1,3-dioxoisoindolin-4-yl)amino)-4-oxobutyl)-1H-1,2,3-triazol-4-yl)phenoxy)-4-methylpyridin-3-yl)-1H-1,2,3-triazol-4-yl)benzoic acid (**2**)

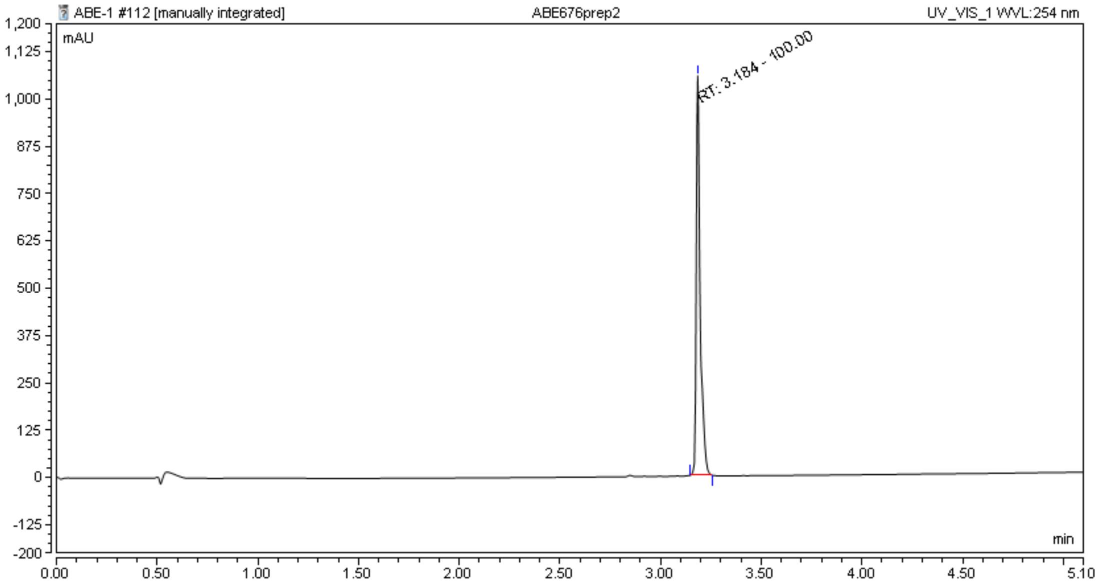

4-(1-(6-(3-(1-(4-((2-(2,6-dioxopiperidin-3-yl)-1,3-dioxoisoindolin-4-yl)amino)-4-oxobutyl)-1H-1,2,3-triazol-4-yl)phenyl)-4-methylpyridin-3-yl)-1H-1,2,3-triazol-4-yl)benzoic acid (**4**)

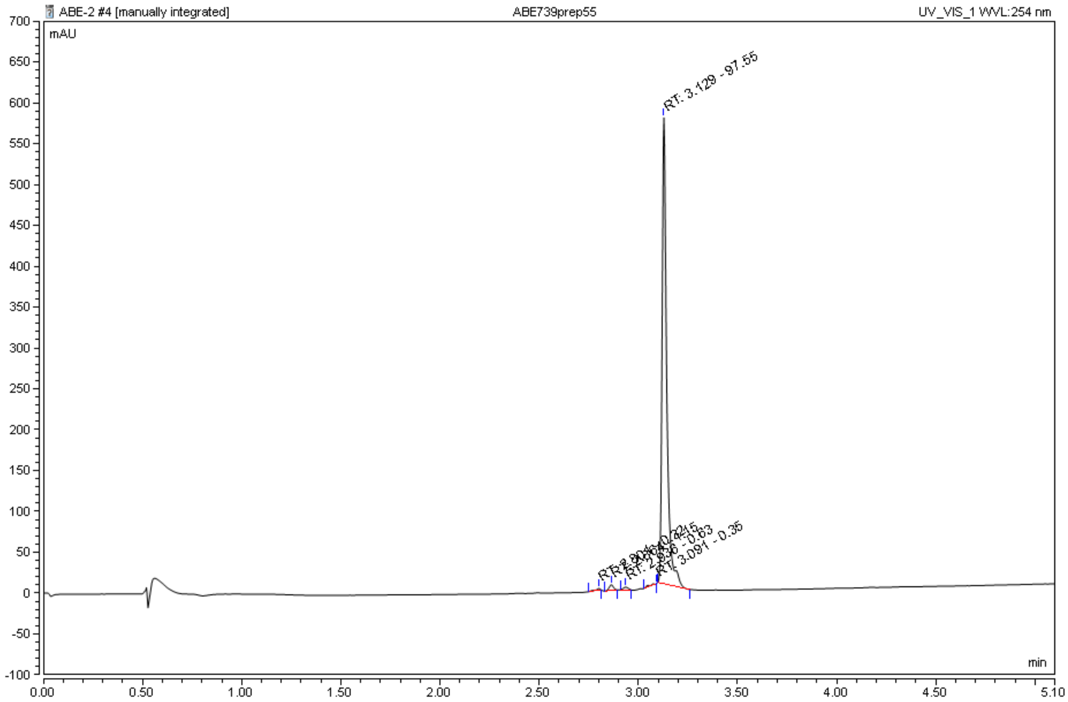

4-(1-(6-(3-(1-(2-(2-(3-((2-(2,6-dioxopiperidin-3-yl)-1,3-dioxoisoindolin-4-yl)amino)-3-oxopropoxy) ethoxy)ethyl)-1H-1,2,3-triazol-4-yl)phenyl)-4-methylpyridin-3-yl)-1H-1,2,3-triazol-4-yl) benzoic acid (**6**)

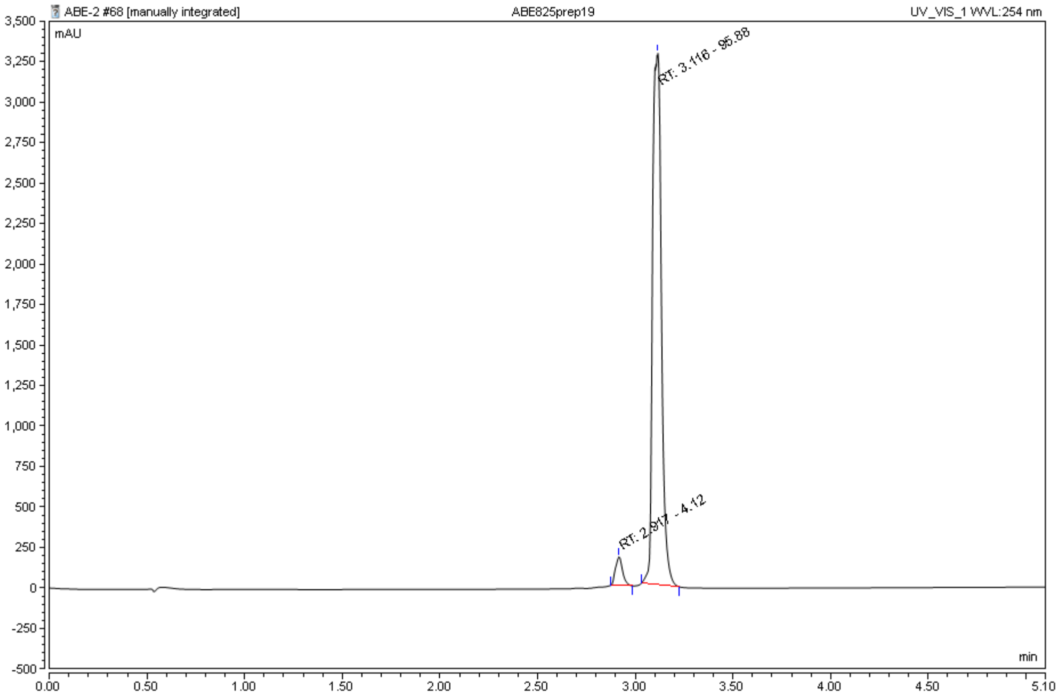

4-(1-(6-(3-(1-(15-((2-(2,6-dioxopiperidin-3-yl)-1,3-dioxoisoindolin-4-yl)amino)-15-oxo-3,6,9,12-tetraoxapentadecyl)-1H-1,2,3-triazol-4-yl)phenyl)-4-methylpyridin-3-yl)-1H-1,2,3-triazol-4-yl)benzoic acid (**8**)

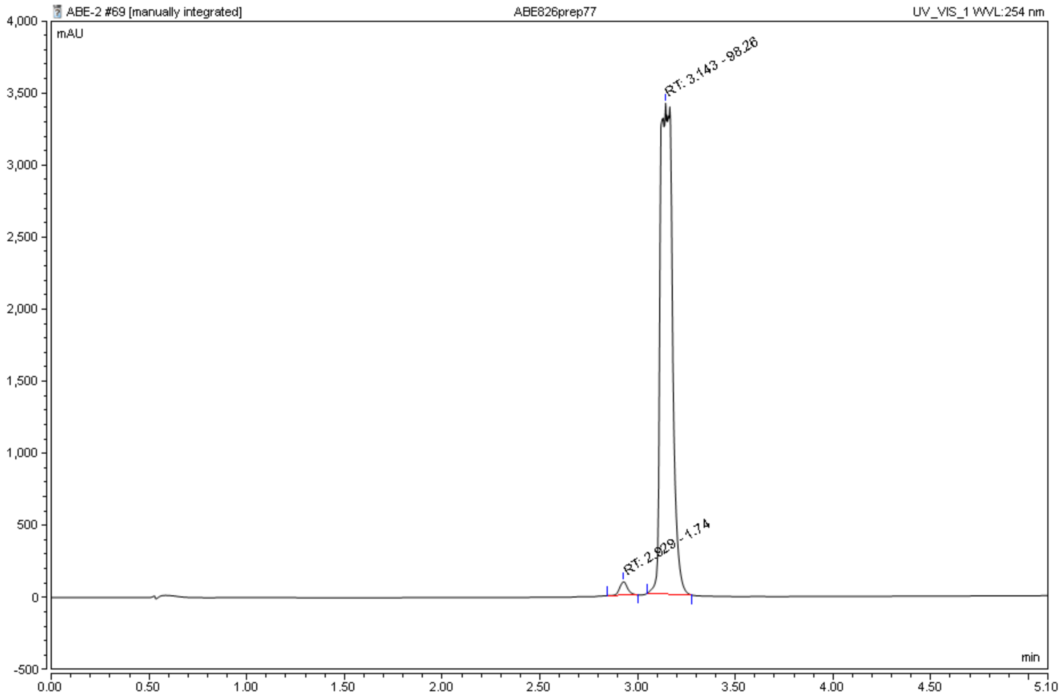

4-(1-(6-(3-(1-(21-((2-(2,6-dioxopiperidin-3-yl)-1,3-dioxoisoindolin-4-yl)amino)-21-oxo-3,6,9,12,15,18-hexaoxahenicosyl)-1H-1,2,3-triazol-4-yl)phenyl)-4-methylpyridin-3-yl)-1H-1,2,3-triazol-4-yl)benzoic acid (**10**)

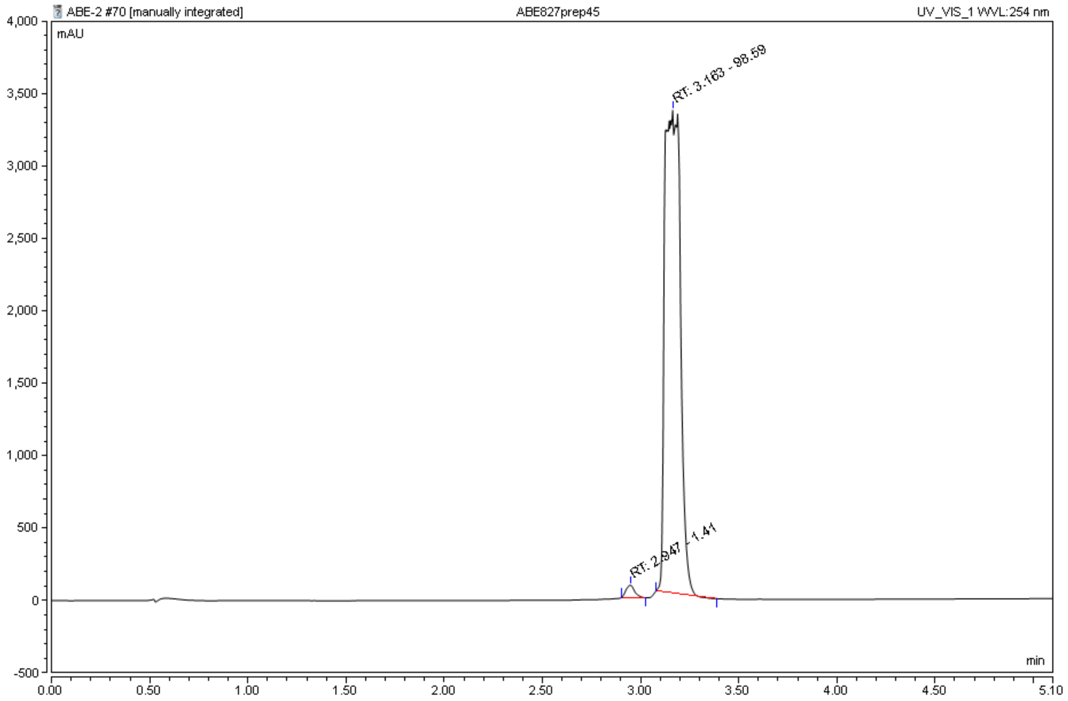

5. References

[1] A. Berwanger, S. C. Stein, A. M. Kany, M. Gartner, B. Loretz, C.-M. Lehr, A. K. H. Hirsch, T. F. Schulz, M. Empting, "Disrupting Kaposi's Sarcoma-Associated Herpesvirus (KSHV) Latent Replication with a Small Molecule Inhibitor", *J. Med. Chem.* **2023**, *66*, 10782–10790.

[2] D. Kundu, M. Tripathy, P. Maity, B. C. Ranu, "Cobalt-catalyzed intermolecular C(sp(2))-O cross-coupling", *Chemistry* **2015**, *21*, 8727–8732.

[3] W. Li, C. Gao, L. Zhao, Z. Yuan, Y. Chen, Y. Jiang, "Phthalimide conjugations for the degradation of oncogenic PI3K", *Eur. J. Med. Chem.* **2018**, *151*, 237–247.

[4] H. J. Bailey et al., "An engineered cereblon optimized for high-throughput screening and molecular glue discovery", *Cell Chem. Biol.* **2025**, *32*, 363-376.e10.

[5] D. L. Buckley, I. van Molle, P. C. Gareiss, H. S. Tae, J. Michel, D. J. Noblin, W. L. Jorgensen, A. Ciulli, C. M. Crews, "Targeting the von Hippel-Lindau E3 ubiquitin ligase using small molecules to disrupt the VHL/HIF-1α interaction", *Journal of the American Chemical Society* **2012**, *134*, 4465–4468.

[6] P. Kirsch, V. Jakob, K. Oberhausen, S. C. Stein, I. Cucarro, T. F. Schulz, M. Empting, "Fragment-Based Discovery of a Qualified Hit Targeting the Latency-Associated Nuclear Antigen of the Oncogenic Kaposi's Sarcoma-Associated Herpesvirus/Human Herpesvirus 8", *J. Med. Chem.* **2019**, *62*, 3924–3939.

[7] J. Hellert et al., "A structural basis for BRD2/4-mediated host chromatin interaction and oligomer assembly of Kaposi sarcoma-associated herpesvirus and murine gammaherpesvirus LANA proteins", *PLoS Pathog.* **2013**, *9*, e1003640.
